# Fast-forward prediction of lattice Boltzmann dynamics with physics-informed neural operators

**DOI:** 10.1038/s41467-026-75730-1

**Published:** 2026-07-29

**Authors:** Xiao Xue, Marco F. P. ten Eikelder, Mingyang Gao, Xiaoyuan Cheng, Yiming Yang, Yi He, Shuo Wang, Sibo Cheng, Yukun Hu, Peter V. Coveney

**Affiliations:** 1 Centre for Computational Science, University College London, London, UK; 2 Institute for Mechanics, Computational Mechanics Group, Technical University of Darmstadt, Darmstadt, Germany; 3 Dynamic Systems Lab, University College London, London, UK; 4 Department of Statistical Science, University College London, London, UK; 5 Department of Heat Transfer and Fluid Dynamics, Netherlands Organisation for Applied Scientific Research, Rijswijk, Netherlands; 6 Centre d’Enseignement et de Recherche en Environnement Atmosphérique (CEREA), École des Ponts and EDF R&D, Institut Polytechnique de Paris, Île-de-France, France; 7 Computational Science Laboratory, Institute for Informatics, University of Amsterdam, Amsterdam, the Netherlands; 8 Centre for Advanced Research Computing, University College London, London, UK

**Keywords:** Statistical physics, Computational science

## Abstract

The lattice Boltzmann equation (LBE), rooted in kinetic theory, captures complex flow behaviour by evolving single-particle distribution functions (PDFs), but its explicit time-stepping makes large-scale simulation computationally intensive. Here we introduce a physics-informed neural operator framework that predicts the LBE evolution over large time jumps without performing step-by-step forward integration, bypassing the need to solve the collision kernel explicitly. The model embeds intrinsic moment-matching constraints and global equivariance of the distribution field, preserving the kinetic structure of the underlying system. The framework is discretization-invariant: models trained on coarse-grained PDFs perform inference on finer grids even when the relaxation time differs between resolutions. It is also agnostic to the lattice Boltzmann formulation, allowing the same architecture to be reused across different kinetic datasets. Across von Kármán vortex shedding, ligament breakup, and bubble adhesion, the framework offers a robust data-driven pathway for accelerating the lattice Boltzmann based dynamical systems.

## Abstract (web summary)

The lattice Boltzmann method models complex flows through particle distribution functions but is limited by small time steps. The authors propose a physics-informed neural operator that advances these functions over large time steps, greatly accelerating simulations without the need to handle collisions explicitly.

## Introduction

Mathematical models based on partial differential equations are essential tools for simulating dynamical systems across the sciences^1–4^. While these macroscopic models underpin much of our understanding of fluid dynamics, plasma physics, and climate science, they often struggle to model microscopic interactions, which are essential for accurately modelling multiphase or multiscale phenomena^5–7^. Kinetic theory offers a natural framework to bridge microscopic and macroscopic scales by describing the evolution of single-particle distribution functions (PDFs) ^8–10^. The lattice Boltzmann (LB) equation has been applied to model a wide range of complex physical systems^11–15^, including multiphase flows^16–24^, turbulent flows^25–28^, as well as biomedical applications such as blood flow and hemodynamics in vascular networks^29–32^. Inspired by Grad’s approach^33,34^, Shan and his coauthors^35^ have demonstrated a promising systematic method to approximate the collision kernel using higher-order Hermite expansions, which can represent the Navier-Stokes equations, the Burnett equations, and many others. Beyond its application in hydrodynamics on both the macroscale^12,36^ and microscale^37–39^, the lattice Boltzmann equation also connects to relativistic fluids^40,41^, thereby extending its relevance to cosmology and plasma physics^42^ at the kinetic level. Despite its success, solving the LBE remains computationally intensive.

As a result, alternative approaches are needed to overcome the computational challenges associated with solving kinetic equations. Machine learning (ML) models, ranging from convolutional neural networks and recurrent architectures to more recent innovations such as transformers and diffusion models, have achieved strong performance in domains such as computer vision, natural language processing, and generative modelling^43–47^. Motivated by these advances, there is growing interest in applying ML techniques to scientific computing tasks. In particular, ML-based approaches have shown promise in fluid mechanics, where they are used for prediction, surrogate modelling, and closure modelling of dynamical systems^48–52^.

Within fluid mechanics, ML models are typically categorised into two main approaches. The first, referred to as “hybrid ML”, incorporates machine-learned components into conventional numerical solvers by partially replacing specific terms or submodels in the governing evolution equations ^53–56^. Examples of this approach include the development of turbulent subgrid-scale (SGS)^53,54,57–60^ and near-wall models^55,56,61^. The second approach, referred to as “standalone ML”, aims to learn the entire numerical solution procedure with a data-driven model that directly predicts the system evolution^62^. A prominent example of this approach is the class of neural operators, which aim to learn mappings from input fields to output fields and have been successfully applied to a range of PDE models^47,62–66^.

Despite recent progress in scientific ML, its application to the LBE remains limited. Most LB-focused ML approaches fall under the “hybrid” paradigm, owing to the presence of the collision kernel in the LB formulation. These methods typically aim to learn the collision term. For example, Bedrunka et al.^67^ optimised parameters of multiple-relaxation-time (MRT) collision kernels, while Corbetta et al.^52,68^ developed a neural network architecture to approximate the discrete Bhatnagar-Gross-Krook (BGK) collision kernel by incorporating mathematical and physical constraints. Extending these ideas, Ortali et al.^69^ introduced a three-dimensional neural collision kernel trained on direct numerical simulation (DNS) data to model LB-based subgrid-scale interactions. However, these approaches are typically limited to single-timestep forward predictions and remain constrained by the fine temporal resolution required to resolve collision dynamics. Consequently, developing methods that bypass time-step constraints and model the forward operator without explicitly learning the collision kernel remains an early and largely unexplored area of research. Implicit and semi-implicit treatments of kinetic equations, including penalty methods for the Boltzmann equation^70^, implicit Fokker–Planck formulations^71^, implicit BGK and Lenard–Bernstein solvers^72,73^, and implicit discrete-ordinate transport schemes form an important line of related work. These methods modify the time-stepping loop to improve stability or stiffness treatment. However, both collision-kernel learning and implicit kinetic solvers remain tied to advancing the kinetic equation through the underlying temporal discretisation. Consequently, methods that learn a reusable multi-step forward map for the distribution-functions remain largely unexplored. Lat-Net^74^, for instance, approximates LBE dynamics in a compressed latent space, making it difficult to enforce local physical conservation laws and kinetic constraints, which can lead to degraded accuracy in out-of-distribution scenarios, thereby limiting its practical applicability in physics-informed modelling.

**Fig. 1:**
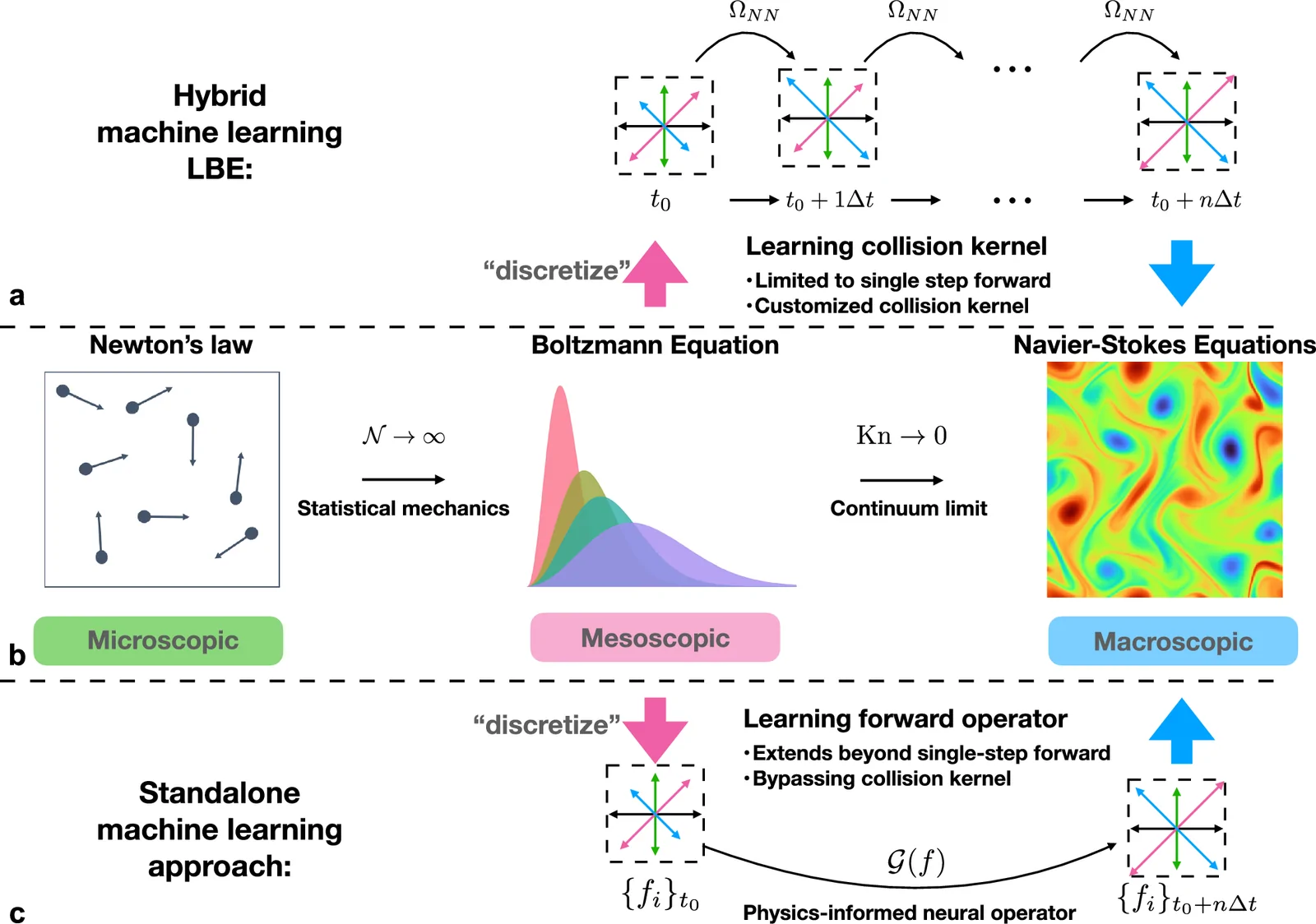
A multiscale framework for lattice Boltzmann equation encompassing microscopic, mesoscopic, and macroscopic perspectives. Panel **a** outlines the kinetic data-driven hybrid ML approach for LBE, where neural networks are used to learn or enhance collision kernels in the lattice Boltzmann equation, a discrete form of the Boltzmann equation. Panel **b** illustrates molecular dynamics governed by Newton’s laws, focusing on individual molecules. As the molecular count ${\mathcal{N}}$ grows, statistical mechanics emerges, transitioning to the mesoscopic scale described by the Boltzmann equation. In the continuum limit, where the Knudsen number Kn → 0, the system converges to the macroscopic hydrodynamic equations. Panel **c** proposes a physics-informed neural operator for LBE, denoted by ${\mathcal{G}}$, that learns the long-time forward evolution of the single-distribution function fields {*f*_*i*_}, enabling large time jumps without explicitly solving the collision kernel.

Here, we propose a physics-informed neural operator (PINO) framework tailored to the lattice Boltzmann equation, enabling fast-forward prediction of single-particle distribution functions while preserving meso-to-macroscale consistency, without relying on explicit learning of the collision kernel (Fig. 1). Our goal is to construct a surrogate for the kinetic time advancement that is intrinsic to the lattice Boltzmann equation. Predicting the discrete single particle distribution functions preserves the full non-equilibrium content of the kinetic description. Importantly, as highlighted in Shan’s work^35^, the kinetic representation can encode physical effects beyond those captured by macroscopic PDEs. When only low-order macroscopic moments are available, reconstructing the PDFs becomes a non-unique and often ill-posed inverse problem. The macroscopic fields do not contain sufficient information to uniquely determine the non-equilibrium components of the PDFs, and any missing or incomplete moment information leads to reconstruction errors. These errors directly contaminate the non-equilibrium stresses that drive LBE dynamics, and even small inaccuracies can cause numerical instabilities when the reconstructed PDFs are reinserted into a lattice Boltzmann solver for further time integration if needed. By learning the PDFs directly, the proposed framework avoids reconstruction ambiguity and remains compatible with the full kinetic structure of the LBE, allowing the learned distributions to be stably advanced by downstream LB solvers if needed. This distribution-function-level objective is distinct from learning macroscopic closure quantities, such as stress, effective viscosity, or phase-field fluxes. Although such closures are valuable in hydrodynamic solvers, they do not specify the full set of distribution functions required for subsequent collision, streaming, boundary-condition, or forcing operations. We therefore learn the distribution functions directly and recover macroscopic or closure-related observables through moment operations when needed.

To obtain physical consistency, we incorporate domain-specific inductive biases into the training process, including local moment-matching constraints and global equivariance of the full distribution function fields, where the global symmetry arises from the underlying local lattice structure. We primarily focus on the U-shaped Neural Operator (UNO) architecture^75^ due to its memory efficiency, which makes it well-suited for large-scale kinetic simulations. While our main results are based on UNO, ablation studies confirm that the Fourier Neural Operator (FNO)^62^ exhibits similar trends under the same physics-informed training regime. These structural constraints enable the model to accurately capture the complex behaviour of the underlying mesoscopic dynamical system while preserving key macroscopic physical properties. We demonstrate that the evolution of single-particle distribution functions can be modelled without explicitly resolving every intermediate stream–collide step, while still respecting the discrete lattice structure through the learned kinetic variables and symmetry-aware constraints. The same trained surrogate can also be evaluated on finer grids within the same lattice family, indicating that it captures the underlying kinetic evolution rather than merely memorising grid-specific patterns. Our approach has been tested on both D2Q9 and D3Q19 lattice models, using a variety of collision operators including the Bhatnagar-Gross-Krook (BGK) model with a Smagorinsky LES closure, the MRT model, and a phase-field formulation of the LBE. This flexibility enables the prediction of complex flow scenarios such as von Kármán vortex shedding, ligament breakup, and bubble adhesion. The purpose of these three examples is to demonstrate that the proposed framework applies consistently across distinct LBE formulations and collision models, rather than to suggest that the simulated flows lie outside the continuum regime. The method’s flexibility with respect to lattice configurations and collision models highlights its broad applicability across diverse kinetic systems.

## Results

### Physics-informed neural operator framework for the lattice Boltzmann equation

The lattice Boltzmann equation can be seen as a velocity-discretised form of the Boltzmann kinetic equation^12^, where the continuous velocity variable is simplified by a defined finite set of *Q* discrete velocities ${\left\{{{\bf{c}}}_{i}\right\}}_{i=0}^{Q-1}$. These discrete velocities **c**_*i*_ are fictitious vectors defined by the lattice and represent the possible directions for particle streaming; they do not correspond to the physical (macroscopic) velocity of the fluid. This leads to a system of *Q* single-particle distribution functions *f*_*i*_ = *f*_*i*_(**x**, *t*), where each ${f}_{i}:{{\mathbb{R}}}^{d}\times {\mathbb{R}}\to {\mathbb{R}}$ describes the density of particles moving with velocity **c**_*i*_ at position ${\bf{x}}\in {{\mathbb{R}}}^{d}$ being the spatial dimension and time $t\in {\mathbb{R}}$:

1 $${\partial }_{t}\,{f}_{i}({\bf{x}},t)+{{\bf{c}}}_{i}\cdot \nabla {f}_{i}({\bf{x}},t)=\Omega ({\left\{{f}_{j}\right\}}_{j=0}^{Q-1}({\bf{x}},t))+{F}_{i}({\bf{x}},t).$$

The left-hand side of Eq.(1) corresponds to the streaming step, which accounts for the propagation of particles, while the first term on the right-hand side represents collisions that redistribute particles among the discrete velocities, where *Ω* is the collision operator and the second term accounts for external forcing *F*. Throughout this paper, “kinetic” refers to the distribution-function description in discrete phase space: the neural operator advances *f*_*i*_(**x**, *t*) over the lattice coordinates and velocities, rather than relying on macroscopic moments reconstructed at the Navier–Stokes level.

Conventional numerical solvers for the LBE are constrained by the explicit treatment of the collision operator, which governs local interactions and facilitates information exchange between neighbouring lattice cells. In contrast to the traditional perspective that decomposes the dynamics into streaming and collision steps at the level of individual cells, we adopt a functional perspective. Specifically, we treat each discrete *f*_*i*_(**x**, *t*) of the particle distribution function as a spatially structured field over the entire domain. The goal is to learn a forward operator that predicts the evolution of the full set {*f*_*i*_} over an extended time interval. Let *t* be the initial time and $t={t}^{{\prime} }+n\Delta t$ the target time. When *n* is large, local interactions become decorrelated, and the evolution becomes increasingly global. We define a neural operator ${\mathcal{G}}$ as:

2a $$G\,:\,A\,{\to} \,U,$$

2b $${\mathcal {A}}=\left\{\left\{{f}_{i}(\cdot,t)\right\}_{i=0}^{Q-1}| t \in {T}_{\mathrm{in}}\right\},$$

2c $${U}=\left\{\left\{{f}_{i}(\cdot,t)\right\}_{i=0}^{Q-1}| t\in {T}_{\mathrm{out}}\right\},$$

where ${\mathcal{A}}$ represents the collection of distribution functions {*f*_*i*_} across all discrete velocities *i* = 0, …, *Q* − 1 over an input time window ${{\mathcal{T}}}_{{\rm{in}}}\subset {\mathbb{R}}$, and ${\mathcal{U}}$ represents their corresponding evolution over the output time window ${{\mathcal{T}}}_{{\rm{out}}}\subset {\mathbb{R}}$. Each *f*_*i*_( ⋅ , *t*) is treated as a spatial field belonging to a function space Ψ. The global operator ${\mathcal{G}}$ is trained to map ${\mathcal{A}}$ to ${\mathcal{U}}$, encapsulating the combined effects of transport and collision dynamics over long time horizons. When the output interval ${{\mathcal{T}}}_{{\rm{out}}}$ lies far behind the input interval ${{\mathcal{T}}}_{{\rm{in}}}$, local interactions are decorrelated and the evolution becomes increasingly global.

**Fig. 2:**
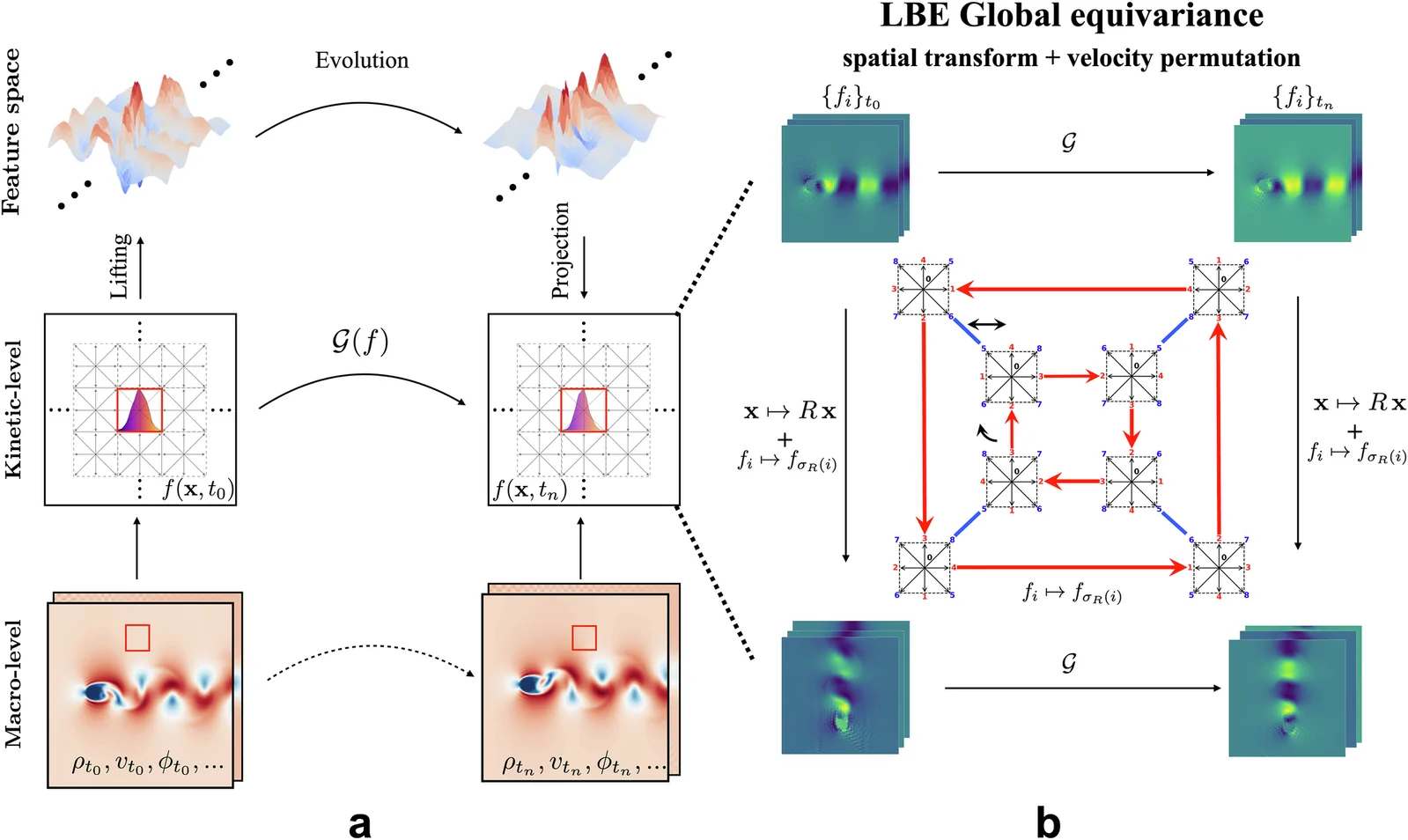
Physics-informed neural operator framework and global equivariance for the lattice Boltzmann equation. Panel **a**, kinetic data-driven framework. The operator ${\mathcal{G}}$ learns the long-time forward evolution of the distribution functions {*f*_*i*_}. Starting from the PDF fields *f*(**x**, *t*_0_), the input is lifted into a feature space, evolved by the neural operator, and projected back to obtain *f*(**x**, *t*_*n*_), where *t*_*n*_ = *t*_0_ + *nΔt*. Macroscopic observables such as density *ρ*, velocity **v**, and phase field *ϕ* are recovered via moment maps (dotted arrows). Panel **b**, Global equivariance under lattice symmetries. For a group action **R** (rotation or reflection in *D*_4_), the input field transforms as ${[{\bf{R}}\cdot f]}_{i}({\bf{x}},t)={f}_{{\sigma }_{R}(i)}({{\bf{R}}}^{-1}{\bf{x}},t)$, combining a spatial transformation with permutation of velocity channels. The equivariance property ${\mathcal{G}}({\bf{R}}\cdot f)={\bf{R}}\cdot {\mathcal{G}}(f)$ ensures that predictions remain consistent with the discrete symmetries of the lattice Boltzmann formulation.

To approximate this forward operator, we employ physics-informed neural operators that generalise across spatial discretizations and enable inference on unseen grid resolutions. A key challenge in learning lattice Boltzmann dynamics is preserving the connection between mesoscopic distribution functions and the macroscopic physical quantities they encode, while also ensuring consistency with the underlying lattice structure of the system. To address this, we incorporate LBE-specific inductive biases into the training loss, including local moment-matching constraints to preserve meso-to-macroscale consistency and global equivariance of the distribution function fields under the action of the discrete symmetry group induced by the underlying local lattice structure. These constraints guide the learned operator to remain physically consistent across scales and symmetries. Figure 2a provides an overview of the kinetic data-driven framework. We begin by generating PDF data through lattice Boltzmann simulations. The distribution fields *f*(**x**, *t*_0_) are lifted into a feature space, evolved through the neural operator ${\mathcal{G}}$, and projected back to obtain *f*(**x**, *t*_*n*_). In this work, we mainly examine the U-shaped Neural Operator^75^, which implicitly learns this evolution via a data-driven composition of local and global operators, yielding approximate solutions at the PDF level. From the predicted distributions, macroscopic observables such as density *ρ*, velocity **v**, and phase field *ϕ* are recovered through moment maps (see Section IV B). A detailed description of the unit conversion from lattice units (LU) to physical units is provided in Supplementary Information Section S2.

Figure 2**b** illustrates the role of discrete symmetry groups (e.g., the dihedral group *D*_4_ in D2Q9) in enhancing global equivariance, which requires applying both a spatial transformation **R**: **x** ↦ **Rx** and a velocity-channel permutation ${f}_{i}\mapsto {f}_{{\sigma }_{R}(i)}$. This property is formally expressed as ${\mathcal{G}}({\bf{R}}\cdot f)={\bf{R}}\cdot {\mathcal{G}}(f)$ and ensures that the learned operator respects the discrete symmetries of the lattice Boltzmann formulation (see “Methods” C and Supplementary Information Section S1 for the full proof). Training is performed with two loss formulations: a standard mean squared error (MSE) loss for data fidelity, and a physics-informed variant (MSE+Phys) that augments MSE with local moment-matching and global equivariance constraints (see Eq. (3)). Details of the network architecture are given in Supplementary Information Section S4.

We have assessed our proposed PINO for LBE framework with several applications of increasing complexity, including different physical phenomena governed by various LB models in 2D and 3D. In doing so, our framework has established fundamental principles for handling LB models that can be applied to general hydrodynamics set-ups, ensuring their broad applicability and reliability. Our evaluation begins with the von Kármán vortex street problem and progresses to more complex scenarios, including the breakup of a multiphase liquid cylinder and bubble adhesion on a solid cylinder. A systematic overview of these problems, including visualisations of the physical input-output mappings, is presented in Table 1. To assess whether learning the full distribution function set {*f*_*i*_} is necessary, we also compare against macroscopic-only (hereafter Macro-only) models trained on the same datasets and evaluated on the relevant hydrodynamic observable. These comparisons are not intended to show that PDF prediction is universally more accurate for every scalar observable; rather, they quantify the trade-off between macroscopic accuracy and retaining the complete kinetic state required for an LB surrogate. To validate the robustness of our framework, we also perform an ablation study by replacing the UNO backbone with FNO. Additional training details and extended results are provided in the Supplementary Information. All error metrics are described in Supplementary Information Section S8. The dataset is available from Xue^76^.

**Table 1:** A schematic overview of kinetic data-driven examples is presented

Physical Problems	Lattice Models	Training Dataset	Test Model Output	Input Snapshot	Output Snapshot
von Kármán vortex streets	D3Q19	${\rm{Re}}=180$, *R*_*c*_ = 3 LU, prediction jumping time: 50*Δt*, *N*_train_ = 1600	*G*(*x*, *t*_in_) → *f*(**x**, *t*_out_), *t*_in_ ∈ [*Δ*50*t*_coll_: *Δ*250*t*_coll_], *N*_val_ = 200, *N*_test_ = 200		
Ligament breakup	D3Q19	*x*_*c*_ ∈ [14: 2: 34], *ϕ*_*s*_ ∈ [0: 0.04: 2), prediction jumping time: 10*Δt*, *N*_train_ = 352	*G*(*x*, *t*_0_ = 0) → *f*(**x**, t^*^), t^*^ ∈ [0: 1], *N*_val_ = 44, *N*_test_ = 44		
Bubble adhesion	D2Q9	*x*_*d*_ ∈ [26: 1: 56], *y*_*d*_ ∈ [30: 1: 46), prediction jumping time: 300*Δt*, *N*_train_ = 396	*G*(*x*, *t*_0_ = 0) → *f*(**x**, t^*^), t^*^ ∈ [0: 1], *N*_val_ = 50, *N*_test_ = 50		

The corresponding lattice models indicate which LB model was used to generate the data during the LB simulation. The model input and output data illustrate the training and testing datasets employed. Additionally, the input and output snapshots provide examples of the instantaneous inputs and the predicted outcomes analyzed in this study.

### Von Kármán vortex streets

**Fig. 3:**
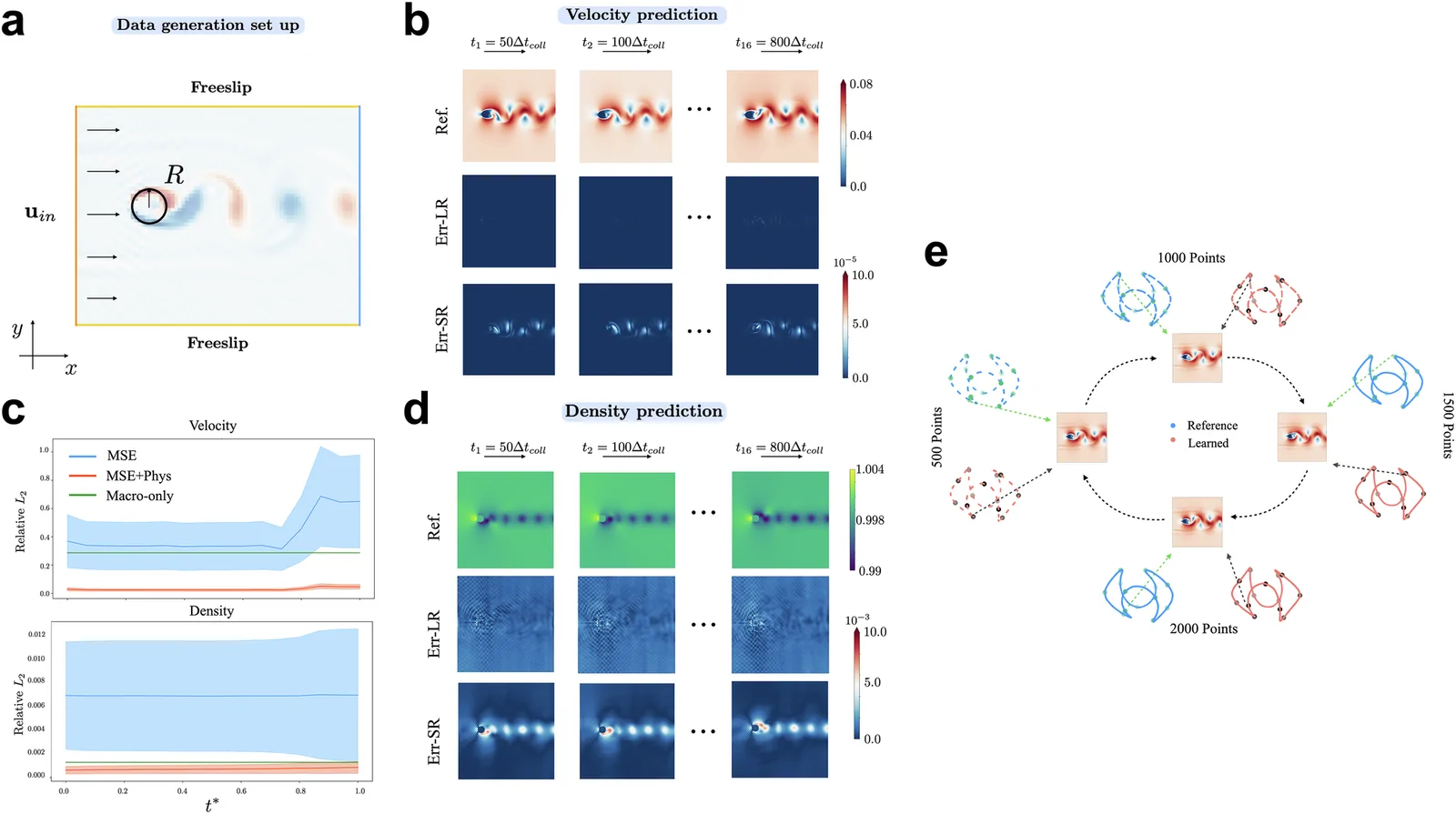
Von Kármán vortex streets prediction using the distribution-function data. The initial condition is randomly selected from a test dataset, and the prediction is generated autoregressively afterwards. Panel **a** provides an outline of the experimental setup. Panel **b** shows the velocity field evolution, where the top row displays the ground truth. The middle row displays the absolute error of the MSE+Phys prediction at low resolution (LR), and the bottom row shows the absolute error obtained by direct super resolution (SR) inference, without retraining the model. Panel **c** presents the relative *L*_2_ error for macroscopic velocity (top) and density (bottom) as a function of the dimensionless time t^*^, comparing the kinetic-driven and Macro-only prediction approaches. Solid lines correspond to MSE (blue), MSE+Phys (orange), and Macro-only (green) models; shaded bands represent 95% confidence intervals across *n* = 10 independently trained models. Panel **d** demonstrates the density evaluation over time. The top row exhibits the ground truth for the density field evolution. The middle row displays the absolute error of the MSE+Phys prediction at low resolution (LR), and the bottom row shows the absolute error obtained by direct super resolution (SR) inference, without retraining the model. Panel **e** shows the embedding orbits from kinetic data, comparing ground truth (blue) and learned embeddings (orange) across sample points from 500 to 2000 in time. Black and green dots highlight a snapshot of the von Kármán vortex street, illustrated by the vorticity plot with a blue-orange colour map.

As illustrated in Fig. 3a, the 2D domain incorporates several boundary conditions. At the inlet, we impose a uniform velocity profile of *u*_in_ = 0.01 LU along the *x* direction, where LU denotes lattice units. The outlet is defined by a pressure-free boundary condition^77–79^. Additionally, freeslip boundary conditions are applied to both the top and bottom sides to eliminate drag from the boundary in the simulation^78^. The radius of the cylinder is set to *R*_*c*_ = 3 LU. The Reynolds number, defined in the lattice Boltzmann framework as ${\rm{Re}}=\frac{{{\bf{u}}}_{{\rm{in}}}{R}_{c}}{\nu }$, with *ν* the kinematic viscosity in LU, is set to ${\rm{Re}}=180$. The cylinder boundary equips a no-slip boundary condition. The first row in Table 1 shows the dataset configuration and the model output. The purpose of this example is to demonstrate that our framework can effectively learn from the distribution-function dataset governed by Eq. (7), predict the evolution of the distribution functions without explicitly learning the collision kernel and perform self-regressive rollouts. We adopted an 80%–10%–10% split for training, validation, and testing, respectively. To assess the fidelity of our ML models^80–82^, the framework was trained independently 10 times and evaluated on a held-out test set of 200 samples (See Supplementary Information Section S5 and S6). In Fig. 3b, the top row shows the ground-truth fields at 160 × 160 based on LB solver^78^. The middle row presents the absolute error of the MSE+Phys prediction on the low-resolution (LR) grid (80 × 80), which is the resolution used for training. Each prediction step is *Δt*_*n*_ = 50*Δt*. The bottom row displays the absolute error at super resolution, obtained through direct inference of the same model on the 160 × 160 grid without any retraining. Across all frames, both the low-resolution and super resolution errors remain within 0 to 1 × 10^−4^, indicating that the learned operator preserves accuracy even when evaluated at a resolution twice as fine as the training grid. Also, for the super resolution dataset collision relaxation time differs from the LR value. This demonstrates that the model is able to capture the fine-scale with the kinetic data and smoothly across spatial resolutions and supports super resolution inference. In Fig. 3c, we present the relative *L*_2_ error in the macroscopic predictions obtained using the MSE and MSE+Phys loss functions. The plots show the evolution of the error over normalised time t^*^ = *t*/*t*_*end*_ with *t*_*end*_ = 6000*Δt* for velocity (top) and density (bottom), comparing models trained with standard MSE loss (blue) and physics-informed MSE+Phys loss (orange). Importantly, we also compare against training with macroscopic data alone (green). Shaded bands denote the 95% confidence interval across *n* = 10 independently trained models. The MSE+Phys kinetic formulation consistently outperforms both the MSE-only kinetic model and the macroscopic-only baseline, yielding lower mean error and reduced variance across the prediction horizon. This improvement stems from the richer single-cell information contained in the full set of kinetic distribution functions, which the macroscopic variables alone cannot provide under the same architecture.

The advantage of the PDF evolution becomes increasingly pronounced at later stages of the rollout, particularly for the velocity field, underscoring the stabilising influence of the physics-based constraints during predictions. Figure 3d shows the density evolution obtained from the distribution functions following Eq.(8). Due to the weak compressibility of LBE^12^, the predicted field captures minor density oscillations with the help of the MSE+Phys constraint, indicated by dark blue circles, which align well with the ground truth. The top row shows the reference density fields at 160 × 160, while the middle row displays the absolute error at the low-resolution, corresponding to the training resolution. The bottom row shows the absolute error at a grid size of 160 × 160, obtained by direct super resolution inference without retraining the model. Across all time instants, the absolute density error remains below 1 × 10^−2^, indicating that the learned operator achieves highly accurate macroscopic reconstruction from the distribution functions even when evaluated on a grid twice as fine as the training resolution.

It is important to note that all results presented in this paper are in dimensionless units, which can be easily converted to physical units, as described in Supplementary Information Section S2. The error evaluation for individual snapshots can be found in Supplementary Information Section S6. To better interpret the high-dimensional feature space, we employ t-SNE (t-distributed Stochastic Neighbour Embedding)^83^, an unsupervised dimensionality reduction technique, to project the feature space data into three-dimensional points. The results presented in Fig. 3e use blue to represent the reference data and red to denote the learned data. As data points increase from 500 to 2000, these points coalesce into blue and red orbits, forming distinct embedding orbits for the reference and learned data. These closed orbits reveal the periodicity of the von Kármán vortex street. The close alignment between the learned and reference orbits indicates that the learned data successfully replicates the underlying dynamics of the ground truth at the PDF level. To investigate this concept further, we examine the first period of von Kármán vortex streets. Thirteen sparse black and green dots mark 13 states within a single period of von Kármán vortex streets. Four of these representative states are displayed using vorticity plots, obtained by reconstructing the kinetic data into macroscopic velocity fields. When the orbit is densely populated with data points, it suggests that populating the embedding space with a large number of samples enables the neural network to accurately capture next-step predictions, thereby overcoming the limitations of single-step forward modelling.

Our framework accelerates prediction from LB simulation by approximately 167 times (see Table 2). Importantly, Fig. 3b, d demonstrate that the model generalises and supports stable super resolution inference for the distribution functions even when the collision parameter and jumping timestep change that higher LB resolution. Detailed information is presented in Supplementary Information Section S6. An ablation study comparing FNO models trained with and without physics-based constraints was also conducted. The model trained with the combined MSE+Phys loss consistently outperforms the one trained with MSE alone (see Supplementary Information, Section S7.1).

**Table 2:** Comparison of simulation and prediction runtimes

Examples	Simulation time (CPU)	Prediction time (CPU)	Acceleration
Kármán vortex street LR (c++)	4.18 ± 0.5s	0.025 ± 0.0012*s*	≈ 167.2 times
Liquid ligament breakup (c++)	0.31 ± 0.03s	0.043 ± 0.0011s	≈ 7.2 times
Bubble adhesion in phase space	22.79 ± 0.74s	0.043 ± 0.0018*s*	≈ 530.08 times

All LB simulations were measured in single precision (FP32) and repeated ten times; the reported values denote the mean, with the accompanying “ ± ” indicating the standard deviation. All ML predictions were also evaluated in FP32 and repeated one hundred times, with mean and standard deviation reported in the same format. The precise definition of the acceleration metric is provided in Supplementary Information S3.

### Liquid cylinder breakup

**Fig. 4:**
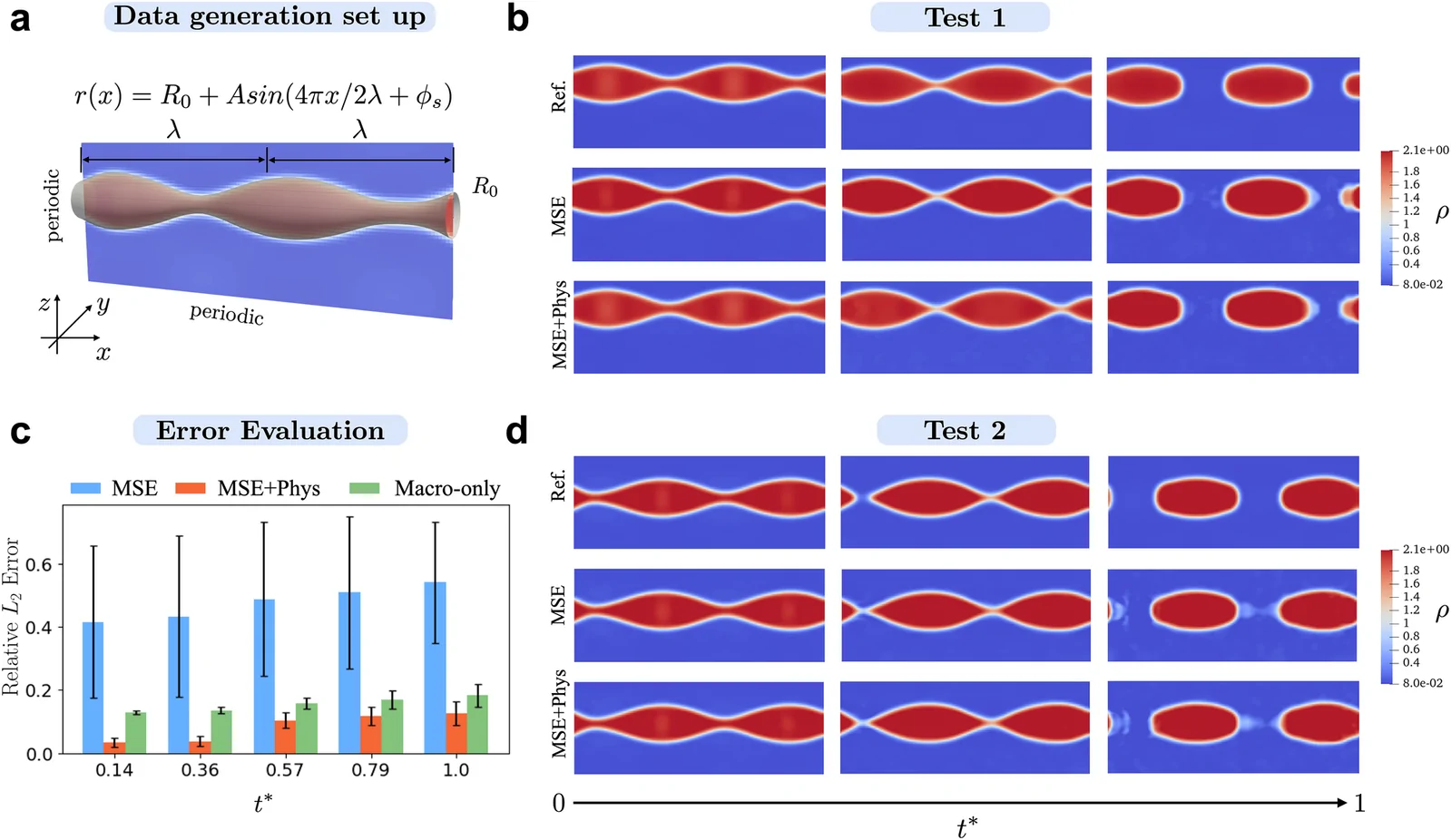
Study of liquid ligament breakup. The test case is selected from a random initial configuration in the test dataset. Panel **a** illustrates the simulation setup for the dataset, showing the ligament radius *R*_0_ and phase shift *ϕ*_*s*_. The wavelength of the simulation is denoted as *λ*. Panel **b** presents test case 1 results. The top row displays the ground truth of the ligament breakup process, the middle row shows the predicted breakup process learned solely using the MSE constraint, and the bottom row presents the prediction using MSE+Phys constraint. Panel **c** shows the error evaluation *L*_2_ as a function of t^*^, where *L*_2_ is measured based on the reconstructed density from the predicted distribution functions. The Macro-only model performs competitively, whereas the standard MSE model shows large error and variance. The MSE+Phys model achieves the lowest error and greatly improved stability, demonstrating the benefit of incorporating the physics-based constraint into the PDF evolution. Bars correspond to MSE (blue), MSE+Phys (orange), and Macro-only (green) models; error bars represent 95% confidence intervals across *n* = 10 independently trained models. **d** Presents test case 2; the top, middle, and bottom rows display the ground truth, the prediction using only the MSE loss, and the prediction using MSE+Phys loss, respectively.

This example illustrates the capability of our learning framework to describe multi-phase problems. The cylinder ligament is set up within the 3-dimensional domain with periodic boundary conditions in the *x*, *y* and *z* directions. The ligament surface is modelled via $r(x)={R}_{0}+A\sin (4\pi x/2\Lambda+{\phi }_{s})$, with *r*(*x*) as the local radius of the cylinder, *Λ* is the wavelength, *ϕ*_*s*_ is the phase shift, *A* represents the amplitude of the sin function (Fig. 4a). We set *A* = 2.5 LU, while the centre of the ligament is located at *x*_*c*_. The dataset is generated by varying *x*_*c*_ ∈ [14: 2: 34] and *ϕ*_*s*_ ∈ [0.04: 0.05: 2), see in Table 1 the second row. For training purposes, we take the *xz* plane to be located in the middle cross section of the *y* direction to focus on the region of interest. The prediction time interval is set to *Δt*_*n*_ = 10*Δt* to capture the rapid transitions occurring during ligament breakup. We divide the dataset into a split of 80% training, 10% validation, and 10% testing for the training and evaluation process. The detailed data generation parameters for the simulations in Eq. (7) can be found in Supplementary Information Section S5.

Figure 4b demonstrates a ligament breakup test case with dimensionless number t^*^ = *t*/*t*_end_ starting from ${t}^{*}\in \left[0,1\right]$. Both the MSE constraint (middle row) and the MSE+Phys constraint (bottom row) are able to capture the breakup process. The MSE+Phys constraint prediction shows less spur currency near the breakup regime. In Fig. 4d, we further examine test case 2, which is also an unseen scenario in the test dataset, and we observe a similar situation for the MSE+Phys constraint, which is marginally better than solely using the MSE constraint. Although the MSE+Phys constraint initially appears to be only slightly more effective than the MSE constraint alone, a more detailed comparison reveals a significant advantage. To rigorously assess this, we conducted a study of 10 independent training models for the constraints of MSE and MSE+Phys (see Supplementary Information Section S6). Our analysis shows that the MSE constraint tends to converge prematurely with a high *L*_2_ error, often getting stuck in local minima, which leads to prediction failures. In contrast, the MSE+Phys constraint consistently enhances the training success rate, yielding more robust and reliable outcomes. Further testing on a set of 10 pre-trained models corroborates these findings. Figure 4c shows the relative *L*_2_ error of macroscopic predictions evaluated at five normalised time points t^*^ = 0.14, 0.36, 0.57, 0.79, 1.0. Error bars represent 95% confidence intervals across 10 independently trained models. The MSE model exhibits the largest error and variance, reflecting the sensitivity of the PDF fields during the interface-breakup process. The macroscopic-only model achieves competitive accuracy, which is expected since density is the primary state variable governing dynamics in this system. In contrast, the MSE+Phys model consistently produces the lowest error and the smallest variance across the full prediction horizon. This indicates that the physics-based constraint stabilises the PDF evolution and allows the model to exploit the richer single-cell information contained in the PDF representation. This result highlights the improved stability and predictive accuracy achieved by incorporating the physics-informed loss during training. The ablation study for FNO follows similar trends for both constraints (See Supplementary Information section S7.2). Additionally, the model achieves ~7.2 times speedup relative to the corresponding LB simulation (See Table 2). The lower acceleration observed in the ligament-breakup case arises from the smaller temporal jump used (*Δt* = 10).

### Bubble adhesion

**Fig. 5:**
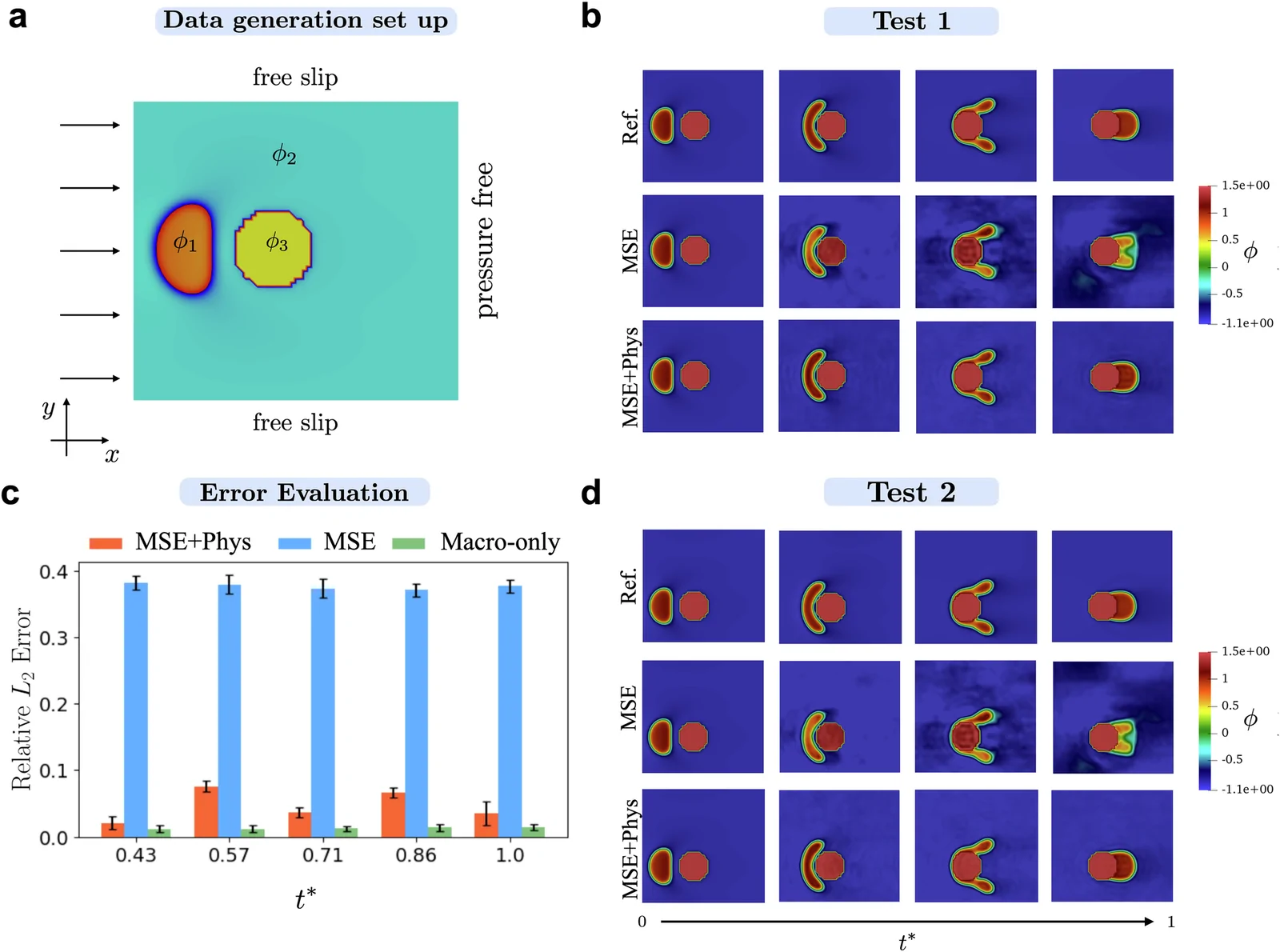
Binary phase capture by a cylinder in 2D. Panel **a** presents a sketch of the simulation setup, showing three phases: fluid 1, *ϕ*_1_, in orange, fluid 2, *ϕ*_2_, in green, and the solid cylinder, *ϕ*_3_, in yellow. **b** Depicts the evolution of a bubble over a sphere with radius *R*_*s*_ = 4 LU. The top row shows the ground truth phase field evolution, the middle row presents the phase field prediction using the MSE constraint, and the bottom row illustrates the prediction using MSE+Phys constraints, as described in Eq.(3). Panel **c** shows the error evaluation *L*_2_ as a function of dimensionless t^*^, where *L*_2_ is measured based on the reconstructed phase from the predicted distribution functions. As a result, direct Macro-only prediction achieves lower error, while MSE+Phys remains competitive. Bars correspond to MSE (blue), MSE+Phys (orange), and Macro-only (green) models; error bars represent 95% confidence intervals across *n* = 10 independently trained models. Panel **d** presents test case 2 over time, with the top, middle, and bottom rows displaying the ground truth, the prediction using the MSE loss, and the prediction using the MSE+Phys loss, respectively.

We demonstrate our learning framework applied to the phase-field LB model coupled with the hydrodynamic LB model for simulating bubble adhesion in the presence of a cylinder. In this example, we learn the phase-field LB model in 2D, which equivalently matches the Cahn-Hilliard equations^84^ (see the Supplementary Information Section S2.). Figure 5a illustrates the general simulation setup. The simulation data generation is conducted within a 2D domain of size *L*_*x*_ × *L*_*y*_, measuring E84 × 78 LU. This domain comprises three phases: *ϕ*_1_, *ϕ*_2_, and *ϕ*_3_, representing the bubble, fluid field, and solid cylinder, respectively. The fluid enters the domain with an inlet velocity directed as shown by the arrows, while the top and bottom boundaries are defined by free-slip boundary conditions.

The outlet boundary is set as a pressure-free condition. The cylinder’s centre coordinates, *x*_*d*_ and *y*_*d*_, are uniformly distributed across the domain, resulting in a total dataset size of *N*_total_ = 496, as indicated in the third row of Table 1. Each prediction time interval is set to *Δt*_*n*_ = 300*Δt*. We split the dataset into 80% training, 10% validation, and 10% testing. Specifically, we used *N*_train_ = 396 samples for training, *N*_val_ = 50 for validation, and *N*_test_ = 50 for testing. The training and validation sets are used to optimise model performance, while the test set includes previously unseen configurations. Detailed data generation parameters related to the simulations governed by Eq. (7) are provided in Supplementary Information Section S5.

Figure 5b, d shows two test cases for bubble adhesion at different locations. The bubble crosses over the cylinder, splits into two sub-bubbles and then merges again. The results show that the MSE+Phys constraint is able to capture the complete dynamics, whereas the MSE constraint can only capture the dynamics up to the splitting process, failing to merge the bubble afterwards. In general, the MSE+Phys constraint case outperforms the MSE-only case. Next, we conducted 10 independent training runs for both the MSE and MSE+Phys constraint configurations (see Supplementary Information Section S6 for details on the training progression and *L*_2_ convergence). A comparative evaluation of the relative error is shown in Fig. 5c.Under the same architecture, dataset, and training conditions, the standard PDF-level MSE model exhibits the largest error and variance throughout the prediction horizon. The macroscopic-only model achieves the lowest error for the phase-field variable, which is expected because phase is the primary scalar quantity evolved in this D2Q9 phase-field system. The MSE+Phys model, however, provides a clear improvement over the MSE-only formulation, yielding significantly lower error and markedly reduced variance across all time instances. Error bars indicate the 95% confidence interval across the ensemble of *n* = 10 independently trained models. These results demonstrate that the physics-based constraint stabilises the PDF evolution, even though direct macroscopic prediction remains highly effective for this scalar phase-field variable. Importantly, although macroscopic prediction is highly effective for this scalar field, predicting a single macroscopic variable cannot reconstruct the full set of PDFs, and therefore cannot serve as a surrogate for accelerating the underlying LBE solver. This physics-based constraint thus remains essential when the goal is to learn the forward PDF evolution. Moreover, our approach provides a  ≈ 530 × acceleration over the LB solver (see Table 2).

Finally, we conduct an ablation study on FNO for the bubble adhesion study, which reveals consistent trends across both types of constraints (see Supplementary Information, Section S7.3). This example showcases the versatility of our PDF-level data-driven PINO method. Our approach can also learn phase-field-based LB models^84^, demonstrating the generality of our framework.

## Discussion

This paper presents a physics-informed neural operator, implemented using the U-shaped neural operator architecture, to learn the fast-forward dynamics of the lattice Boltzmann equation. UNO is chosen for its memory efficiency, making it well-suited for large-scale LB modelling. Rather than relying on explicit collision kernel modelling or step-by-step integration, our approach learns the long-range evolution of the distribution functions, effectively bypassing the limitations imposed by the collision operator and enabling fast-forward prediction over extended time intervals. To ensure physical fidelity, we design a composite loss function that integrates mean-squared error with local moment-matching and global equivariance constraints. The moment-matching terms promote accurate meso-to-macroscale consistency by preserving quantities such as density and momentum, while the equivariance constraint regularises symmetry under global transformations. Crucially, this global equivariance is induced by the underlying local lattice structure, ensuring that the learned dynamics remain consistent with the discrete symmetries inherent in the LBE formulation. We evaluate the proposed framework on three representative problems: von Kármán vortex streets, liquid cylinder breakup and bubble adhesion. These examples span a wide range of kinetic regimes, including vortex shedding, multiphase interfacial dynamics and phase-field modelling. By predicting the evolution of single-particle distribution functions, our model reconstructs macroscopic quantities such as velocity, density and phase fields without requiring explicit macroscopic closures or predefined lattice rules. This physics-informed neural operator framework also offers extensibility to other physical observables, such as flux tensors, through higher-order moment-matching loss. Ablation studies replacing the U-shaped neural operator with the Fourier neural operator confirm that the proposed physics-informed constraints yield consistent performance improvements across architectures.

Beyond these neural operators, our framework is readily extendable to other neural operator families, offering a promising avenue for future development. The kinetic representation naturally requires more memory than macroscopic fields (19 versus 4 variables per cell in D3Q19 and 9 versus 3 in D2Q9), which limits the feasible training domain size. Nevertheless, across the benchmark cases, our comparison shows that PDF-level learning provides clear benefits in D3Q19 systems, where the richer PDF representation captures internal correlations that improve macroscopic accuracy, while yielding comparable performance to macroscopic only models in the D2Q9 phase field case. Despite the higher memory cost, directly learning the PDF evolution remains a reliable and physically consistent route for accelerating lattice Boltzmann simulations, as only the kinetic formulation preserves the non equilibrium information that cannot be recovered from macroscopic simulations variables when higher order moment information is missing. Learning macroscopic closures such as stresses, effective viscosities, or phase-field fluxes is a complementary strategy for hybrid solvers, but it does not replace the LB state: each subsequent LB update would still require reconstructing the full distribution functions, with the associated reconstruction error compounding over a rollout. The present work, therefore, advances the distribution functions directly, from which macroscopic and closure-related quantities can be recovered by moment operations. Using LB-derived data to train macroscopic closure or correction models for hydrodynamic solvers remains a valuable, distinct learning problem. Additionally, our framework achieves acceleration over conventional lattice Boltzmann solvers by between two and three orders of magnitude. The ability to capture complex, coupled physical dynamics, including small-scale oscillations and long-range transport, using fast-forward evolution of the distribution functions makes our framework a promising tool for data-driven modelling of physical systems governed by partial differential equations, while preserving their underlying distribution function’s structure. Although the examples considered here lie within regimes where continuum descriptions are valid, our contribution is to provide an accelerated, LBE-level surrogate that remains agnostic to the specific collision operator. This allows the learned PDF update to be reused across a broad class of lattice Boltzmann applications without requiring model-specific reformulation.

Looking forward, while we have successfully applied the neural-operator architecture to all the LBE problems discussed in this work, extending the framework to a wider variety of geometries and flow configurations will require additional data preparation and larger curated datasets. Building toward such a broader generalisation represents an important future direction, aligned with our longer-term goal of developing a generalisable neural operator for lattice Boltzmann methods that can transfer across diverse kinetic scenarios. The break-even analysis in the Supplementary Information also clarifies the present limitation of the work: the current models are trained per case on relatively narrow geometry and parameter bands, so their reuse window is bounded by those training distributions. The reported amortisation should therefore be read as a conservative per-case estimate. A more general LB neural operator trained across geometries, lattice models, collision kernels, and flow regimes would spread the same offline data-generation and training investment over far more downstream simulations and inference queries. The present approach is also complementary to implicit or semi-implicit kinetic solvers: those methods change the time integrator, whereas our model learns a reusable large-step forward map for the distribution functions generated by the standard stream–collide workflow. Another promising route is to use ML-based operators as accelerated temporal jump solvers within an LB solver framework, where the ML model advances the solution over large time steps while the LB solver provides fine-scale corrections.

## Methods

### Physics-Informed training of neural operators for the lattice Boltzmann equation

We approximate the global operator ${\mathcal{G}}$ introduced in Section II using neural operator architectures. We employ either the U-shaped Neural Operator or the Fourier Neural Operator to parameterise the spatiotemporal mapping from *f*_in_ to ${\hat{f}}_{{\rm{out}}}$. Detailed formulations of both architectures are provided in Supplementary Information S4. To ensure that the learned operator remains faithful to the underlying kinetic physics of the LBE, we train it with the physics-informed loss described below, which augments standard data fidelity with moment-matching and equivariance constraints. To train the neural operator, we define a composite loss function that combines data fidelity with physics-informed constraints. The total loss is

3 $${\mathcal{L}}\,=\,{\lambda }_{{\rm{MSE}}}\,{{\mathcal{L}}}_{{\rm{MSE}}}\,+\,{\lambda }_{{\rm{mom}},0}\,{{\mathcal{L}}}_{{\rm{mom}},0}\,+\,{\lambda }_{{\rm{mom}},1}\,{{\mathcal{L}}}_{{\rm{mom}},1}\,+\,{\lambda }_{{\rm{equiv}}}\,{{\mathcal{L}}}_{{\rm{equiv}}},$$

where ${{\mathcal{L}}}_{{\rm{MSE}}}$ measures the discrepancy between predicted and reference distribution functions, ${{\mathcal{L}}}_{{\rm{mom}},0}$ and ${{\mathcal{L}}}_{{\rm{mom}},1}$ regularises meso-to-macroscopic of zeroth- and first-order moments (mass and momentum), and ${{\mathcal{L}}}_{{\rm{equiv}}}$ regularises moment equivalence between predicted and reference distributions. The coefficients *λ*_MSE_, *λ*_mom,0_, *λ*_mom,1_, *λ*_equiv_ are non-negative coefficients that balance the contribution of each term. ${{\mathcal{L}}}_{{\rm{equiv}}}$ penalises violation of global LBE equivariance (See section Methods C). The moment matching losses are defined by:

4 $${{\mathcal{L}}}_{{\rm{mom}},0}={\left|\left| \mathop{\sum }\limits_{i=0}^{Q-1}{\hat{f}}_{i}(\cdot,\cdot )-\mathop{\sum }\limits_{i=0}^{Q-1}{f}_{i}(\cdot,\cdot )\right|\right| }_{2}^{2},$$

5 $${{\mathcal{L}}}_{{\rm{mom}},1}={\left|\left| \mathop{\sum }\limits_{i=0}^{Q-1}{\hat{f}}_{i}(\cdot,\cdot )\,{{\bf{c}}}_{i}-\mathop{\sum }\limits_{i=0}^{Q-1}{f}_{i}(\cdot,\cdot )\,{{\bf{c}}}_{i}\right|\right| }_{2}^{2},$$

where ${\hat{f}}_{i}$ is the predicted distribution functions. These losses are computed locally across space and time to ensure the preservation of meso-to-macroscale consistency. Higher-order moments are not included, as the training data does not reliably resolve them, and they introduce sensitivity to numerical noise.

To ensure that the learned dynamics respect the global equivariance property of the lattice Boltzmann equation, we incorporate an equivariance loss that combines global spatial transformations with local permutations of the discrete velocity channels, both determined by the underlying lattice structure. Although local equivariance is commonly regularised in LBE learning frameworks^68^, it becomes unreliable over long time intervals due to the compounded effects of streaming and collision, which break local symmetry even when initial conditions are symmetric. Since our neural operator models long-time evolution by directly predicting large temporal jumps rather than simulating intermediate steps, enforcing local equivariance is inappropriate. Instead, we promote global equivariance, defined as the invariance of the full distribution field under joint spatial transformation and velocity permutation, which remains physically meaningful over extended time horizons. We therefore define a global equivariance loss:

6 $${{\mathcal{L}}}_{{\rm{equiv}}}=\sum _{{\bf{R}}\in {{\mathcal{S}}}_{h}}{\left\Vert {\mathcal{G}}({\bf{R}}\cdot f)-{\bf{R}}\cdot {\mathcal{G}}(f)\right\Vert }^{2},$$

where ${{\mathcal{S}}}_{h}$ denotes the discrete symmetry group of the lattice (e.g., dihedral group *D*_4_ in 2D), and the group action **R** ⋅ *f* applies both spatial transformations and velocity index permutations. This constraint promotes physically consistent evolution of the distribution functions under rigid transformations of the full system state. The weights *λ*_MSE_, *λ*_mom,0_, *λ*_mom,1_, and *λ*_equiv_ are treated as hyperparameters and selected based on validation performance. A detailed study of their setup is provided in Supplementary Information Section S3. Note that since each kinetic snapshot contains all discrete distribution functions across the full spatial domain (e.g., 19 variables per cell in D3Q19 and 9 in D2Q9), the memory footprint of kinetic datasets is larger than that of macroscopic-only datasets. This high dimensionality limits feasible dataset sizes and batch sizes during training.

### The lattice Boltzmann method

This work uses the LB simulation to generate the kinetic data. The detailed description of the lattice Boltzmann method is given in Supplementary Information Section S1. We use both two-dimensional and three-dimensional LB models, referred to as D2QX and D3QX, where *X* denotes the number of discrete velocities for the lattice cell. The governing equation for the distribution function *f*_*i*_(**x**, *t*), accounting for collision and forcing, can be expressed as:

7 $${f}_{i}({\bf{x}}+{{\bf{c}}}_{i}\Delta t,t+\Delta t)={f}_{i}({\bf{x}},t)+\Omega \left[{f}_{i}({\bf{x}},t)-{f}_{i}^{{\rm{eq}}}({\bf{x}},t)\right]+{F}_{i}({\bf{x}},t),$$

where *Ω* denotes the collision operator, **c**_*i*_ represents the *i*th discrete velocity vector, ${f}_{i}^{{\rm{eq}}}$ is the equilibrium distribution function, and *F*_*i*_ is the forcing term. A common choice is the Bhatnagar-Gross-Krook (BGK) collision operator, which can be written as $\Omega ({\bf{x}},t)=-\frac{1}{\tau }$. Other collision operators, for example, the multiple relaxation time (MRT) kernel^85^ and the cumulant kernel^86^, can also be used for higher Reynolds number flows. A detailed description of the collision operators employed in this work is provided in Supplementary Information Section S1.

Macroscale quantities such as density and momentum can be obtained through moment-matching as defined in Eq. (4) and Eq. (5). Higher-order moments are not considered in this work, as the LB data used does not preserve physically meaningful information beyond the first moment. The discretized expressions for the conserved macroscopic quantities are:

8 $$\rho ({\bf{x}},t)=\mathop{\sum }\limits_{i=0}^{Q-1}{f}_{i}({\bf{x}},t),$$

9 $$\rho ({\bf{x}},t){\bf{u}}({\bf{x}},t)=\mathop{\sum }\limits_{i=0}^{Q-1}{f}_{i}({\bf{x}},t){{\bf{c}}}_{i},$$

where *Q* is the number of discrete velocity directions.

### Global equivariance induced by lattice symmetries

The discrete symmetry groups *D*_4_ (for 2D lattice models) and the 24-element proper cubic rotation group *O* (for 3D lattice models) are not only essential to the physical structure of the lattice Boltzmann models but also play a central role in our neural operator framework. Specifically, these local lattice symmetries induce a global equivariance structure over the full distribution function fields. In the context of our model, global equivariance refers to the invariance of the neural operator’s output under joint spatial transformations and velocity-index permutations, both defined by the corresponding lattice symmetry group. Enforcing global equivariance in this way ensures that the predicted evolution of the system remains consistent under rigid transformations of the entire domain, making the learned dynamics physically meaningful over long time jumps.

Here, ${\bf{R}}\in {{\mathcal{S}}}_{h}$ denotes a group element acting on spatial coordinates and associated with a permutation *σ*_**R**_ on the velocity indices such that:

10 $${\bf{R}}\,{{\bf{c}}}_{{\sigma }_{{\bf{R}}}(i)}={{\bf{c}}}_{i},\quad \forall i.$$

Then, the group action on the distribution field is defined as:

11 $${[{\bf{R}}\cdot f]}_{i}({\bf{x}},t):={f}_{{\sigma }_{{\bf{R}}}(i)}({{\bf{R}}}^{-1}{\bf{x}},t).$$

We say that the neural operator ${\mathcal{G}}$ is globally equivariant if:

12 $${\mathcal{G}}({\bf{R}}\cdot f)={\bf{R}}\cdot {\mathcal{G}}(f),\quad \forall {\bf{R}}\in {{\mathcal{S}}}_{h}.$$

This relation ensures that a rigid transformation of the input field, rotating or reflecting the entire spatial domain and permuting velocity channels accordingly, results in a correspondingly transformed output field. Unlike local equivariance, which is defined at the level of individual lattice cells and may break due to streaming dynamics, global equivariance captures symmetries at the field level and remains robust over long time horizons. It is important to note that we regularise toward the discrete symmetry group induced by the lattice, rather than the full continuous rotation group. For example, the D2Q9 lattice is invariant only under the dihedral group *D*_4_ (rotations by multiples of 90^∘^ and reflections), and the D3Q19 lattice under the proper cubic rotation group *O*. Our equivariance loss therefore regularises these discrete symmetries, which are exactly those respected by the underlying lattice Boltzmann formulation. The full proof of global equivariance for the lattice Boltzmann equation is provided in Supplementary Information Section S2.5.

## Supplementary information


Supplementary information
Transparent Peer Review file


## Data Availability

The kinetic datasets generated and analysed in this study, including the lattice Boltzmann simulation snapshots used to train and evaluate the neural operator for the von Kármán vortex street, liquid ligament breakup, and bubble adhesion benchmarks, have been deposited in the Zenodo repository under the accession code (https://doi.org/10.5281/zenodo.20529005^76^). The processed datasets used for figure generation are available in the same record.

## Code availability

The source code for training and inference of the physics-informed neural operator, together with scripts to reproduce the figures and tables in this study, is available at (https://github.com/UCL-CCS/Fast-forward-lattice-Boltzmann) under the MIT licence.
