# Supplementary material for: Fast-forward prediction of lattice Boltzmann dynamics with physics-informed neural operators
Source: Nat Commun. 2026 Jul 29;17:9243. doi: 10.1038/s41467-026-75730-1 (PMC13526859; doi:10.1038/s41467-026-75730-1)
Supplement: Supplementary file 1 [file 41467_2026_75730_MOESM1_ESM.pdf]

Supplementary Information

Fast-forward prediction of lattice Boltzmann dynamics with physics-informed neural operators

Xiao Xue[1+], Marco F.P. ten Eikelder[2], Mingyang Gao[1], Xiaoyuan Cheng[3], Yiming Yang[4], Yi He[3], Sibo Cheng[6], Shuo Wang[5], Yukun Hu[2+], Peter V. Coveney[1,7,8+]

[1] Centre for Computational Science, University College London, UK

[2] Institute for Mechanics, Computational Mechanics Group, Technical University of Darmstadt, Germany

[3] Centre for Dynamic Systems, University College London, UK

[4] Department of Statistical Science, University College London, UK

[5] Department of Applied Physics, Eindhoven University of Technology, The Netherlands

[6] Centre d'Enseignement et de Recherche en Environnement Atmosphérique (CEREA), École des Ponts and EDF R&D, Institut Polytechnique de Paris, Île-de-France, France

[7] Computational Science Laboratory, Institute for Informatics, University of Amsterdam, The Netherlands

[8] Centre for Advanced Research Computing, University College London, UK

+Email: x.xue@ucl.ac.uk, yukun.hu@ucl.ac.uk, p.v.coveney@ucl.ac.uk

This Supplementary Information document provides comprehensive details to support and extend the findings presented in the main text, with a focus on the implementation and evaluation of the physics-informed neural operator framework for the lattice Boltzmann equation (LBE). Section S1 introduces the mathematical formulation of the LBE and its discretization, including various collision operators used in our simulations. Section S2 discusses lattice model symmetry considerations, covering both 2D and 3D discrete velocity sets and their group-theoretic properties. The architecture of the neural operator, including our implementation of U-shaped Neural Operator (UNO) and Fourier Neural Operator (FNO) backbones, is detailed in Section S3. Sections S4 and S5 describe the data generation process and experimental setups used across the benchmark problems: von Kármán vortex street, liquid ligament breakup, and bubble adhesion. Additional results and visualizations are provided in Section S6 to further illustrate model performance. S7 demonstrates the ablation study for FNO on proposed examples. Finally, Section S8 defines the error metrics and presents an ablation study, including comparisons between UNO and FNO, and assessments of different loss components.

CONTENTS

S1. Lattice Boltzmann equations and collision kernels 4

S1.1 The lattice Boltzmann method 4

S1.2 Bhatnagar–Gross–Krook collision operator and equilibrium 4

S1.3 Multiple relaxation time collision operator 5

S1.4 Smagorinsky subgrid-scale modeling 5

S1.5 Shan-Chen interaction model 5

S1.6 Phase-field lattice Boltzmann method 6

S1.7 Unit conversion between lattice units and physical units 7

S2. Lattice model and symmetry 8

S2.1 D2Q9 lattice cell model 8

S2.2 Dihedral symmetry group for 2D lattice cell 8

S2.3 D3Q19 lattice cell model 8

S2.4 Proper octahedral rotation group for 3D lattice cell 9

S2.5 Equivariance of the Lattice Boltzmann equation 9

S3. Representative Applications and Performance Analysis of the PINO Framework 11

S4. Network architecture details & training 14

S4.1 UNO architecture details 15

S4.2 FNO architecture details 17

S4.3 Traning proceedure 19

S5. Data generation 21

S5.1 Von Kármán vortex street set up 21

S5.2 Cylindrical ligament breakup set up 22

S5.3 Bubble adhesion set up 22

S6. Additional results 23

S6.1 Von Kármán vortex street: low resolution vs super resolution 23

1. Low resolution prediction 24

2. Super resolution prediction 25

S6.2 Cylindrical ligament breakup: error comparison 27

S6.3 Bubble adhesion: error comparison 27

S6.4 Training evolution comparison for selected applications 28

S7. Ablation study: FNO results 34

S7.1 Von Kármán vortex street 35

S7.2 Liquid ligament breakup 35

S7.3 Bubble adhesion 35

S8. Error metrics 39

Supplementary References 40

S1. LATTICE BOLTZMANN EQUATIONS AND COLLISION KERNELS

In this section, we first discretise the Boltzmann equation to the lattice Boltzmann (LB) formulation. Then, we review the LB equations, focusing on both the hydrodynamic and phase-field limits. In addition, we integrate various collision kernels employed in data generation. We begin by recalling the general LB equation.

S1.1 The lattice Boltzmann method

The governing equation for the single-particle distribution functions $f_i(\mathbf{x}, t)$, located at position $\mathbf{x}$ and time t, accounting for collisions and external forcing, is

$$f_i(\mathbf{x} + \mathbf{c}_i\Delta t,\ t + \Delta t) = f_i(\mathbf{x}, t) + \Omega_i(\mathbf{x}, t) + F_i(\mathbf{x}, t), \tag{S1}$$

where $\mathbf{c}_i$ is the ith discrete velocity, $\Omega_i(\mathbf{x}, t)$ is the collision term, and $F_i(\mathbf{x}, t)$ is the forcing term. The macroscopic fields are recovered via the standard moments

$$\rho(\mathbf{x}, t) = \sum_{i=0}^{Q-1} f_i(\mathbf{x}, t), \qquad \rho(\mathbf{x}, t)\, \mathbf{u}(\mathbf{x}, t) = \sum_{i=0}^{Q-1} f_i(\mathbf{x}, t)\, \mathbf{c}_i. \tag{S2}$$

S1.2 Bhatnagar–Gross–Krook collision operator and equilibrium

In the single-relaxation-time (BGK) model, each component relaxes toward its local equilibrium $f_i^{\mathrm{eq}}(\rho, \mathbf{u})$:

$$\Omega_i(\mathbf{x}, t) \ = \ -\frac{1}{\tau}\left(f_i(\mathbf{x}, t) - f_i^{\mathrm{eq}}(\rho(\mathbf{x}, t), \mathbf{u}(\mathbf{x}, t))\right), \tag{S3}$$

where the relaxation time τ relates to the kinematic viscosity ν by

$$\nu \ = \ c_s^2\left(\tau - \tfrac{1}{2}\right)\Delta t, \tag{S4}$$

with c_s the lattice speed of sound (for standard lattices, $c_s^2 = 1/3$) and Δt the timestep (set to unity in our simulations). The second-order equilibrium distribution used in this work is

$$f_i^{\mathrm{eq}}(\rho, \mathbf{u}) \ = \ w_i\, \rho \left[1 + \frac{\mathbf{c}_i \cdot \mathbf{u}}{c_s^2} + \frac{(\mathbf{c}_i \cdot \mathbf{u})^2}{2c_s^4} - \frac{\mathbf{u} \cdot \mathbf{u}}{2c_s^2}\right], \tag{S5}$$

where $\{\mathbf{c}_i\}_{i=0}^{Q-1}$ denotes the set of discrete lattice velocities (e.g., for D2Q9 or D3Q19), $\{w_i\}$ are the corresponding lattice weights, c_s is the lattice speed of sound (for standard lattices $c_s^2 = 1/3$), and $\cdot$ denotes the Euclidean inner product. For reference, the standard choices are

$$\text{D2Q9: } w_0 = \tfrac{4}{9},\ w_{1\text{–}4} = \tfrac{1}{9},\ w_{5\text{–}8} = \tfrac{1}{36}; \qquad \text{D3Q19: } w_0 = \tfrac{1}{3},\ w_{1\text{–}6} = \tfrac{1}{18},\ w_{7\text{–}18} = \tfrac{1}{36}.$$

S1.3 Multiple relaxation time collision operator

As described in Eq. S1, the collision term can be generalized by relaxing different kinetic moments at different rates, which leads to the multiple relaxation time (MRT) collision operator [1]:

$$\Omega_i(\mathbf{x}, t) = -\Big[\mathbf{M}^{-1}\,\boldsymbol{\Lambda}\,\mathbf{M}\left(\mathbf{f} - \mathbf{f}^{\mathrm{eq}}\right)\Big]_i, \tag{S6}$$

where the distribution vector is

$$\mathbf{f} = \big(f_0(\mathbf{x}, t), f_1(\mathbf{x}, t), \ldots, f_{Q-1}(\mathbf{x}, t)\big)^\top,$$

and likewise for $\mathbf{f}^{\mathrm{eq}}$. Here $\mathbf{M}$ is the moment-transformation matrix and $\boldsymbol{\Lambda}$ is a diagonal matrix of relaxation rates in moment space. The explicit form of $\mathbf{M}$ used in this work is given in Eq. S37.

S1.4 Smagorinsky subgrid-scale modeling

In this section, we provide a brief description of the Smagorinsky subgrid-scale (SGS) large-eddy simulation (LES) model within the lattice Boltzmann (LB) framework. The effective viscosity ν_{eff} [2–4] is modeled as the sum of the molecular viscosity ν (given in Eq. S4) and the turbulent viscosity ν_t:

$$\nu_{\mathrm{eff}} = \nu + \nu_t, \qquad \nu_t = C_{\mathrm{smag}}\,\Delta^2\,\|\bar{\mathbf{S}}\|, \tag{S7}$$

where $\bar{\mathbf{S}}$ is the filtered strain-rate tensor, $\|\bar{\mathbf{S}}\| = (2S_{\alpha\beta}S_{\alpha\beta})^{1/2}$ denotes its Frobenius norm, C_{smag} is the Smagorinsky constant (set to 0.01 in this work), and Δ is the filter width.

In the LB formulation, ν_{eff} enters through the relaxation time τ_{eff} in Eq. S4, replacing ν by ν_{eff}:

$$\nu_{\mathrm{eff}} = c_s^2\left(\tau_{\mathrm{eff}} - \tfrac{1}{2}\right)\Delta t.$$

This modification accounts for the effect of unresolved turbulent stresses within the collision operator. A detailed implementation of LB-based SGS modeling can be found in Xue et al. [5].

S1.5 Shan-Chen interaction model

In the contraction of the liquid ligament configuration outlined in Table S1, we apply a forcing model to account for non-ideal multiphase/multicomponent interactions using

the Shan–Chen formulation for mixtures [6–10]. The force exerted on fluid phase l due to the surrounding fluid component l' is expressed as

$$F_l(\mathbf{x},t) = -\,\mathcal{G}_{sc}\,\rho_l(\mathbf{x},t)\sum_{l'\neq l}\sum_{i=0}^{Q-1}\omega_i\,\rho_{l'}(\mathbf{x}+\mathbf{c}_i\Delta t,t)\,\mathbf{c}_i, \tag{S8}$$

where $\rho_l(\mathbf{x},t)$ denotes the density of fluid component/phase l, $\mathcal{G}_{sc}$ is a coupling coefficient regulating the interaction strength between fluid components, ω_i are the lattice weights, and $\mathbf{c}_i$ are the discrete lattice velocities.

S1.6 Phase-field lattice Boltzmann method

For the bubble adhesion example, we employ a phase-field LB model coupled with the standard hydrodynamic LB model (Eq. S1). The phase field $\phi(\mathbf{x},t)$ is an order parameter that distinguishes between fluid components. Its evolution is governed at the macroscopic level by the Cahn–Hilliard equation,

$$\partial_t\phi + \nabla\cdot(\phi\mathbf{u}) = \nabla\cdot(\mathcal{M}\nabla\mu), \tag{S9}$$

where $\mathbf{u}$ is the fluid velocity, μ is the chemical potential, and $\mathcal{M}$ is the mobility parameter controlling diffusive relaxation.

Within the LB framework, this equation is recovered from the dynamics of an auxiliary distribution function $g_i(\mathbf{x},t)$. The phase field is obtained as the zeroth moment of g_i:

$$\phi(\mathbf{x},t) = \sum_{i=0}^{Q-1} g_i(\mathbf{x},t). \tag{S10}$$

The evolution of g_i follows a BGK-type lattice Boltzmann equation in phase space:

$$g_i(\mathbf{x}+\mathbf{c}_i\Delta t,\, t+\Delta t) = g_i(\mathbf{x},t) - \frac{1}{\tau_\phi}\Big(g_i(\mathbf{x},t) - g_i^{\mathrm{eq}}(\mathbf{x},t)\Big), \tag{S11}$$

where τ_ϕ is the relaxation time in phase space, related to the mobility $\mathcal{M}$ by

$$\mathcal{M} = c_s^2\left(\tau_\phi - \tfrac{1}{2}\right)\Delta t. \tag{S12}$$

The equilibrium distribution g_i^{eq} is constructed such that the Cahn–Hilliard equation (S9) is recovered in the macroscopic limit. A common choice is

$$g_i^{\mathrm{eq}} = w_i\left(\frac{\Gamma\mu}{c_s^2} + \frac{\phi\,\mathbf{u}\cdot\mathbf{c}_i}{c_s^2} + \frac{\phi\big[(\mathbf{u}\cdot\mathbf{c}_i)^2 - c_s^2|\mathbf{u}|^2\big]}{2c_s^4}\right), \quad i\neq 0, \tag{S13}$$

and

$$g_0^{\mathrm{eq}} = \phi - \sum_{i\neq 0} g_i^{\mathrm{eq}}. \tag{S14}$$

Here Γ is a tunable parameter controlling the diffusive interface thickness. The chemical potential μ is obtained from the free energy functional and encodes interface forces between the two fluid phases.

The coupling between the phase field and hydrodynamic LB model arises because ϕ appears in the pressure tensor and in the chemical potential, thereby affecting the momentum equation. This formulation is commonly referred to as a phase-field LB method, since the order parameter ϕ evolves according to the Cahn–Hilliard phase-field model.

S1.7 Unit conversion between lattice units and physical units

In this subsection, we demonstrate the unit conversion between lattice units (LU) and physical units in both velocity and distance. Within the LB simulation, we can obtain the LB velocity $\mathbf{u}_{\mathrm{LB}}(\mathbf{x}, t)$ at location $\mathbf{x}$, the physical unit of the velocity, $\mathbf{u}_{\mathrm{phys}}(\mathbf{x}, t)$, can be written as

$$\mathbf{u}_{\mathrm{phys}}(\mathbf{x}, t) = \mathbf{u}_{\mathrm{LB}}(\mathbf{x}, t)\frac{c_x}{c_t}, \tag{S15}$$

where c_x and c_t are the conversion factors from the lattice Boltzmann simulation to the physical system which is defined as

$$c_x = \frac{L_{\mathrm{phys}}}{L_{\mathrm{LB}}}, \qquad c_t = \frac{t_{\mathrm{phys}}}{t_{\mathrm{LB}}}, \tag{S16}$$

where L_{phys} and t_{phys} represent the space and time units from the physical system, while L_{LB} and t_{LB} are the space and time lattice Boltzmann units from the lattice Boltzmann simulation. Accordingly, we can also obtained the physical distance y_{phys} from the LB length scale:

$$y_{\mathrm{phys}} = y_{\mathrm{LB}} c_x. \tag{S17}$$

For example, in Eq. (S16), L_{phys} may denote the height of the channel as $L_{\mathrm{phys}} = 2$m so that $L_{\mathrm{LB}} = 20$ LU. Then, $c_x = 0.1$m. Similar calculations can be performed to determine the time scale conversion factor c_t.

S2. LATTICE MODEL AND SYMMETRY

In this section, we present the lattice model for the generation of kinetic data. Specifically, we will present the D2Q9 model for the bubble adhesion case, the D3Q19 case for Kármán vortex street and the liquid ligament breakup case. In addition, the symmetry group for 2D and 3D lattice models is demonstrated in this section as well.

S2.1 D2Q9 lattice cell model

The discretised velocity set $\mathbf{c}_i$ for D2Q9 model in Eq. S11 where $i \in \{0, 1, \dots, Q-1\}$ with $Q = 9$

$$\begin{aligned} \mathbf{c}_i = \{&(0,0),(1,0),(0,1),(-1,0),(0,-1),\\ &(1,1),(-1,1),(-1,-1),(1,-1)\}. \end{aligned} \tag{S18}$$

S2.2 Dihedral symmetry group for 2D lattice cell

We define the Dihedral group D_4 for the 2D square lattice cell that consist of

$$D_4 = \{I, r, r^2, r^3, s, sr, sr^2, sr^3\} \tag{S19}$$

where I indicating the identity matrix, the r indicating 90° rotational matrix, and s indicating the reflection matrix. Here we only write out the basic element of the rotational matrix, r

$$r(\mathbf{c}_i) = \begin{pmatrix} 0 & -1 \\ 1 & 0 \end{pmatrix} \mathbf{c}_i. \tag{S20}$$

The reflection matrix can be written as:

$$s(\mathbf{c}_i) = \begin{pmatrix} 1 & 0 \\ 0 & -1 \end{pmatrix} \mathbf{c}_i \tag{S21}$$

S2.3 D3Q19 lattice cell model

The discretised velocity set $\mathbf{c}_i$ for D3Q19 model in Eq. (S1) with where $i \in \{0, 1, \dots, Q-1\}$ with $Q = 19$

$$\begin{aligned} \mathbf{c}_i = \{&(0,-1,-1),(-1,0,-1),(0,0,-1),(1,0,-1),(0,1,-1),(-1,-1,0),\\ &(0,-1,0),(1,-1,0),(-1,0,0),(0,0,0),(1,0,0),(-1,1,0),(0,1,0),\\ &(1,1,0),(0,-1,1),(-1,0,1),(0,0,1),(1,0,1),(0,1,1)\}. \end{aligned} \tag{S22}$$

S2.4 Proper octahedral rotation group for 3D lattice cell

We use the proper octahedral rotation group O for the 3D cubic lattice cell, consisting of the 24 orientation-preserving cubic rotations. This is the rotation subgroup of the full octahedral group O_h; the latter also includes improper transformations such as reflections and inversion and has 48 elements, which are not used in our 3D equivariance regularisation.

$$O = \begin{cases} I, \\ r_x(90^\circ), r_x(180^\circ), r_x(270^\circ), \\ r_y(90^\circ), r_y(180^\circ), r_y(270^\circ), \\ r_z(90^\circ), r_z(180^\circ), r_z(270^\circ), \\ r_{\mathrm{e}_1}, r_{\mathrm{e}_2}, r_{\mathrm{e}_3}, r_{\mathrm{e}_4}, r_{\mathrm{e}_5}, r_{\mathrm{e}_6}, \\ r_{\mathrm{v}_1}(120^\circ), r_{\mathrm{v}_2}(120^\circ), r_{\mathrm{v}_3}(120^\circ), r_{\mathrm{v}_4}(120^\circ), \\ r_{\mathrm{v}_1}(240^\circ), r_{\mathrm{v}_2}(240^\circ), r_{\mathrm{v}_3}(240^\circ), r_{\mathrm{v}_4}(240^\circ) \end{cases} \tag{S23}$$

where the rotation matrices have the following meaning. First, I denotes the identity rotation, representing no rotation. The rotations $r_x(\theta)$, $r_y(\theta)$, and $r_z(\theta)$ correspond to rotations by angles $\theta \in \{90^\circ, 180^\circ, 270^\circ\}$ about the x-, y-, and z-axes, respectively, capturing the primary cubic symmetries along the Cartesian directions. Additionally, the set includes r_{e_i}, which are 180° rotations about axes that pass through the midpoints of opposite edges of the cube. Finally, the elements $r_{\mathrm{v}_i}(120^\circ)$ and $r_{\mathrm{v}_i}(240^\circ)$ represent 120° and 240° rotations around axes passing through pairs of opposite vertices of the cube (or equivalently, the octahedron inscribed in it).

S2.5 Equivariance of the Lattice Boltzmann equation

A central structural aspect of the LBE is the selection of the discrete velocity set $\{\mathbf{c}_i\}$, which is invariant under a discrete subgroup $\mathcal{S}_h \subseteq O(d)$ that reflects the symmetries of the spatial discretization. In two dimensions, the common choice is the nine-velocity (D2Q9) model, where $\{\mathbf{c}_i\}$ is invariant under the group D_4, the group of symmetries of the square. Precisely, $D_4 \subset O(2)$ contains 8 matrices: 4 rotations by angles $0, \pi/2, \pi, 3\pi/2$, and 4 reflections across the axes and diagonals. For the D3Q19 lattice, we use the 24-element proper octahedral rotation group O introduced above. In general, we assume that the velocity set is invariant under symmetry group $\mathcal{S}_h$: for every $\mathbf{R} \in \mathcal{S}_h$, there exists a

permutation σ_R of the index set $\{i\}$ such that:

$$\mathbf{R}\mathbf{c}_{\sigma_R(i)} = \mathbf{c}_i, \quad \text{for all } i. \tag{S24}$$

Next, we define the application of the group $\mathcal{S}_h$ on the distribution fields through a transformation of its spatial argument:

$$[\mathbf{R}f]_i(\mathbf{x},t) = f_{\sigma_\mathbf{R}(i)}(\mathbf{R}^{-1}\mathbf{x},t), \qquad \mathbf{R} \in \mathcal{S}_h. \tag{S25}$$

The LBE is said to be equivariant under $\mathcal{S}_h$ if its structure is preserved under this group action. That is, if $f = \{f_i\}$ satisfies the LBE, then the transformed field $\mathbf{R}\cdot f$ also satisfies the same equation for every $\mathbf{R} \in \mathcal{S}_h$.

Next, we study the transformation of the LBE under $\mathbf{R} \in \mathcal{S}_h$. Applying the chain rule to the transformed streaming contribution eventually provides:

$$\partial_t[\mathbf{R}\cdot f]_i + \mathbf{c}_i \cdot \nabla[\mathbf{R}\cdot f]_i = \left(\partial_t f_{\sigma_\mathbf{R}(i)} + \mathbf{c}_{\sigma_\mathbf{R}(i)} \cdot \nabla f_{\sigma_\mathbf{R}(i)}\right)(\mathbf{R}^{-1}\mathbf{x},t). \tag{S26}$$

Hence, the streaming part is equivariant under $\mathcal{S}_h$ if the velocity set $\{\mathbf{c}_i\}$ is invariant under $\mathcal{S}_h$. Next, we focus on the collision term $Q_i(f)$ and consider the BGK approximation:

$$Q_i(f) = -\frac{1}{\tau}\left(f_i - f_i^{\text{eq}}\right), \tag{S27}$$

where $f_i^{\text{eq}} = f_i^{\text{eq}}(\rho,\mathbf{u})$ is the equilibrium distribution depending on the macroscopic density and velocity. Straightforward computations reveal the transformation of the density and the momentum. Applying $\mathbf{R} \in \mathcal{S}_h$ and using the change of variables $\tilde{\mathbf{x}} = \mathbf{R}^{-1}\mathbf{x}$, we have:

$$\rho^\mathbf{R}(\mathbf{x}) := \sum_i [\mathbf{R}\cdot f]_i(\mathbf{x}) = \sum_i f_{\sigma_\mathbf{R}(i)}(\mathbf{R}^{-1}\mathbf{x}) = \sum_j f_j(\mathbf{R}^{-1}\mathbf{x}) = \rho(\mathbf{R}^{-1}\mathbf{x}) \tag{S28a}$$

$$[\rho\mathbf{u}]^\mathbf{R}(\mathbf{x}) := \sum_i \mathbf{c}_i[\mathbf{R}\cdot f]_i(\mathbf{x}) = \sum_i \mathbf{c}_i f_{\sigma_\mathbf{R}(i)}(\mathbf{R}^{-1}\mathbf{x}) = \sum_j \mathbf{R}\mathbf{c}_j f_j(\mathbf{R}^{-1}\mathbf{x}) = \mathbf{R}\cdot(\rho\mathbf{u})(\mathbf{R}^{-1}\mathbf{x}), \tag{S28b}$$

and hence the velocity transforms as a vector field: $\mathbf{u}^\mathbf{R}(\mathbf{x}) = \mathbf{R}\cdot\mathbf{u}(\mathbf{R}^{-1}\mathbf{x})$. Therefore, since $f_i^{\text{eq}}(\rho,\mathbf{u})$ is constructed to respect rotational invariance, the transformed equilibrium distribution satisfies:

$$[\mathbf{R}\cdot f^{\text{eq}}]_i(\mathbf{x}) = f^{\text{eq}}_{\sigma_\mathbf{R}(i)}(\mathbf{R}^{-1}\mathbf{x}),$$

i.e., the same transformation law as f_i. Consequently, the collision operator is equivariant [11]:

$$[\mathbf{R}\cdot Q(f)]_i = Q_{\sigma_\mathbf{R}(i)}(f)(\mathbf{R}^{-1}\mathbf{x}) = Q_i([\mathbf{R}\cdot f])(\mathbf{x}).$$

For the other collision and forcing terms used in this work, the same equivariance principle applies provided that the lattice weights, moment bases, and force discretisations are transformed consistently with the corresponding velocity-channel permutations. Thus, the equivariance regularisation is tied to the discrete symmetry group of the chosen lattice rather than to the full continuous rotation group.

S3. REPRESENTATIVE APPLICATIONS AND PERFORMANCE ANALYSIS OF THE PINO FRAMEWORK

In this section, we provide a systematic justification for the selection of applications and their corresponding hyperparameter settings used to represent the kinetic data-driven PINO framework. As shown in Table S1, different terms of the collision kernel, described in Section S2, are employed: specifically, the BGK collision kernel with the Smagorinsky subgrid-scale model and the MRT collision kernel. These applications notably include both 2D and 3D lattice models, namely the D2Q9 and D3Q19 models, respectively. Furthermore, the selected models represent various physical phenomena, including fluid-structure interaction, multiphase flow, and the phase-field model coupled with fluid dynamics. Detailed demonstrations for each example are provided in Table S1. In this work, we adopt a fixed and physically motivated set of hyperparameters for all experiments. The loss-function weights are chosen to reflect the relative importance of key physical quantities (e.g., density, velocity, and phase-field variables) in each problem, thereby enforcing the corresponding conservation or interface constraints during training. All models are trained for the same number of epochs using a consistent weighting strategy, avoiding case-specific fine tuning and ensuring a systematic and fully reproducible benchmark configuration. While we do not perform exhaustive hyperparameter optimisation, the framework allows interested users to further adjust the loss weights to emphasise specific physical targets or accuracy metrics as needed.

In Table S2, it shows the comparison between the LB simulation and the prediction time using the trained model. Specifically, our effective LB simulation time is defined as:

$$T_{\mathrm{LB}}^{\mathrm{eff}} = \frac{N_{\mathrm{data}}}{N_{\mathrm{total}}} T_{\mathrm{LB}}^{\mathrm{total}}, \tag{S29}$$

where $N_{\mathrm{data}} = L_x \times L_y$ is the total number of data grids, N_{total} is the domain simulation. $T_{\mathrm{LB}}^{\mathrm{total}}$ is the total simulation time. The reported acceleration is computed as the ratio between the effective LB simulation time and the ML prediction time. It is

important to emphasize that the LB simulations were executed using highly optimized C++ code, which is typically an order of magnitude (approximately 10×) faster than equivalent Python implementations. Therefore, if the LB simulations were conducted in Python, the observed acceleration would likely be more pronounced. Moreover, the acceleration is inherently tied to the nature of the ML predictor, which is capable of directly forecasting fluid states over large temporal strides. Instead of computing every intermediate timestep as in traditional LB solvers, the predictor effectively skips multiple timesteps, yielding substantial runtime reductions. All model training is performed on a single NVIDIA A100 80 GB GPU, while all inference benchmarks are executed on our inference hardware, an Intel(R) Xeon(R) Gold 6254 CPU @ 3.10 GHz. Since the reference LB solver is a CPU-only implementation, we executed it in single precision (FP32) to ensure a fair comparison with the neural predictor. A detailed comparison of the updated runtimes and speedups is provided in Table S2.

The memory usage reported in Table S3 reflects the peak device allocation observed during training. FNO models require substantially more GPU memory due to their global spectral convolution layers, which scale with the full spatial resolution of the domain. In contrast, UNO employs localized upsampling–downsampling operations, resulting in a significantly smaller memory footprint.

TABLE S3. GPU memory usage during training of the FNO and UNO models on a single NVIDIA A100 (80 GB) GPU. All models were trained in single precision (FP32) with batch size 32 for 100 epochs.

**Case**	**Model**	**GPU Memory (GB)**	**Training time (hh:mm:ss)**
Kármán vortex street (D3Q19)	FNO	39.9	03:20:16
	UNO	15.4	01:35:32
Liquid ligament breakup (D3Q19)	FNO	38.7	01:00:57
	UNO	15.9	00:26:50
Bubble adhesion (D2Q9)	FNO	29.1	00:15:34
	UNO	17.7	00:13:09

To quantify when the offline investment is recovered, we define the break-even number of inference queries as

$$N_{\text{break-even}} = \frac{T_{\text{data}} + T_{\text{train}}}{T_{\text{LB}}^{\text{eff}} - T_{\text{ML}}}, \tag{S30}$$

where T_{data} is the cumulative wall time used to generate the training dataset, T_{train} is the one-time training time, $T_{\text{LB}}^{\text{eff}}$ is the effective LB runtime for the same prediction window, and T_{ML} is the trained neural-operator inference time. We estimate T_{data} from the mean full-domain LB wall time and the number of simulations or snapshots used for each dataset. Using the timings reported above, the resulting estimate is:

**Case**	T_{data}	T_{train}	$T_{\text{data}} + T_{\text{train}}$	**Saving/query**	$N_{\text{break-even}}$
Kármán vortex street	2000×17.24 s $= 9.58$ h	1.59 h	11.17 h	$4.18 - 0.025 = 4.155$ s	$\approx 9.7 \times 10^3$
Liquid ligament breakup	440×14.88 s $= 1.82$ h	0.45 h	2.27 h	$0.31 - 0.043 = 0.267$ s	$\approx 3.1 \times 10^4$
Bubble adhesion	496×104.34 s $= 14.38$ h	0.22 h	14.60 h	$22.79 - 0.043 = 22.747$ s	$\approx 2.3 \times 10^3$

TABLE S4. Approximate offline-cost amortization for the neural operator surrogate. T_{data} is estimated from the mean full-domain LB wall time and the number of simulations or snapshots used to generate each dataset. The break-even count gives the number of inference queries after which the saved LB runtime exceeds the one-time data-generation and training cost.

The break-even count lies between approximately 2.3×10^3 and 3.1×10^4 inference queries across the three benchmarks; beyond this point the per-query saving accumulates and asymptotically approaches the inference-only acceleration reported in the main text. The relatively higher break-even count for the ligament case stems from its already inexpensive LB baseline, with a per-query saving of only 0.27 s, so the surrogate's value in that case is better read as enabling stable large-jump interfacial prediction than as a per-query cost reduction.

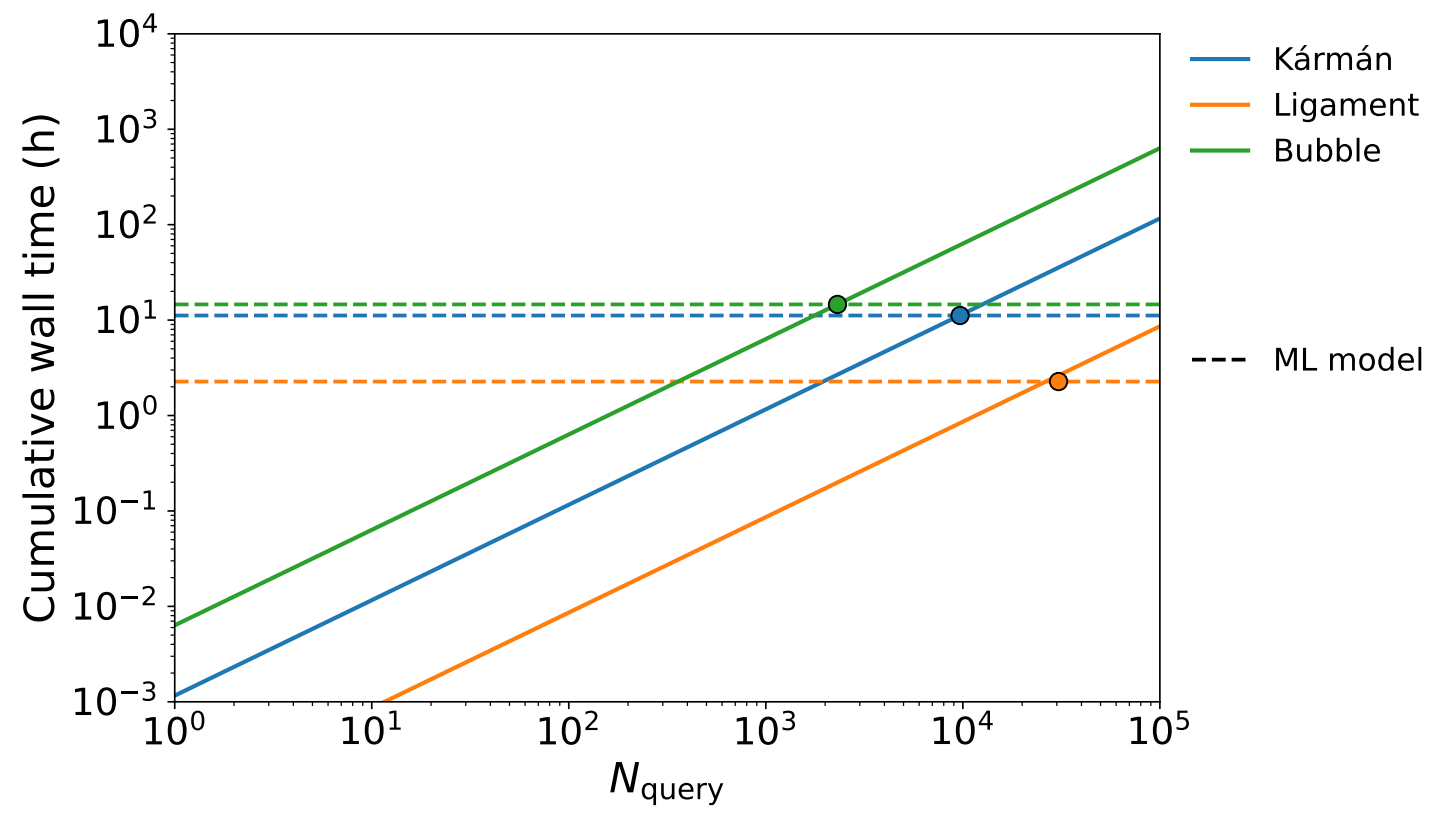

FIG. S1. Cumulative wall time versus the number of inference queries N_{query}, in log–log scale. Solid lines are LB baselines, which scale linearly in N_{query}; dashed lines are the trained ML model approximated by the one-time offline cost $T_{\text{data}} + T_{\text{train}}$, with the small per-query inference time neglected in the visualisation. Crossovers give the per-case break-even count $N_{\text{break-even}} \approx 9.7 \times 10^3$ for the Kármán case, 3.1×10^4 for the ligament case, and 2.3×10^3 for the bubble-adhesion case.

S4. NETWORK ARCHITECTURE DETAILS & TRAINING

We implement a physics-informed neural operator to model the long-time evolution of the lattice Boltzmann distribution functions $\{f_i\}$ across discrete velocity directions $\{\mathbf{c}_i\}$, without relying on explicit step-by-step temporal integration. To represent the neural operator $\mathcal{G}$ for LBE in spatiotemporal form, we model the evolution of the distribution functions using an integral operator over both space and time.

The input set is

$$f_{\text{in}} = \{f_i(\mathbf{x}, t')\}_{t' \in \mathcal{T}_{\text{in}},\, i=1}^{Q},$$

where Q denotes the number of discrete velocities, and the operator $\mathcal{G}$ maps this to the predicted output

$$\hat{f}_{\text{out}} = \{\hat{f}_i(\mathbf{x}, t)\}_{t \in \mathcal{T}_{\text{out}},\, i=1}^{Q}.$$

Formally, each predicted distribution is given by

$$\hat{f}_i(\mathbf{x}, t) = \sigma\left(\sum_{j=0}^{Q-1} \int_{\mathcal{T}_{\text{in}}} \int_{\mathcal{D}} K_{ij}^{\theta}(\mathbf{x}, t; \mathbf{x}', t')\, \phi_j(f_j(\mathbf{x}', t'))\, d\mathbf{x}' dt' + b_i(\mathbf{x}, t) \right), \tag{S31}$$

where $\mathcal{D}$ denotes the spatial domain; K_{ij}^{θ} is a learnable spatiotemporal kernel that models nonlocal interactions between spatial positions, times, and velocity components; ϕ_j is a

learnable pointwise feature map applied independently to each input channel; b_i is a learnable bias field; and σ is a pointwise nonlinearity such as the Gaussian error linear unit (GELU) [12].

S4.1 UNO architecture details

In this paper, we select the the U-shaped Neural operator for memory saving purpose. This choice is motivated by the universal approximation capabilities of Fourier series, especially in approximating solutions of PDEs [13]. The general architecture of the UNO is presented in Fig.S1. The architecture details are described in Table S5 to Table S7.

In the UNO architecture [14], the integral operator $\mathcal{G}$ is approximated by a hierarchical composition of convolutional transformations across multiple spatial resolutions. Formally, the operator is represented as:

$$\mathcal{G}^{\text{UNO}}(f) = \mathcal{Q}_\theta \circ \left(\mathcal{G}_\theta^{(L)} \circ \cdots \circ \mathcal{G}_\theta^{(1)}\right) \circ \mathcal{P}_\theta(f), \tag{S32}$$

where $\mathcal{P}_\theta$ lifts the input f to a latent feature space, each $\mathcal{G}_\theta^{(l)}$ is a local convolutional layer operating at scale l with downsampling or upsampling, and $\mathcal{Q}_\theta$ projects the final output back to the space of distribution functions. Skip connections between symmetric layers preserve spatial consistency across scales.

TABLE S5. Single Fourier block architecture. nc_{in}: number of in channels, nc_{out}: number of out channels, nm_{h}: number of Fourier modes for horizontal direction, nm_{w}: number of Fourier modes for vertical direction.

**Component**	**Layer**	**Parameters**
Fast Fourier Transformation (FFT)		
SpectralConv	Fully Connected (FC) Layer	$(\text{nc}_{\text{out}}, \text{nc}_{\text{in}}, \text{nm}_{\text{h}}, \text{nm}_{\text{w}})$
Skip	2D Convolution	$(\text{nc}_{\text{in}}, \text{nc}_{\text{out}}, 1, 1)$
Inverse FFT		
Activation	GELU	

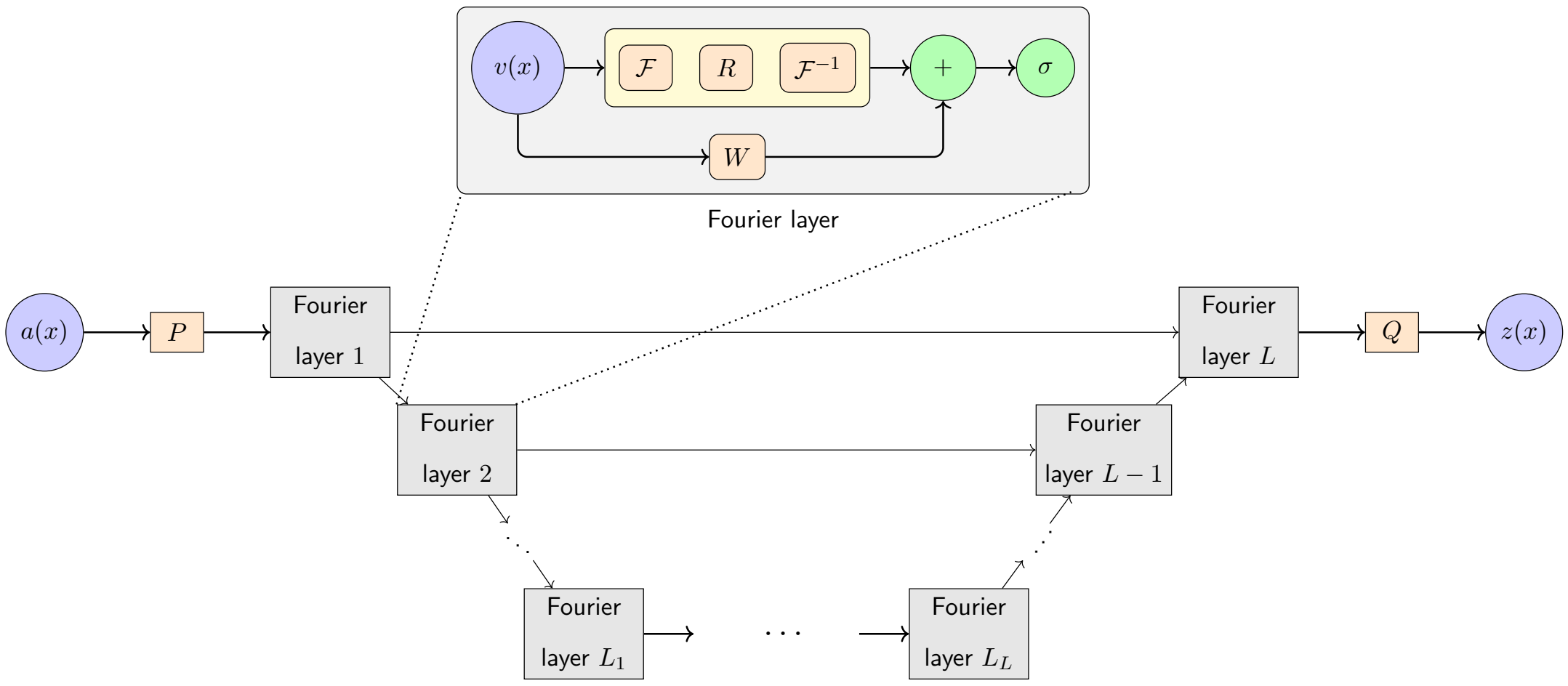

FIG. S2. The general UNO architecture for LBE. The input distribution function $a(x)$ is first projected by P into a higher-dimensional feature space. The core of the network consists of a sequence of Fourier layers, each performing a spectral transform ($\mathcal{F}$), linear transformation (R), inverse transform ($\mathcal{F}^{-1}$), nonlinear activation (σ), and residual connection with weights W. Finally, the processed features are mapped back to the output distribution $z(x)$ through Q.

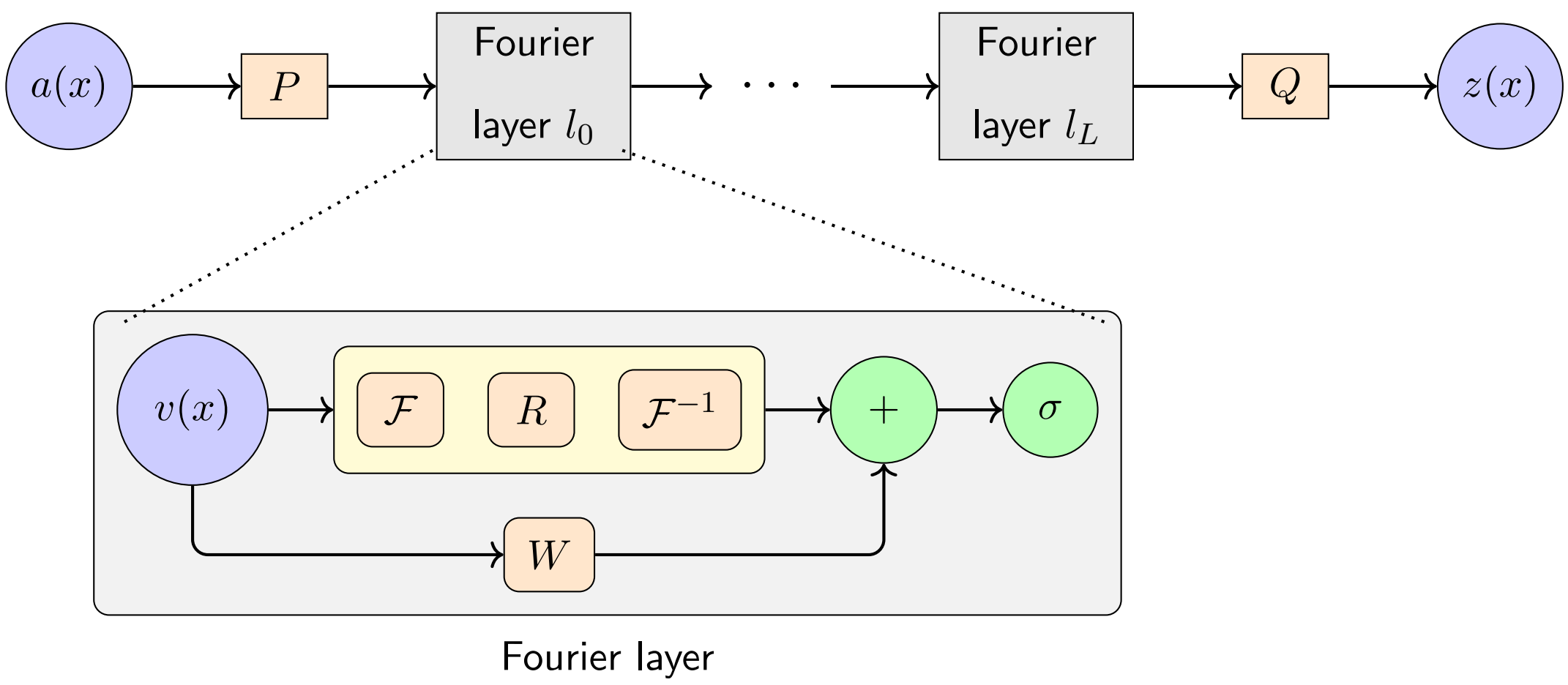

FIG. S3. The general FNO architecture for LBE. The input distribution function $a(x)$ is lifted by P, passed through a stack of Fourier layers with spectral transforms ($\mathcal{F}$, R, $\mathcal{F}^{-1}$), nonlinear activations (σ), and residual connections (W), and finally projected back to the output $z(x)$ by Q.

TABLE S6. Model architecture for von Kármán vortex street input data with shape (c, h, w) where c is the number of in channels, h is the number of grid points in the horizontal direction, w is the number of grid points in the vertical direction. The total parameters are 12 million.

Component	Layer	Parameters
Encoder	Convolution 2D	$(\text{nc}_{\text{in}} = c, \text{nc}_{\text{out}} = 256, 1, 1)$
	Convolution 2D	$(\text{nc}_{\text{in}} = 256, \text{nc}_{\text{out}} = 128, 1, 1)$
	Fourier Block	$(\text{nc}_{\text{in}} = 128, \text{nc}_{\text{out}} = 32, \text{nm}_{\text{h}} = \frac{h}{2}, \text{nm}_{\text{w}} = \frac{w}{2})$
	Fourier Block	$(\text{nc}_{\text{in}} = 32, \text{nc}_{\text{out}} = 64, \text{nm}_{\text{h}} = \frac{h}{4}, \text{nm}_{\text{w}} = \frac{w}{4})$
	Fourier Block	$(\text{nc}_{\text{in}} = 64, \text{nc}_{\text{out}} = 128, \text{nm}_{\text{h}} = \frac{h}{8}, \text{nm}_{\text{w}} = \frac{w}{8})$
Bottle neck	Fourier Block	$(\text{nc}_{\text{in}} = 128, \text{nc}_{\text{out}} = 128, \text{nm}_{\text{h}} = \frac{h}{8}, \text{nm}_{\text{w}} = \frac{w}{8})$
Decoder	Fourier Block	$(\text{nc}_{\text{in}} = 128, \text{nc}_{\text{out}} = 64, \text{nm}_{\text{h}} = \frac{h}{8}, \text{nm}_{\text{w}} = \frac{w}{8})$
	Fourier Block	$(\text{nc}_{\text{in}} = 64, \text{nc}_{\text{out}} = 32, \text{nm}_{\text{h}} = \frac{h}{4}, \text{nm}_{\text{w}} = \frac{w}{4})$
	Fourier Block	$(\text{nc}_{\text{in}} = 32, \text{nc}_{\text{out}} = 128, \text{nm}_{\text{h}} = \frac{h}{2}, \text{nm}_{\text{w}} = \frac{w}{2})$
	Convolution 2D	$(\text{nc}_{\text{in}} = 128, \text{nc}_{\text{out}} = 256, 1, 1)$
	Convolution 2D	$(\text{nc}_{\text{in}} = 256, \text{nc}_{\text{out}} = c, 1, 1)$

TABLE S7. Model architecture for example 2 and 3 input data with shape (c, h, w) where c is the number of in channels, h is the number of grid points in the horizontal direction, w is the number of grid points in the vertical direction.

Component	Layer	Parameters
Encoder	Convolution 2D	$(\text{nc}_{\text{in}} = c, \text{nc}_{\text{out}} = 256, 1, 1)$
	Convolution 2D	$(\text{nc}_{\text{in}} = 256, \text{nc}_{\text{out}} = 128, 1, 1)$
	Fourier Block	$(\text{nc}_{\text{in}} = 128, \text{nc}_{\text{out}} = 64, \text{nm}_{\text{h}} = \frac{h}{2}, \text{nm}_{\text{w}} = \frac{w}{2})$
Bottle neck	Fourier Block	$(\text{nc}_{\text{in}} = 64, \text{nc}_{\text{out}} = 64, \text{nm}_{\text{h}} = \frac{h}{8}, \text{nm}_{\text{w}} = \frac{w}{8})$
Decoder	Fourier Block	$(\text{nc}_{\text{in}} = 64, \text{nc}_{\text{out}} = 128, \text{nm}_{\text{h}} = \frac{h}{8}, \text{nm}_{\text{w}} = \frac{w}{8})$
	Convolution 2D	$(\text{nc}_{\text{in}} = 128, \text{nc}_{\text{out}} = 256, 1, 1)$
	Convolution 2D	$(\text{nc}_{\text{in}} = 256, \text{nc}_{\text{out}} = c, 1, 1)$

S4.2 FNO architecture details

The general architecture of the FNO for LBE is presented in Fig.S2. The ablation FNO architecture detail is presented between Table S8 to Table S10. In the FNO [15],

the operator $\mathcal{G}$ is constructed as a composition of Fourier layers, each approximating the integral operator via spectral convolution. The full operator is defined as:

$$\mathcal{G}^{\text{FNO}}(f) = \mathcal{Q}_\theta \circ \left(\mathcal{F}_\theta^{(L)} \circ \sigma \circ \cdots \circ \mathcal{F}_\theta^{(1)} \circ \sigma\right) \circ \mathcal{P}_\theta(f), \tag{S33}$$

where $\mathcal{P}_\theta$ and $\mathcal{Q}_\theta$ are pointwise lifting and projection maps, σ is a nonlinear function, and each $\mathcal{F}_\theta^{(l)}$ is a spectral convolution layer defined by:

$$\mathcal{F}_\theta^{(l)}(f)(\mathbf{x}) = W_\theta^{(l)} f(\mathbf{x}) + \mathcal{F}^{-1}\left(R_\theta^{(l)}(\kappa) \cdot \mathcal{F}(f)(\kappa)\right), \tag{S34}$$

where $W_\theta^{(l)}$ is a learnable pointwise linear transformation, $\mathcal{F}$ and $\mathcal{F}^{-1}$ denote the forward and inverse Fourier transforms, and $R_\theta^{(l)}(\kappa)$ are the learnable spectral multipliers in layer l. This composition enables the operator to model global interactions efficiently across the spatial domain.

TABLE S8. Single Fourier Block architecture used in the FNO models, where m_h is the number of modes for the horizontal direction and m_w is the number of modes for the vertical direction. Each block consists of a forward FFT, spectral convolution in Fourier space via a Tucker linear layer, a parallel skip connection implemented with 1D convolution, and a nonlinear channel MLP. The outputs are combined through a skip gate, followed by an inverse FFT and GELU activation.

**Component**	**Layer**	**Parameters**
Fast Fourier Transformation (FFT)	—	—
Spectral Convolution	Tucker Linear Layer	$(n_{\text{out}},\ n_{\text{in}},\ \text{modes} = (m_h,\ m_w))$
Skip Connection	Convolution 1D	$(n_{\text{in}},\ n_{\text{out}},\ \text{kernel} = 1,\ \text{stride} = 1)$
Nonlinearity	Channel MLP	Convolution 1D $(n_{\text{in}} \rightarrow \frac{n_{\text{in}}}{2} \rightarrow n_{\text{in}})$
MLP Skip Gate	SoftGating()	—
Inverse FFT	—	—
Activation	GELU	—

TABLE S9. Model architecture for von Kármán vortex street and cylindrical ligament breakup, both with 62 millions total parameters. The input data have shape (c, h, w), where c is the number of in channels, h is the number of grid points in the horizontal direction, w is the number of grid points in the vertical direction.

**Component**	**Layer**	**Parameters**
Lifting	Convolution 1D	$(n_{\text{in}} = c,\ n_{\text{out}} = 256, 1, 1)$
	Convolution 1D	$(n_{\text{in}} = 256,\ n_{\text{out}} = 128,\ 1, 1)$
Fourier Blocks	Fourier Block	$(n_{\text{in}} = 128,\ n_{\text{out}} = 128,\ \text{modes} = (32,\ 32))$
	Fourier Block	$(n_{\text{in}} = 128,\ n_{\text{out}} = 128,\ \text{modes} = (32,\ 32))$
	Fourier Block	$(n_{\text{in}} = 128,\ n_{\text{out}} = 128,\ \text{modes} = (32,\ 32))$
	Fourier Block	$(n_{\text{in}} = 128,\ n_{\text{out}} = 128,\ \text{modes} = (32,\ 32))$
	Fourier Block	$(n_{\text{in}} = 128,\ n_{\text{out}} = 128,\ \text{modes} = (32,\ 32))$
	Fourier Block	$(n_{\text{in}} = 128,\ n_{\text{out}} = 128,\ \text{modes} = (32,\ 32))$
	Fourier Block	$(n_{\text{in}} = 128,\ n_{\text{out}} = 128,\ \text{modes} = (32,\ 32))$
Projection	Convolution 1D	$(n_{\text{in}} = 128,\ n_{\text{out}} = 128,\ 1, 1)$
	Convolution 1D	$(n_{\text{in}} = 128,\ n_{\text{out}} = c,\ 1, 1)$

TABLE S10. Model architecture for bubble adhesion, with 27 millions total parameters. The input data have shape (c, h, w), where c is the number of in channels, h is the number of grid points in the horizontal direction, w is the number of grid points in the vertical direction.

**Component**	**Layer**	**Parameters**
Lifting	Convolution 1D	$(n_{\text{in}} = c,\ n_{\text{out}} = 256,\ 1, 1)$
	Convolution 1D	$(n_{\text{in}} = 256,\ n_{\text{out}} = 128,\ 1, 1)$
Fourier Blocks	Fourier Block	$(n_{\text{in}} = 128,\ n_{\text{out}} = 128,\ \text{modes} = (32,\ 32))$
	Fourier Block	$(n_{\text{in}} = 128,\ n_{\text{out}} = 128,\ \text{modes} = (32,\ 32))$
	Fourier Block	$(n_{\text{in}} = 128,\ n_{\text{out}} = 128,\ \text{modes} = (32,\ 32))$
Projection	Convolution 1D	$(n_{\text{in}} = 128,\ n_{\text{out}} = 128,\ 1, 1)$
	Convolution 1D	$(n_{\text{in}} = 128,\ n_{\text{out}} = c,\ 1, 1)$

S4.3 Traning proceedure

We have provided our training procedure accordingly with three phases:

Phase 1: Data Preparation

Input: LBM dataset $\mathcal{D}$ with distribution fields $f_i(\mathbf{x}, t)$

Goal: Construct input–target training pairs for operator learning

1. Split $\mathcal{D}$ along the temporal axis into train/validation/test.
2. For each split, slide a temporal window of size $S+T$.
3. Construct:

$$X = \{f_{t-S+1}, \ldots, f_t\}, \qquad Y = \{f_{t+1}, \ldots, f_{t+P}\}.$$

4. Stack S distribution fields along the channel dimension $\Rightarrow X \in \mathbb{R}^{(19S)\times H\times W}$.
5. Save datasets into PyTorch `Dataset/DataLoader`.

Phase 2: Model & Loss Setup

1. Initialize UNO model f_θ with:

$$\text{input channels} = 19S, \quad \text{output channels} = 19P.$$

2. Define losses:
   - Data fidelity: $\mathcal{L}_{\mathrm{L2}}$, $\mathcal{L}_{\mathrm{H1}}$
   - Kinetic constraints: density $\mathcal{L}_\rho$, velocity $\mathcal{L}_u$
   - Global equivariance (D4 symmetry for example):

$$\mathcal{L}_{\mathrm{eq}} = \frac{1}{8} \sum_{g\in D_4} \|f_\theta(g(X)) - g(Y)\|_2.$$

     For D3Q19 cases, the same construction is applied using the 24 rotations in the proper octahedral rotation group O, replacing $\frac{1}{8}\sum_{g\in D_4}$ by $\frac{1}{24}\sum_{g\in O}$ with the corresponding velocity-channel permutations.
3. Set weighted total loss.
4. Initialize Adam optimizer with cyclic learning-rate schedule.

Phase 3: Training Loop

For epoch $i = 1 \ldots Epochs$:

1. Select learning rate from the periodic schedule.
2. Set model to training mode.
3. For each minibatch (X, Y):
   (a) Move tensors to GPU (FP32).
   (b) Compute prediction: $\hat{Y} = f_\theta(X)$.
   (c) Evaluate all loss components.
   (d) Compute total loss $\mathcal{L}$.
   (e) Backpropagate, clip gradients, optimizer step.
4. Set model to evaluation mode.
5. Compute validation losses over all batches.

S5. DATA GENERATION

In this section, we present three test cases to illustrate the data preparation process for our training. The test cases include the von Kármán vortex street, cylindrical ligament breakup, and bubble adhesion.

S5.1 Von Kármán vortex street set up

To prepare the von Kármán vortex street data, we apply the Bhatnagar-Gross-Krook model with a Smagorinsky subgrid-scale collision kernel (see Table S1). Data generation is performed using the D3Q19 lattice model. The full domain size is with the grid size of $110 \times 80 \times 3$. The input data has shape $L_i \times L_x \times L_y$, where L_i, L_x, and L_y are the data dimensions in the discretisation number of the lattice model, stream-wise, and span-wise dimensions. A full dataset for a single time snapshot is $19 \times 80 \times 80$. The relaxation time τ is set to $\tau = 0.505$. The training data are constructed with 2000 snapshots from the low-resolution (LR) data, resulting in the total dimension of the dataset equal to $2000 \times 19 \times 80 \times 80$. The input to the neural network architecture consists of a stack of 5

time-series snapshots from $t_0 = 1\Delta t$ to $t_5 = 5\Delta t$, where $\Delta t = n\,\Delta t$ with $n = 50$, with each time frame representing 50 collision intervals. The output of the neural architecture is a single prediction at t_6, corresponding to 300 Δt, effectively "jumping" over 300 collision intervals in the system.

S5.2 Cylindrical ligament breakup set up

In the cylindrical ligament breakup case, the kinetic framework, driven by LB data, is utilised to solve the multiphase ligament breakup problem [16, 17]. We employ the D3Q19 model with the MRT collision kernel and a Shan-Chen forcing term, as detailed in Sections S2 and S3. The simulations are conducted in a three-dimensional space for the breakup of a cylindrical ligament, with a domain size of $L_i \times L_x \times L_y \times L_z = 19 \times 48 \times 128 \times 48$ LU. The initial radius of the cylinder is set to $R_0 = 7$ lattice units. The ligament surface is perturbed according to the function $r(x) = R_0 + \delta \sin\left(\frac{4\pi x}{2\lambda} + \phi_s\right)$, where $\delta = 2.5$ LU represents the amplitude of the perturbation, λ the wavelength, and ϕ_s the phase shift. The periodic boundary condition is set in all x, y, z directions. The liquid cylinder is initialised with a density of $\rho_1 = 2.11633$ in LU, while the surrounding gas phase is assigned a density of $\rho_2 = 0.083408$ LU. The Shan-Chen interaction constant $\mathcal{G}_{sc}$ is set to $\mathcal{G}_{sc} = -5.3$, which governs the two-phase interactions. The relaxation time τ, which controls the viscous dissipation in the system, is set to $\tau = 0.6767$ LU. To generate the dataset, we vary the position of the ligament center along the x-axis, denoted by $x_c \in [14 : 2 : 34]$, and the phase shift of the sinusoidal surface perturbation, ϕ_s, is varied in the range $\phi_s \in [0.04 : 0.05 : 2)$. In total, $N_{\text{total}} = 440$ simulations were performed. Each simulation consists of 14 time frames, uniformly distributed over the time evolution of the ligament breakup process. We select the xz plane located in the middle of the y direction. During initialisation, the equilibrium density distribution function f^{eq} is assigned to each simulation, following the Maxwell-Boltzmann distribution, based on the macroscopic density ρ and velocity $\mathbf{c}$ at each lattice point. This ensures that the system begins to maintain thermodynamic equilibrium before the ligament breakup dynamics unfold.

S5.3 Bubble adhesion set up

In the bubble adhesion scenario, we employ the D2Q9 lattice model for both the hydrodynamic evolution and the phase-field model. The hydrodynamic behaviour, specifically

the laminar flow over a cylinder with low Reynolds numbers, is captured by the density distribution function $\mathbf{f}$. This function governs the fluid motion and evolves according to the LB framework. Simultaneously, the evolution of the phase field model, representing the dynamics of the bubble, surrounding fluid, and cylinder, is described by the density distribution function $\mathbf{g}$. The coupling between the fluid dynamics and the phase-field model is achieved through modifications to the equilibrium distribution functions, as shown in Eq. S13.

All simulations are performed on the 2D plane at a domain size of $L_i \times L_x \times L_y = 9 \times 100 \times 300$ LU. For data reduction purposes, we only select the region where the phase-field interaction occurs. The selected region is the $9 \times 84 \times 78$ grid size. Both liquid and gas that have their own relaxation times τ_f and τ_g are set to 1. The radius of the cylinder is set to 10 LU. The bubble is initialised with radius 10 LU. The phase value of the wall is 1.5 LU, the bubble is set to 1.0 LU, and the liquid is set to -1.0 LU. The centre of the cylinder is located at (x_d, y_d) where $x_d \in [26 : 1 : 56]$,$y_d \in [29 : 49]$. Adjusting x_d and y_d, we performed simulations $N_{\text{total}} = 446$ times.

During initialisation, the equilibrium density distribution function for both $\mathbf{f}$ and $\mathbf{g}$ follows the Maxwell-Boltzmann distribution, based on the macroscopic property at each lattice point.

S6. ADDITIONAL RESULTS

In this section, we present additional results for the three applications: the von Kármán vortex street, ligament breakup and bubble adhesion. First, we demonstrate the low-resolution versus super-resolution results for the von Kármán vortex street. Next, we show the evolution process and error comparison for the cylindrical ligament breakup and bubble adhesion cases.

S6.1 Von Kármán vortex street: low resolution vs super resolution

In this section, we aim to demonstrate our model's capability for super-resolution within a kinetic data framework. Specifically, by super-resolution, we refer to the model's ability to upscale predictions from a pre-trained, low-resolution kinetic dataset (domain size of 80×80 LU) to high-resolution dynamics (160×160 LU) in an autoregressive manner. In particular, this is achieved without requiring additional fine-tuning or transfer

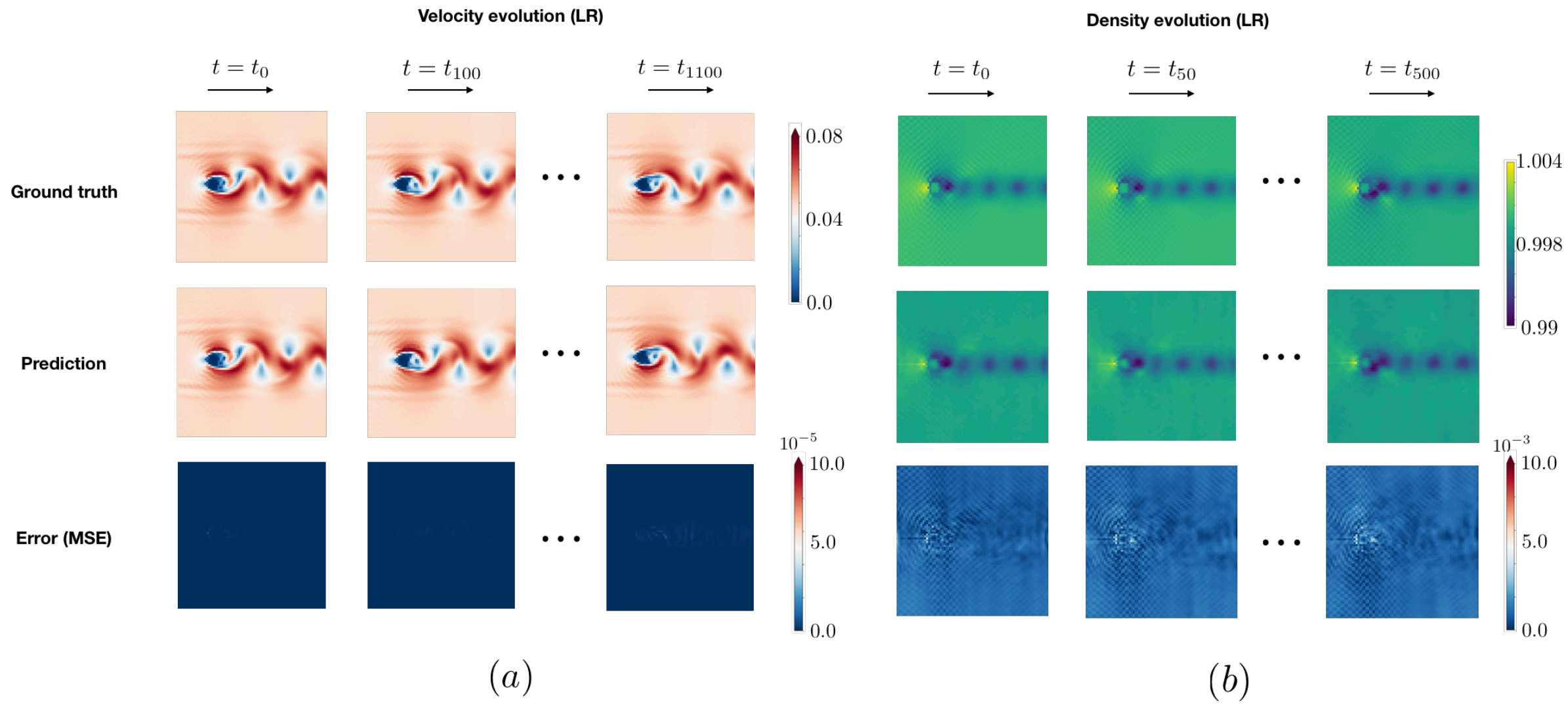

FIG. S4. The low resolution predictions are derived from the kinetic data. In Panel (a), the top row shows the ground truth for the velocity field evolution, the middle row presents the reconstructed velocity field predicted using MSE+Phys from kinetic data, and the bottom row displays the mean square error (MSE) between the ground truth and the prediction. In Panel (b), the top row shows the ground truth for the density field evolution, the middle row displays the reconstructed density field from kinetic data with MSE+Phys prediction, and the bottom row shows the MSE between the ground truth and the prediction.

learning. We begin by examining the low-resolution predictions and associated error metrics under the MSE+Phys constraint, providing a foundation for assessing super-resolution performance.

1. Low resolution prediction

As shown in Fig. S4, we present the predicted evolution at the macroscopic level derived from the kinetic data, where each time frame comprises $Q \times 80 \times 80$ LB data with $Q = 19$. By predicting data evolution at the kinetic level, we reconstruct the corresponding macroscopic evolution. In particular, each predicted time frame surpasses the single-step forward limitation with $\Delta t = n\Delta t$ where $n = 50$, equivalent to 50 steps of LB simulation.

In Figs. S4 (a) and S4 (b), we analyse the predictions for both velocity and density evolution at the macroscopic scale. Figure S4 (a) compares the evolution of the velocity field between the ground truth and the predictions, with the error quantified by the MSE.

Up to $t = t_{1100}$, the predictions closely align with the ground truth, with a maximum MSE error of approximately 1.0×10^{-4}. In particular, with the inclusion of our MSE+Phys constraint, the density field is also accurately captured in the LR data, as shown in Fig. S4 (b). Due to the weak compressibility of the numerical solution in the lattice Boltzmann equation [18], the predicted field exhibits minor density oscillations, represented by dark blue points, which are in good agreement with the ground truth. It is important to note that all results presented in this paper are in dimensionless units, which can be easily converted to physical units as described in the section of section S1.7. The MSE error indicates that the maximum MSE error for the density field is near 1×10^{-3}.

2. Super resolution prediction

We proceed to test the super-resolution (SR) performance of the trained model using low-resolution (LR) data. The simulation configuration remains consistent with that of the LR simulations, with the only difference being the simulation size, now set to 160×160 lattices in 2D. The relaxation time τ is set to $\tau = 0.5025$, differing from the LR simulations. Successfully capturing the simulation's evolution under this new configuration would indicate that super-resolution is effective at the kinetic level, even with a change in relaxation time and without any additional training on SR data.

We used a stack of 16 simulation snapshots in a time series as input to predict a single timestep at $t = t_{17}$. Subsequent steps are predicted auto-aggressively without corrections. For the SR configuration, each prediction snapshot has $\Delta t = 160\,\Delta t$, compared to $\Delta t = 50\,\Delta t$ in the LR data. Interestingly, our simulation results show strong alignment with the SR data, as demonstrated in Fig. S5.

In Figure S5 (a), the ground truth of the velocity field exhibits fewer spurious currents [19] compared to the LR data shown in Fig. S4(a), as the higher resolution reduces numerical errors. For the SR predictions, the model successfully predicted the evolution of the SR velocity field with an MSE of approximately 5.0×10^{-5}. Figure S5(b) illustrates the density evolution prediction for the SR data. The model effectively captures the overall evolution of the density field; however, some discrepancies are observed as spurious yellow regions near the darker points. Overall, the density field prediction shows a maximum MSE difference of 1.0×10^{-2}. Next, we further analyse why our model can predict SR data using the neural networks model obtained via the LR data.

Figure S6 illustrates the latent spaces in the bottleneck of the training models, referred

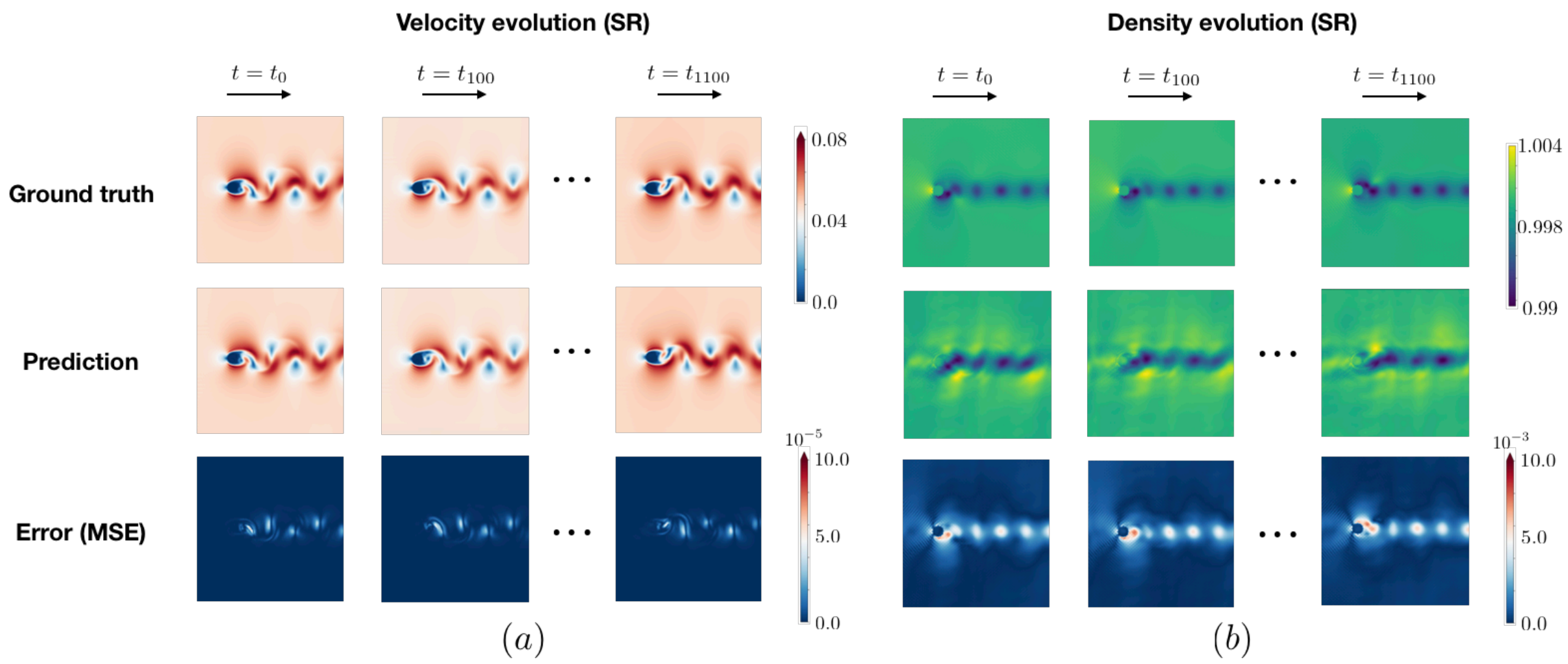

FIG. S5. The super resolution (SR) predictions are derived from kinetic-level predictions at time frames $t = t_0$, $t = t_{100}$, and $t = t_{1100}$. Panel (a) shows the ground truth, the prediction using the MSE+Phys constraint, and the MSE error for the SR velocity field evolution reconstructed from kinetic data. Panel (b) displays the ground truth, the prediction using the MSE+Phys constraint, and the MSE error for the SR density field evolution computed by kinetic data.

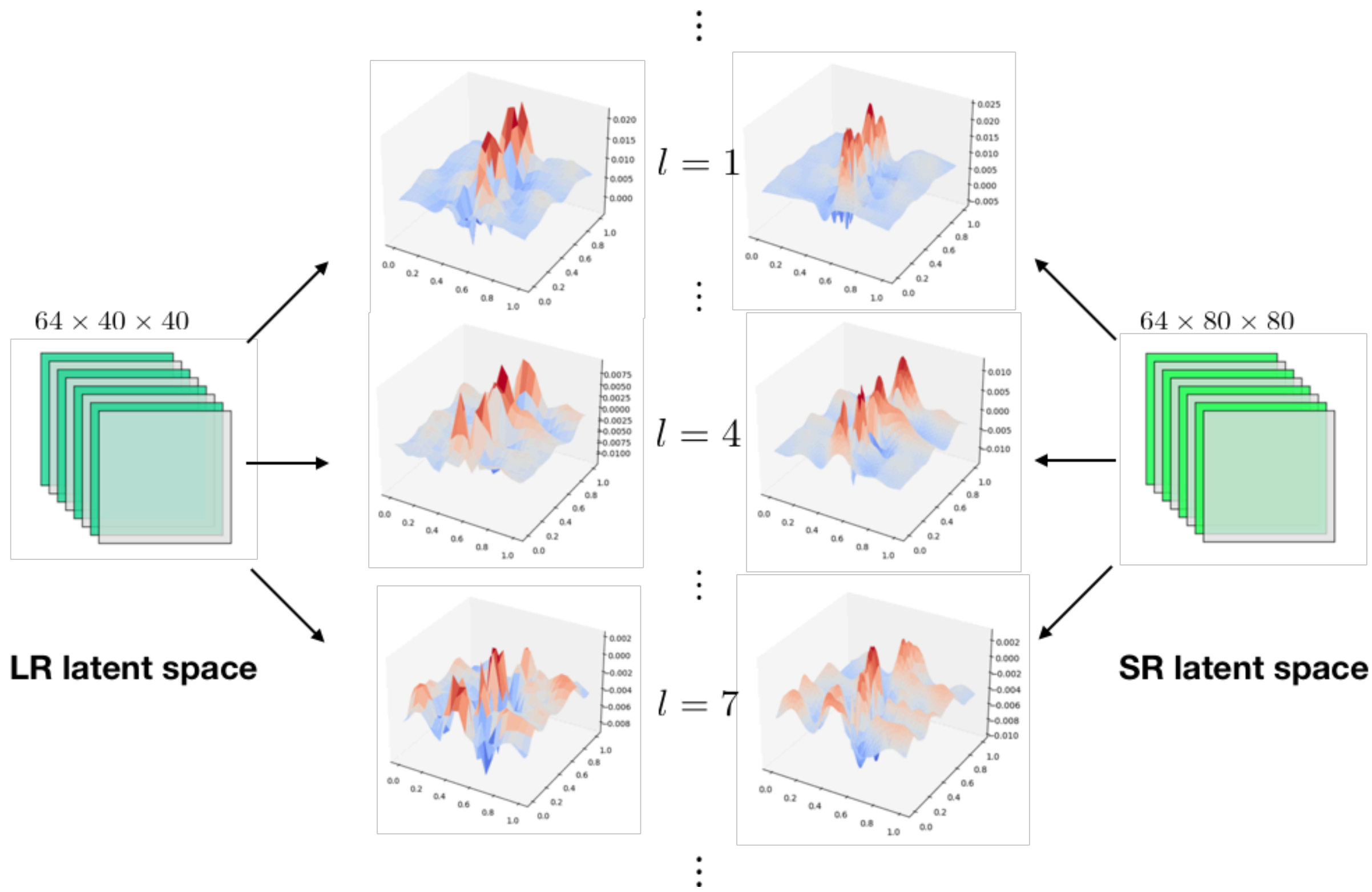

FIG. S6. Comparison of LR and SR latent spaces. The backbone dimensions for LR and SR are set to $L_l \times L_x \times L_y$, where $L_l = 64$, $L_x = 40$, $L_y = 40$ for LR, and $L_l = 64$, $L_x = 80$, $L_y = 80$ for SR. Three representative latent spaces are randomly selected with $l = 1, 4, 7$.

to as the LR latent space and the SR latent space. The LR latent space has dimensions $64 \times 40 \times 40$, corresponding to $L_l \times L_x \times L_y$, where L_l represents the number of layers at the bottleneck. In comparison, the latent space of SR is constructed with dimensions $64 \times 80 \times 80$. For analysis, we randomly selected three layers, specifically $l = 1$, $l = 4$, and $l = 7$, from each model.

The LR latent space closely resembles the SR latent space, suggesting that the SR latent space can be interpreted as a "grid-refined" version of the LR latent space. This similarity in structure supports the successful prediction of SR data by indicating analogous patterns across the latent spaces.

S6.2 Cylindrical ligament breakup: error comparison

In this example, we demonstrate both the ligament breakup process as shown in Fig. S7 and the evolution of the ligament breakup error evolution for the MSE constraint and the MSE+Phys constraint in Fig. S16.

In Fig. S7 and Fig. S16, both the MSE constraint and the MSE+Phys constraint successfully capture the breakup process. The dimensionless parameter $t^* = t/t_{\text{end}}$ represents the ratio between the current time scale and the final time scale. However, as shown in Fig. S12, the MSE+Phys constraint exhibits greater stability during training, whereas the MSE constraint alone is more prone to becoming trapped in local minima.

S6.3 Bubble adhesion: error comparison

In this example, we demonstrate both the bubble adhesion process (Fig. S9) and the evolution of the error for the MSE constraint and the MSE+Phys constraint (Fig. S17). Overall, the MSE+Phys constraint outperforms the MSE constraint.In Fig. S9, the MSE+Phys constraint exhibits excellent alignment and is able to capture the remerging process after bubble breakup, however, the MSE constraint struggles to merge the bubble at $t^* = 1.0$. In Fig. S17, the MSE+Phys constraint exhibits minimal error accumulation as t^* approaches 1.0, whereas the MSE constraint shows significant error accumulation, particularly during the bubble merging process.

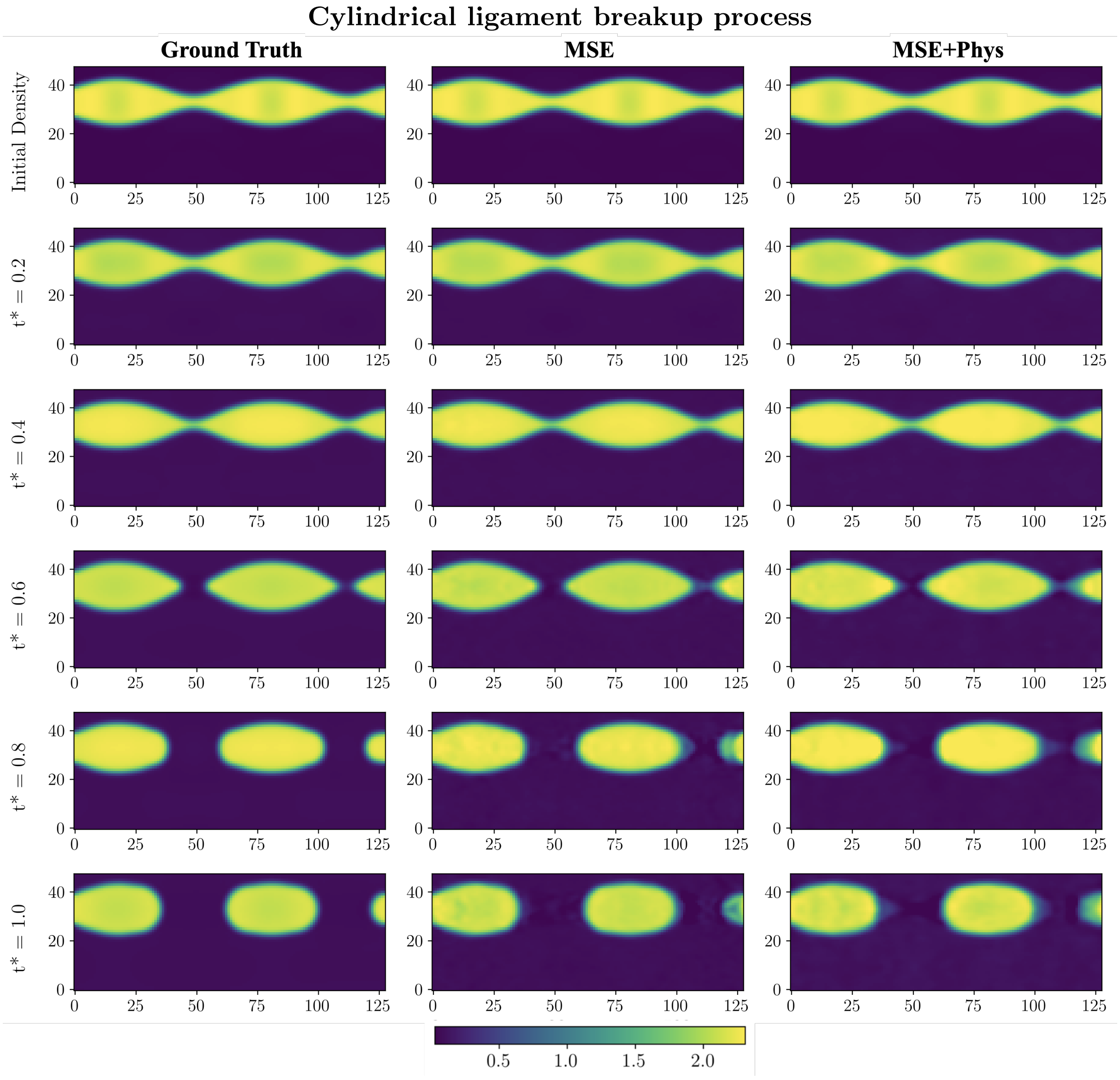

FIG. S7. Demonstration of the cylindrical ligament breakup process. The left, middle, and right columns represent the ground truth evolution, the MSE-predicted evolution, and the MSE+Phys-predicted evolution, respectively. All results are reconstructed from kinetic data. The first row shows the initial density for all three cases. The figure illustrates the breakup process at various dimensionless times, t^*, capturing the transformation of the ligament from a sinusoidal shape into droplets.

S6.4 Training evolution comparison for selected applications

Figures S11 to S13 show the evolution of training loss under the pure MSE constraint and the combined MSE and Phys constraint (integrating conservation laws and symmetry-equivariant groups). In the last two cases, the MSE+Phys constraint outper-

forms the MSE constraint. However, in the Kármán vortex street case, both the MSE and MSE+Phys constraints exhibit a similar progression in training. Figure S11 illustrates the L_2-normal error as a function of the epoch. Due to uncertainties during the training process, the training loss is evaluated using an ensemble approach. We systematically

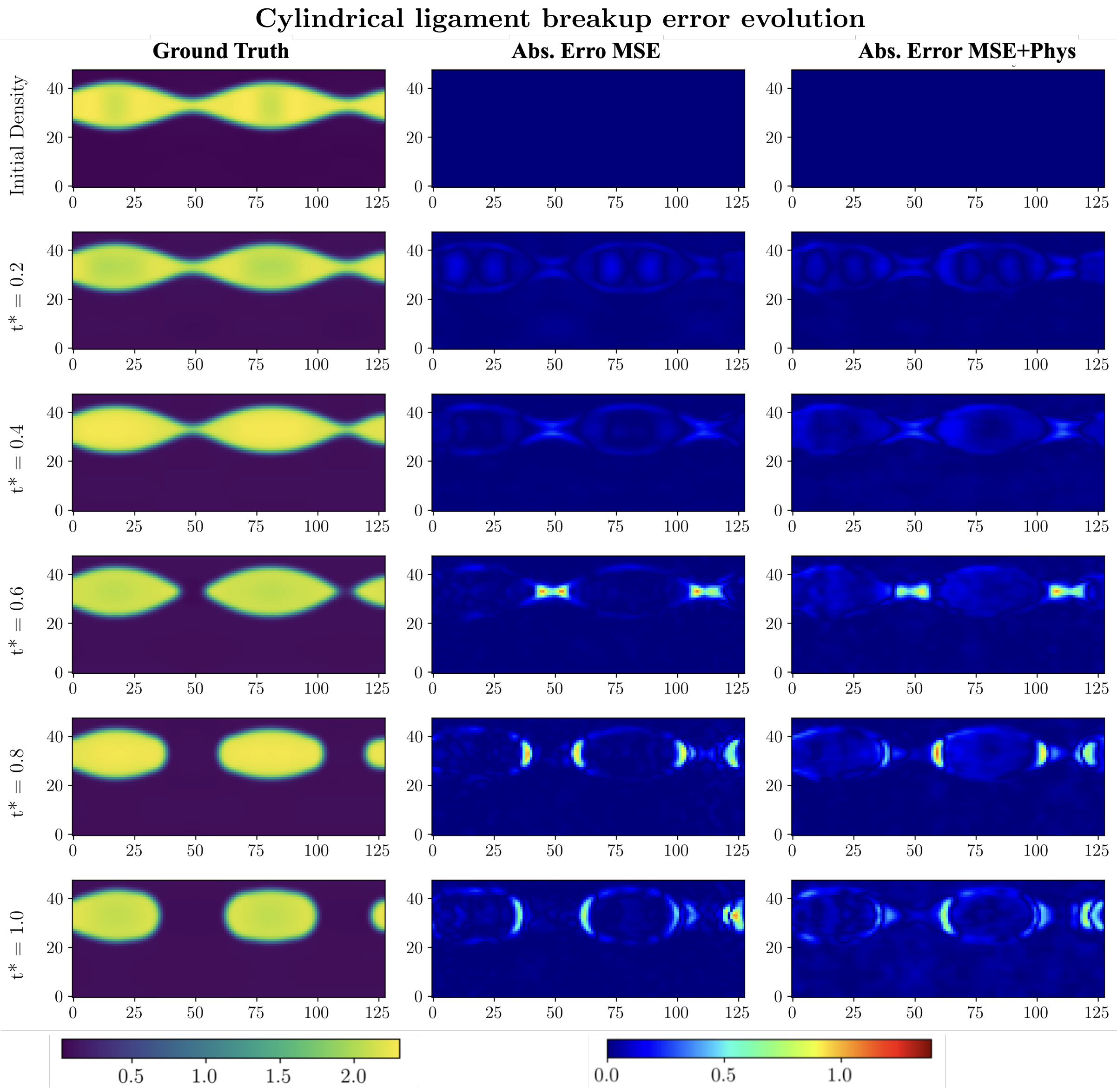

FIG. S8. Demonstration of the cylindrical ligament breakup error evolution. The left, middle, and right columns show the ground truth, the MSE-predicted absolute error, and the MSE+Phys-predicted absolute error, respectively. All results are reconstructed from kinetic data, and then make further evaluations. The first row presents the multiphase ligament and initial absolute error. The figure captures the ligament breakup into droplets over dimensionless times, t^*, with error evolution for the MSE and the MSE+Phys shown in the right columns.

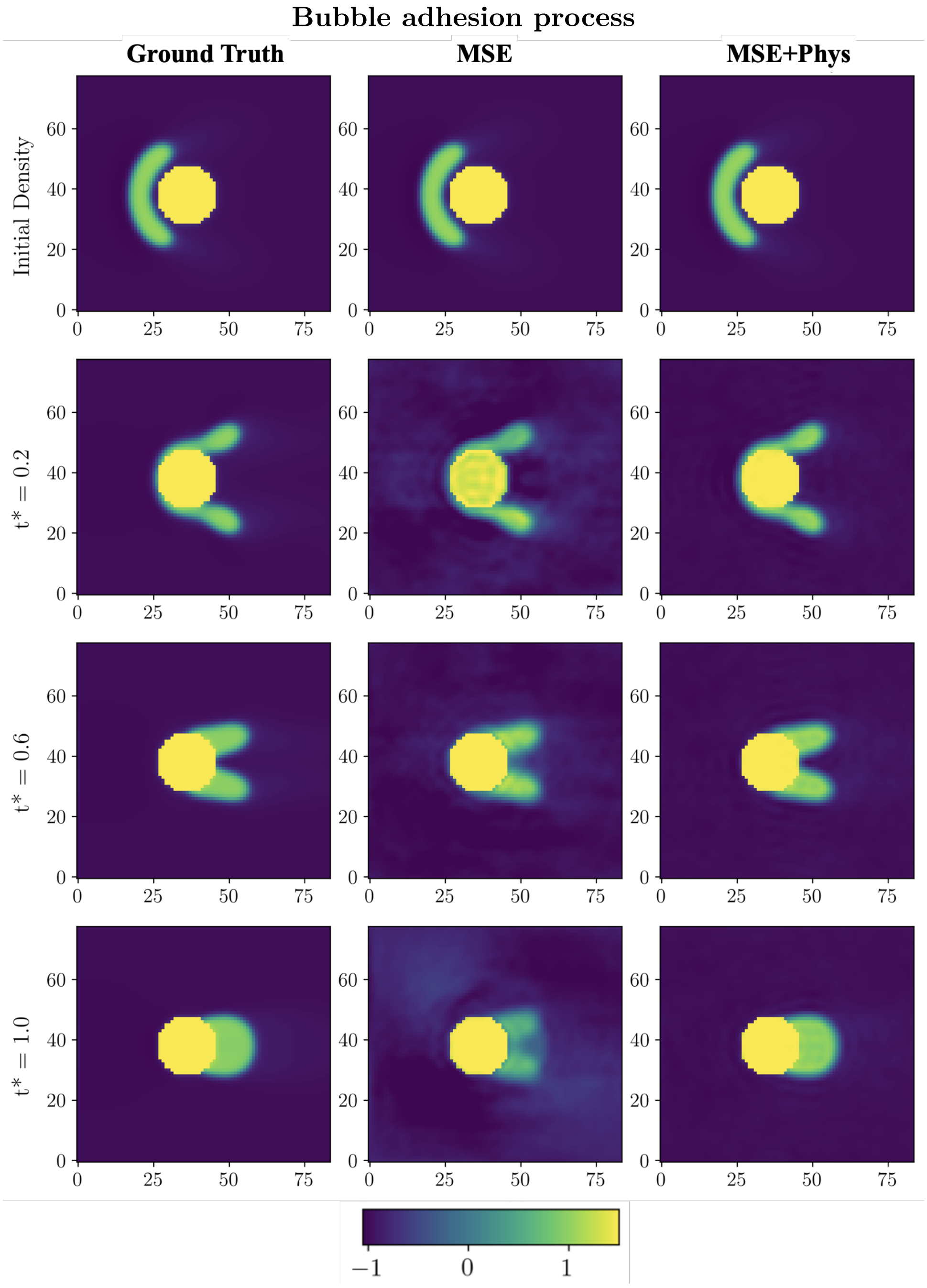

FIG. S9. Illustration of the bubble adhesion process. The left, middle, and right columns represent the ground truth evolution, the MSE-predicted evolution, and the MSE+Phys-predicted evolution all reconstructed from kinetic data. The first row shows the initial phase field from kinetic data. At various dimensionless times, t^*, the bubble comes across the cylinder, breaks into two smaller bubbles, and then merges. The MSE constraint struggles to reconstruct the merging process accurately.

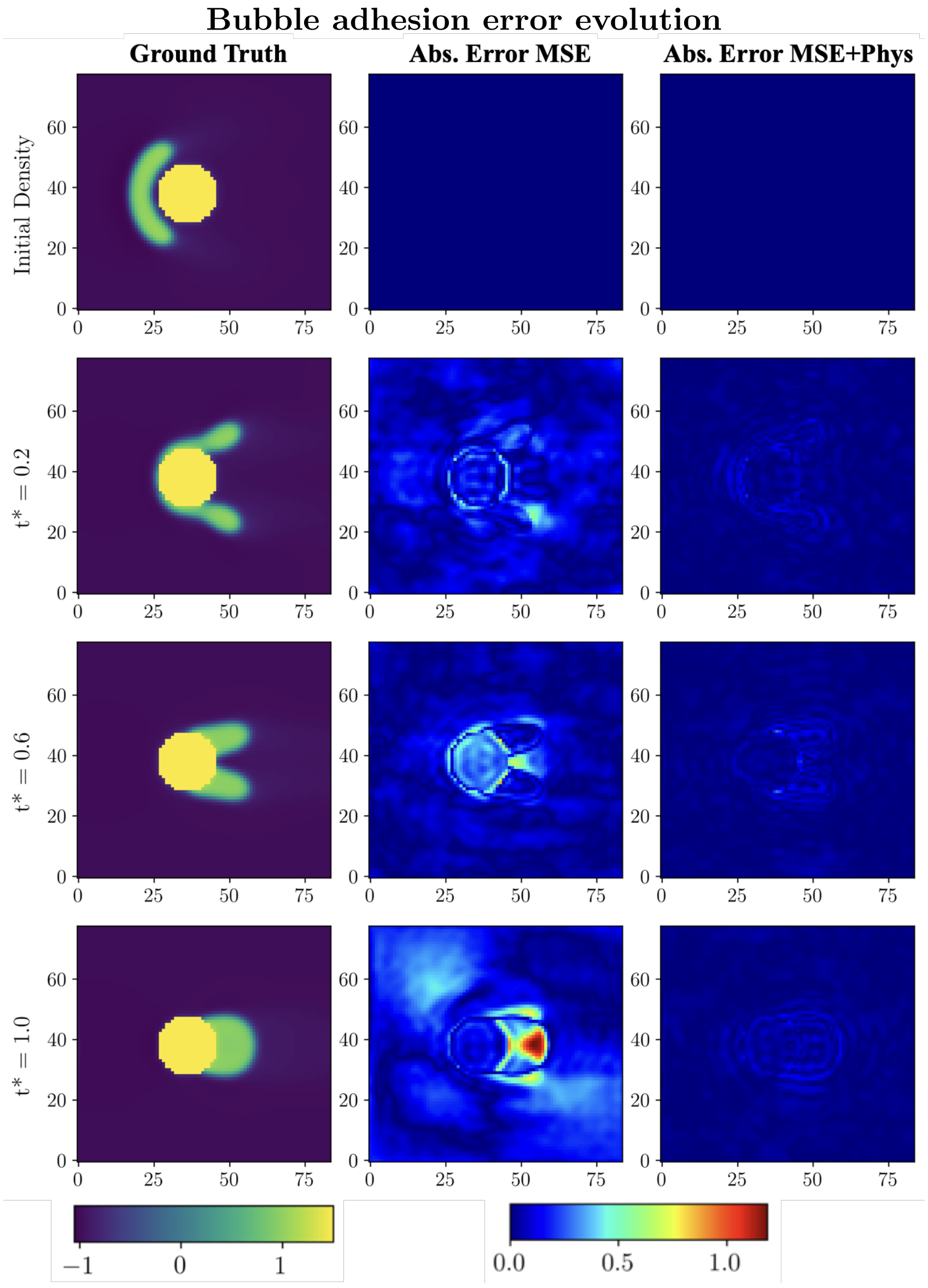

FIG. S10. Illustration of the error comparison between the MSE constraint and the MSE+Phys constraint. The left, middle, and right columns show the ground truth, the MSE-predicted absolute error, and the MSE+Phys-predicted absolute error, respectively. All errors are calculated via macroscopic results obtained via kinetic data. The first row shows the initial condition and absolute error. The right two columns display the absolute error evolution for the MSE and the MSE+Phys constraint.

trained $n = 10$ independent models for both the MSE and MSE+Phys constraints. The plot shows that the MSE loss is comparable to the MSE+Phys loss. However, in the macroscale quantity predictions shown in the main content (Fig. 2), we demonstrate that the MSE+Phys constraint outperforms the MSE constraint.

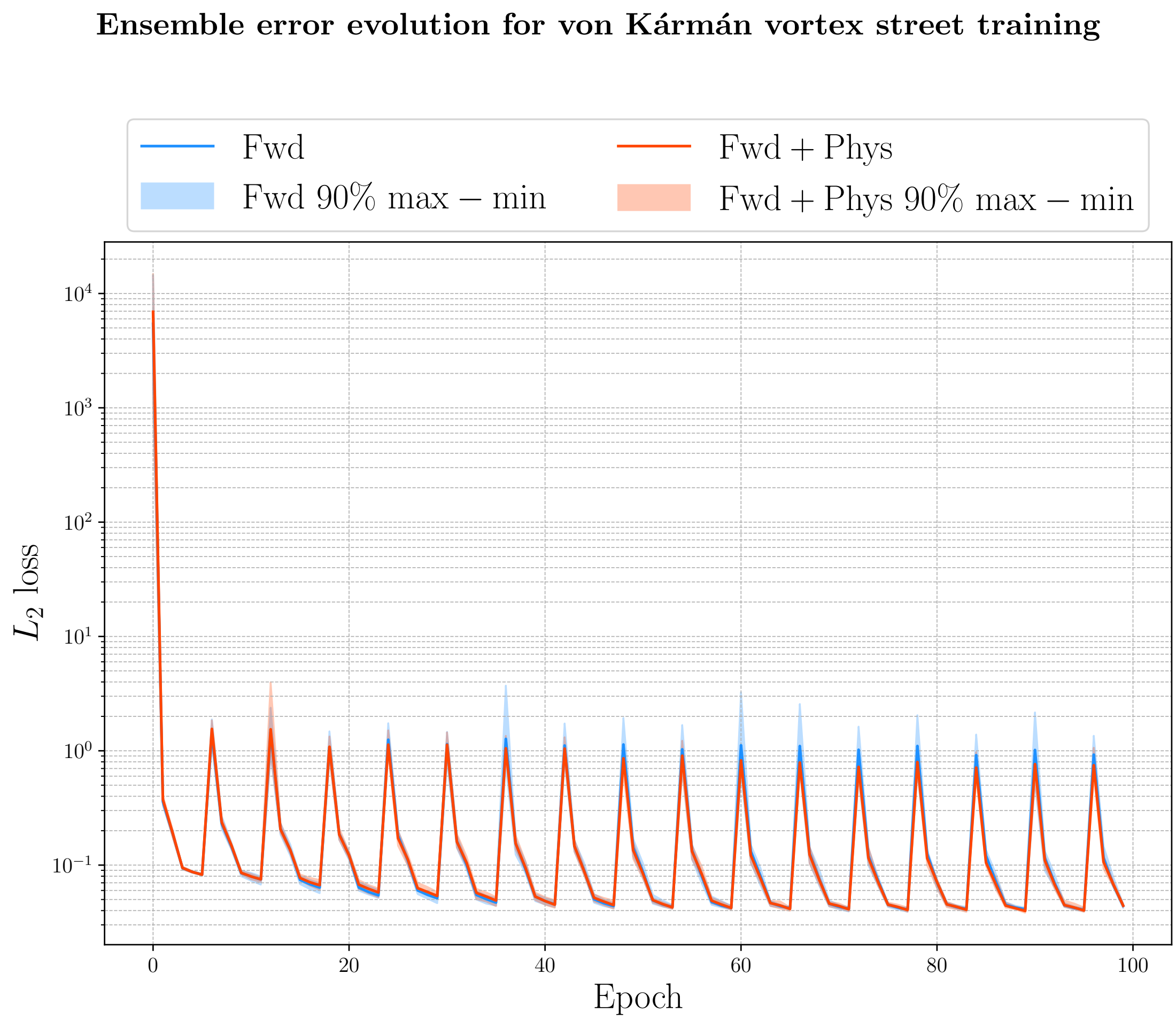

FIG. S11. Epoch evolution as a function of L_2-norm error for von Kármán vortex street. The solid blue and red lines represent the mean values of the forward loss (MSE loss) and the combined forward plus physical loss (MSE+Phys loss), respectively. The faded blue and red regions indicate the ranges corresponding to 90% of the maximum and minimum values for each respective constraint loss.

In Figure S12, the blue line represents the result of the MSE constraint. The red line represents the result of the MSE+Phys constraint. We observe that the median value of the MSE constraint for the liquid ligament breakup case is significantly higher compared to that of the MSE+Phys constraint case. In particular, the L_2-norm error remains relatively flat for the MSE constraint, suggesting that the training process may be trapped at a local minimum. A similar phenomenon is observed for the MSE+Phys case; however, the median value of the red line is systematically closer to the minimum value of the MSE+Phys constraint. This indicates that incorporating conservation laws and symmetry considerations leads to improved accuracy during training, ultimately resulting

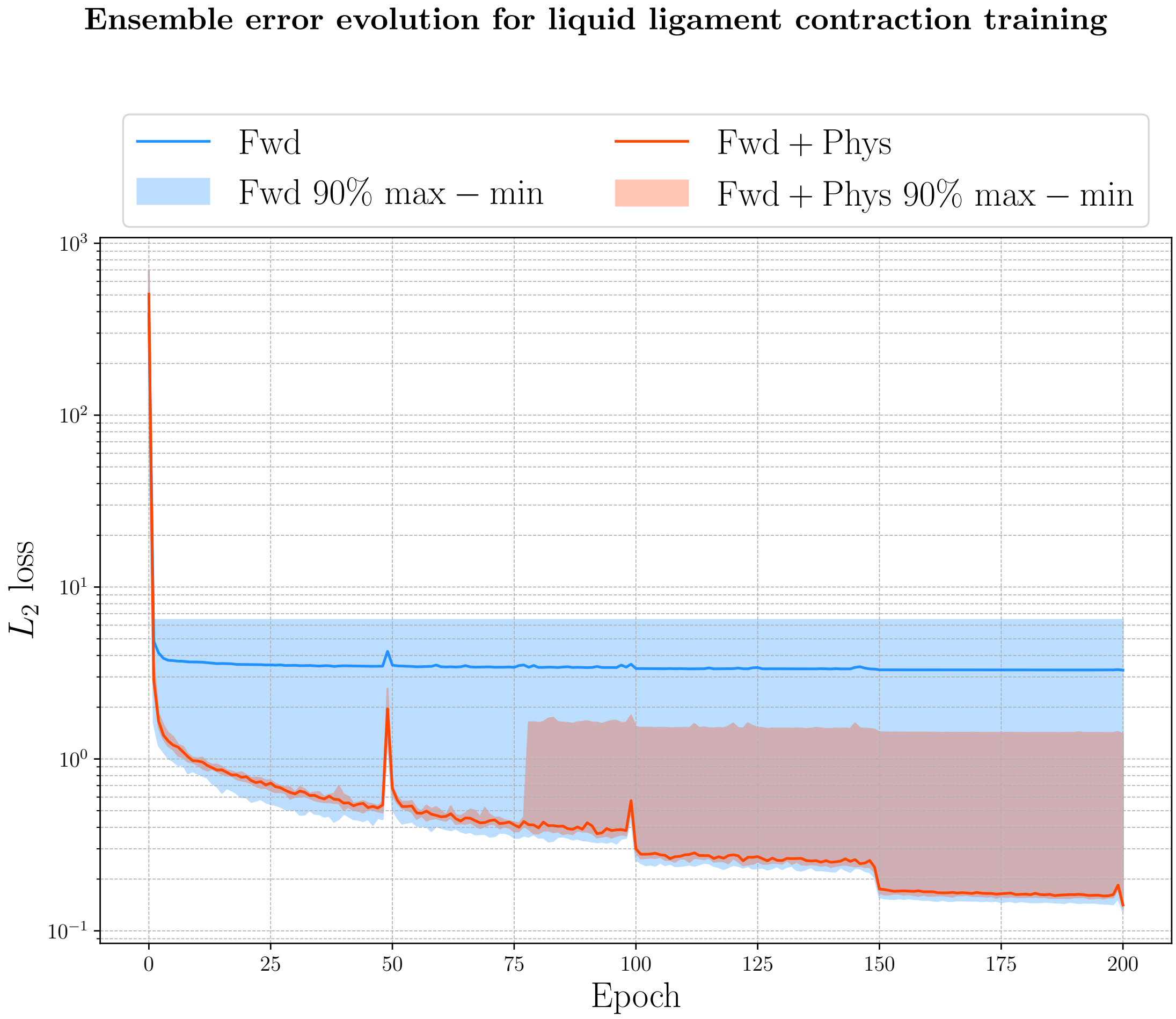

FIG. S12. Epoch evolution as a function of L_2-norm error for liquid ligament contraction. The solid blue and red lines represent the mean values of the forward loss (MSE loss) and the combined forward plus physical loss (MSE+Phys loss), respectively. The faded blue and red regions indicate the ranges corresponding to 90% of the maximum and minimum values for each respective constraint loss.

in a higher success rate.

In Figure S13, the blue line represents the result of the MSE constraint. The red line represents the result of the MSE+Phys constraint. We observe that the median value of the MSE constraint for the liquid ligament breakup case is significantly higher compared to that of the MSE+Phys constraint case. In particular, the L_2-norm error remains relatively flat for the MSE constraint, suggesting that the training process may be trapped at a local minimum. A similar phenomenon is observed for the MSE+Phys case; however, the median value of the red line is systematically closer to the minimum value of the MSE+Phys constraint. This indicates that incorporating conservation laws and

symmetry considerations leads to improved accuracy during training, ultimately resulting in a higher success rate.

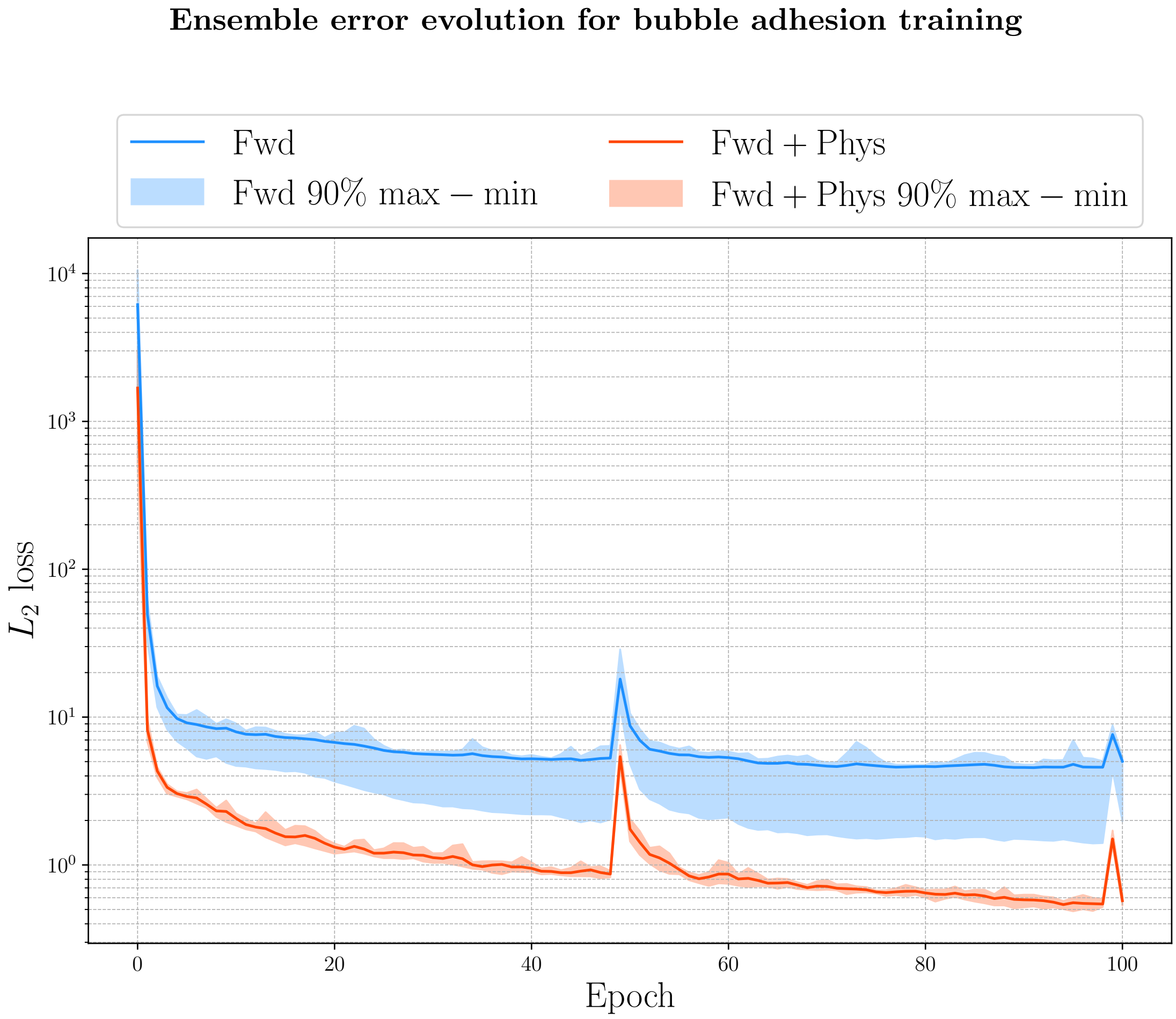

FIG. S13. Epoch evolution as a function of L_2-norm error for bubble adhesion study. The solid blue and red lines represent the mean values of the forward loss (MSE loss) and the combined forward plus physical loss (MSE+Phys loss). The faded blue and red regions indicate the ranges corresponding to 90% of the maximum and minimum values for each respective constraint loss.

S7. ABLATION STUDY: FNO RESULTS

To evaluate the generality and robustness of our proposed physics-informed framework, we extend its application to the Fourier neural operator. This section presents the primary experimental results obtained using FNO across the same physical scenarios explored in previous sections. By comparing baseline FNO models trained on MSE loss against their physics-informed counterparts (MSE+Phys), we aim to investigate whether

the incorporation of physical constraints yields consistent improvements in predictive accuracy across different architectures. We kept the number of FNO layers the same as in UNO and found that the trained FNO model requires 2 to 3 times more memory than UNO. Below we present all three different cases.

S7.1 Von Kármán vortex street

Figures S14 and S15 show the relative L_2 errors in density and velocity predictions, respectively, for the Kármán vortex street over dimensionless time t^*. In both cases, the model trained with the combined MSE+Phys loss achieves significantly lower prediction errors compared to the model trained with MSE alone. Moreover, the shaded regions, which represent 95% confidence intervals across $n = 10$ independently trained models, indicate that the MSE+Phys model consistently exhibits reduced variance across time. These results demonstrate that incorporating physics-based constraints can improve accuracy and enhance the stability and robustness of the learned dynamics.

S7.2 Liquid ligament breakup

Figure S16 demonstrates the relative L_2 density prediction errors for cylindrical ligament breakup, both MSE and MSE+phys constraints, within 6% of the relative error. Two models exhibit similar performance, with no significant differences observed. In both cases, the error increases as the ligament deforms to breakup periods.

S7.3 Bubble adhesion

We finally examine the bubble adhesion example. As illustrated, MSE+Phys consistently outperforms the pure MSE constraints, demonstrating the effectiveness of our proposed moment-matching and lattice-equivariant framework. The MSE results show error around 10% to 12% in terms of prediction of the phase field evolution. However, with the physics constraint, MSE+Phys results deduct the error down to 4% to 5%.

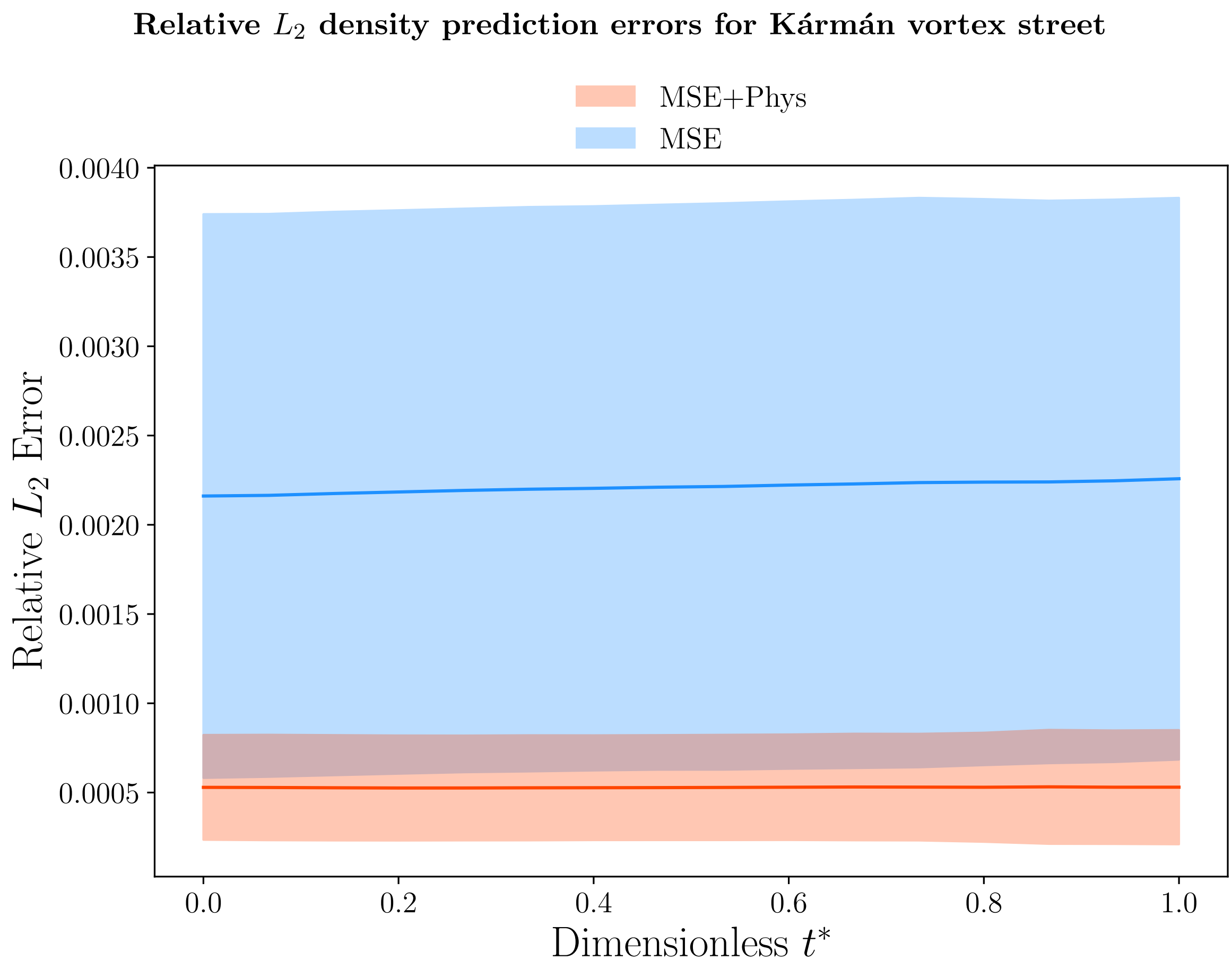

FIG. S14. The plot compares the baseline forward model (MSE loss) and the physics-augmented model (MSE+Phys loss). The x-axis represents dimensionless time steps ranging from 0 to 1, indicating the evolution of vortex dynamics. The y-axis quantifies the deviation in predicted density fields from the ground truth. The solid lines indicate the mean error over $n = 10$ independently trained models, and the shaded areas indicate 95% confidence intervals. Across all evaluated time steps, the MSE+Phys model consistently outperforms the standard MSE model by having lower loss and thinner error region.

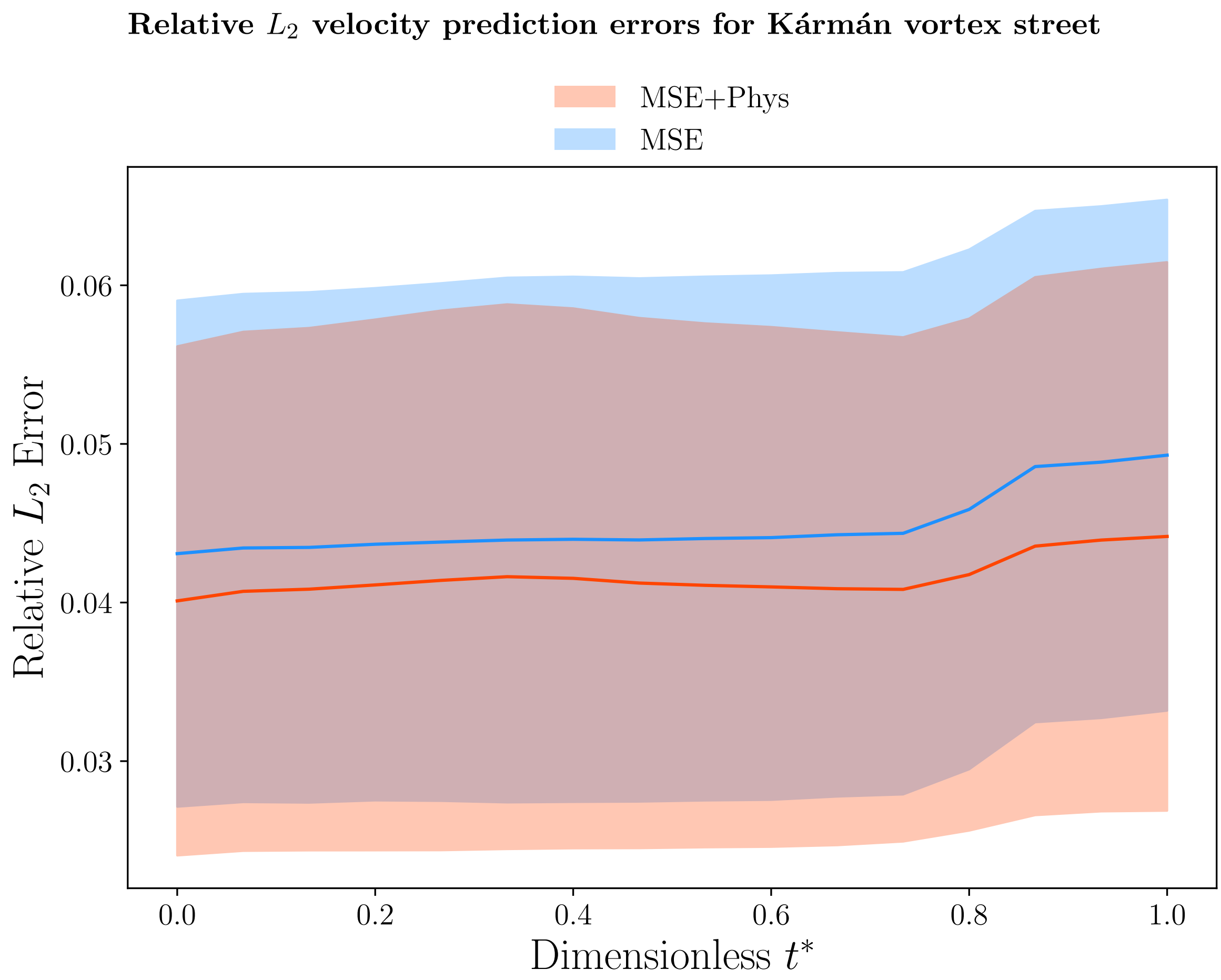

FIG. S15. The plot shows the temporal evolution of the relative L_2 error in velocity predictions, comparing a standard forward model (MSE) and a physics-augmented model (MSE+Phys). The solid lines indicate the mean error over $n = 10$ independently trained models, and the shaded regions represent 95% confidence intervals. The physics-informed model exhibits consistently lower prediction error, demonstrating improved generalization and physical coherence.

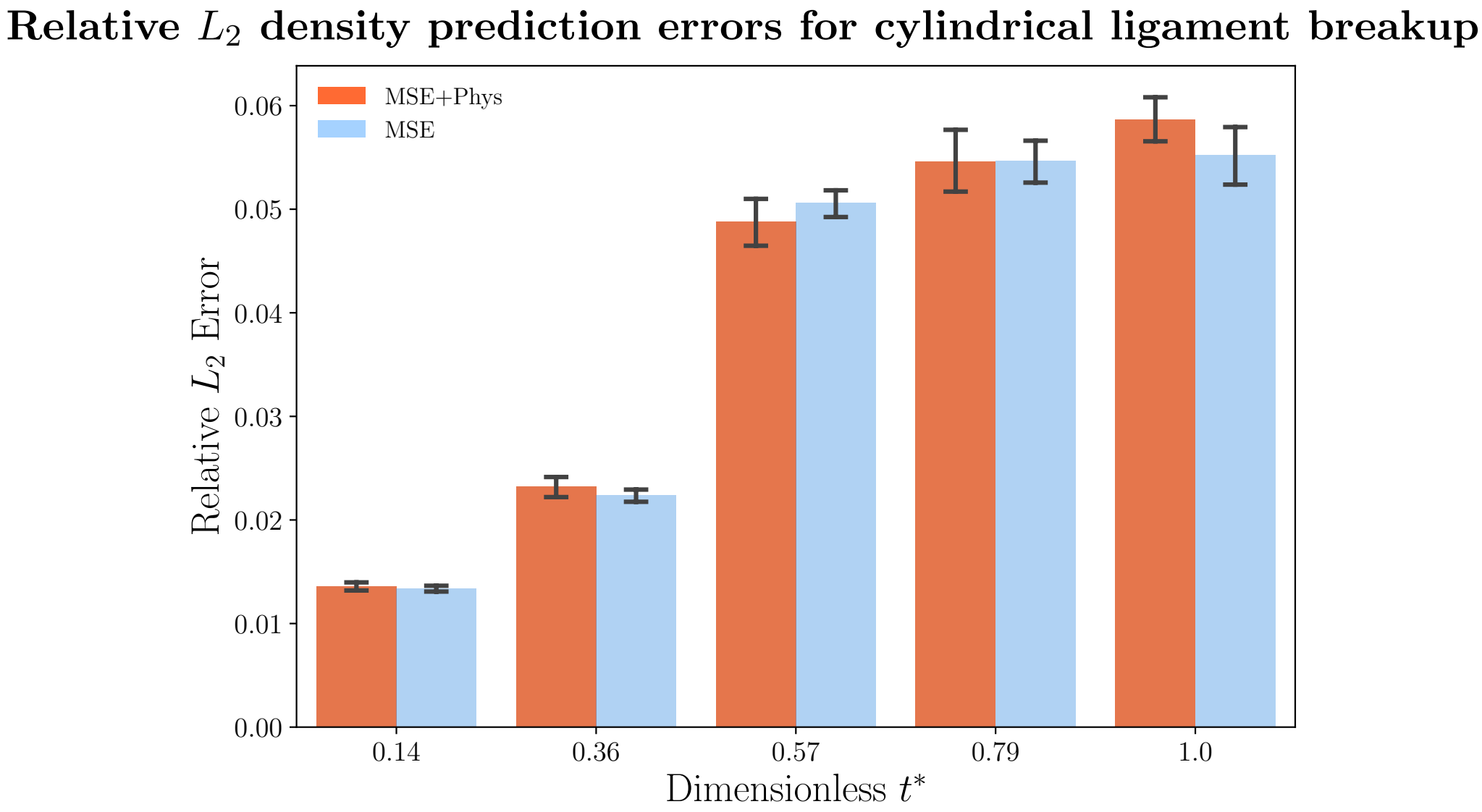

FIG. S16. The figure compares the relative L_2 error of density predictions obtained from the standard model (MSE) and its physics-informed counterpart (MSE+Phys). The x-axis represents dimensionless time $t^* = t/t_{\text{end}}$, capturing the evolution of the breakup process. The error bars denote 95% confidence intervals computed over $n = 10$ independently trained models.

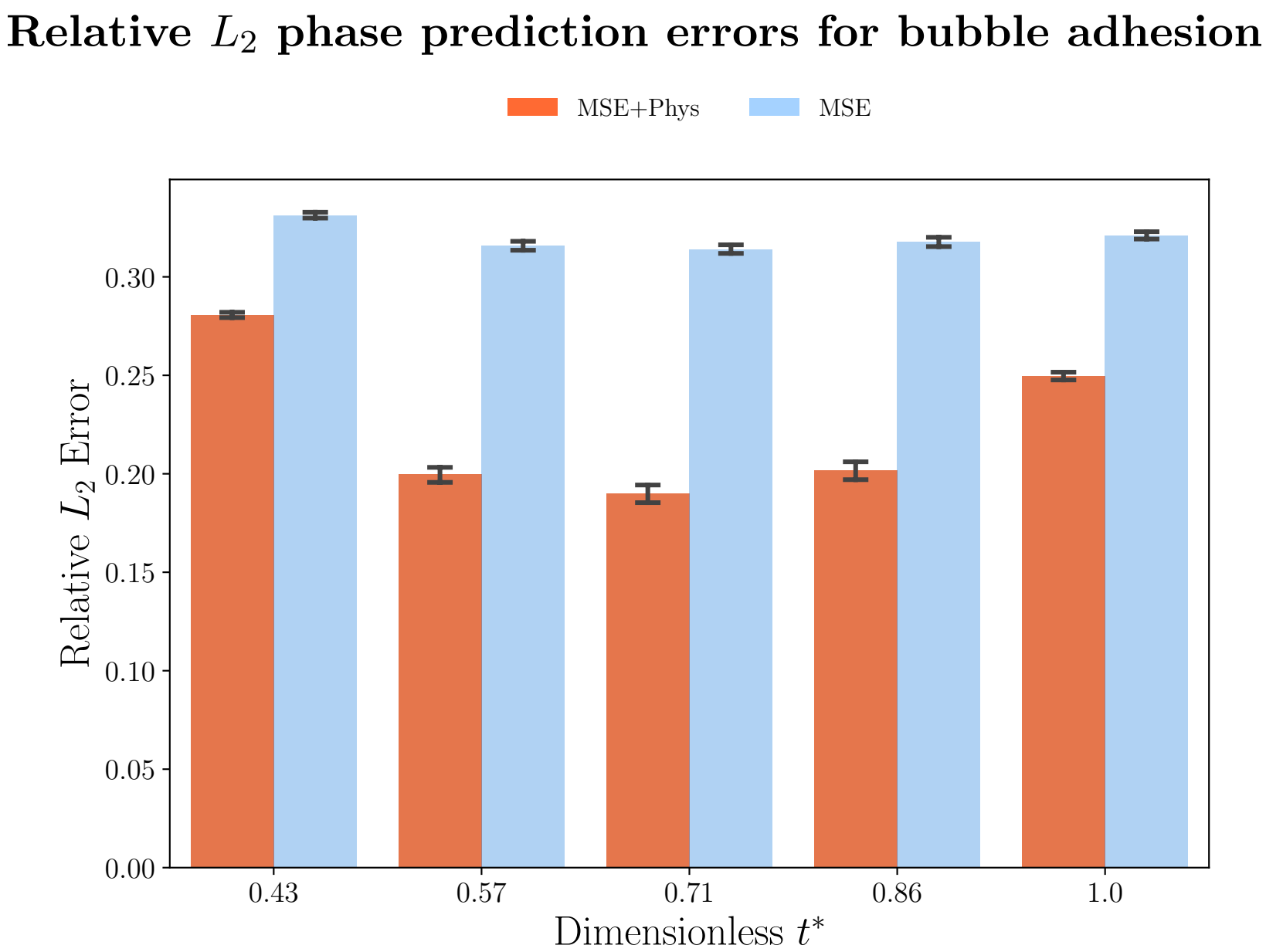

FIG. S17. The figure shows the relative L_2 error of phase predictions for the bubble adhesion task, comparing the standard model (MSE) with its physics-informed framework (MSE+Phys). The x-axis tracks the evolution of the bubble adhesion process. The error bars denote 95% confidence intervals computed over $n = 10$ independently trained models.

S8. ERROR METRICS

The error metrics which appear in Fig. S16 and Fig. S17 are measured by the absolute difference in macroscale quantities defined as:

$$E_{\text{abs}} = \left| y(\mathbf{x}) - \hat{y}(\mathbf{x}) \right| . \tag{S35}$$

The relative L2 loss in all cases is presented as follows:

$$L_2 = \frac{\sum_{i=1}^{n} (y_i - \hat{y}_i)^2}{\sum_{i=1}^{n} y_i^2} \tag{S36}$$

where n denotes the number of measured samples, y_i represents the actual target value for the i-th observation, and $\hat{y}_i$ is the corresponding predicted value.

$$\mathbf{M} = \begin{bmatrix}
1 & 1 & 1 & 1 & 1 & 1 & 1 & 1 & 1 & 1 & 1 & 1 & 1 & 1 & 1 & 1 & 1 & 1 & 1 \\
8 & 8 & -11 & 8 & 8 & 8 & -11 & 8 & -11 & -30 & -11 & 8 & -11 & 8 & 8 & 8 & -11 & 8 & 8 \\
1 & 1 & -4 & 1 & 1 & 1 & -4 & 1 & -4 & 12 & -4 & 1 & -4 & 1 & 1 & 1 & -4 & 1 & 1 \\
0 & -1 & 0 & 1 & 0 & -1 & 0 & 1 & -1 & 0 & 1 & -1 & 0 & 1 & 0 & -1 & 0 & 1 & 0 \\
0 & -1 & 0 & 1 & 0 & -1 & 0 & 1 & 4 & 0 & -4 & -1 & 0 & 1 & 0 & -1 & 0 & 1 & 0 \\
-1 & 0 & 0 & 0 & 1 & -1 & -1 & -1 & 0 & 0 & 0 & 1 & 1 & 1 & -1 & 0 & 0 & 0 & 1 \\
-1 & 0 & 0 & 0 & 1 & -1 & 4 & -1 & 0 & 0 & 0 & 1 & -4 & 1 & -1 & 0 & 0 & 0 & 1 \\
-1 & -1 & -1 & -1 & -1 & 0 & 0 & 0 & 0 & 0 & 0 & 0 & 0 & 0 & 1 & 1 & 1 & 1 & 1 \\
-1 & -1 & 4 & -1 & -1 & 0 & 0 & 0 & 0 & 0 & 0 & 0 & 0 & 0 & 1 & 1 & -4 & 1 & 1 \\
-2 & 1 & -1 & 1 & -2 & 1 & -1 & 1 & 2 & 0 & 2 & 1 & -1 & 1 & -2 & 1 & -1 & 1 & -2 \\
-2 & 1 & 2 & 1 & -2 & 1 & 2 & 1 & -4 & 0 & -4 & 1 & 2 & 1 & -2 & 1 & 2 & 1 & -2 \\
0 & -1 & -1 & -1 & 0 & 1 & 1 & 1 & 0 & 0 & 0 & 1 & 1 & 1 & 0 & -1 & -1 & -1 & 0 \\
0 & -1 & 2 & -1 & 0 & 1 & -2 & 1 & 0 & 0 & 0 & 1 & -2 & 1 & 0 & -1 & 2 & -1 & 0 \\
0 & 0 & 0 & 0 & 0 & 1 & 0 & -1 & 0 & 0 & 0 & -1 & 0 & 1 & 0 & 0 & 0 & 0 & 0 \\
1 & 0 & 0 & 0 & -1 & 0 & 0 & 0 & 0 & 0 & 0 & 0 & 0 & 0 & -1 & 0 & 0 & 0 & 1 \\
0 & 1 & 0 & -1 & 0 & 0 & 0 & 0 & 0 & 0 & 0 & 0 & 0 & 0 & 0 & -1 & 0 & 1 & 0 \\
0 & 1 & 0 & -1 & 0 & -1 & 0 & 1 & 0 & 0 & 0 & -1 & 0 & 1 & 0 & 1 & 0 & -1 & 0 \\
-1 & 0 & 0 & 0 & 1 & 1 & 0 & 1 & 0 & 0 & 0 & -1 & 0 & -1 & -1 & 0 & 0 & 0 & 1 \\
1 & -1 & 0 & -1 & 1 & 0 & 0 & 0 & 0 & 0 & 0 & 0 & 0 & 0 & -1 & 1 & 0 & 1 & -1
\end{bmatrix} . \tag{S37}$$

[1] D. d'Humières, Multiple–relaxation–time lattice Boltzmann models in three dimensions, Philosophical Transactions of the Royal Society of London. Series A: Mathematical, Physical and Engineering Sciences **360**, 437 (2002).

[2] J. Smagorinsky, General circulation experiments with the primitive equations: I. the basic experiment, Monthly Weather Review **91**, 99 (1963).

[3] S. Hou, J. Sterling, S. Chen, and G. Doolen, A lattice Boltzmann subgrid model for high Reynolds number flows, arXiv preprint comp-gas/9401004 (1994).

[4] Y. Koda and F.-S. Lien, The lattice Boltzmann method implemented on the gpu to simulate the turbulent flow over a square cylinder confined in a channel, Flow, Turbulence and Combustion **94**, 495 (2015).

[5] X. Xue, H.-D. Yao, and L. Davidson, Synthetic turbulence generator for lattice Boltzmann method at the interface between RANS and LES, Physics of Fluids **34**, 055118 (2022).

[6] X. Shan and H. Chen, Lattice Boltzmann model for simulating flows with multiple phases and components, Phys. Rev. E **47**, 1815 (1993).

[7] X. Shan and H. Chen, Simulation of nonideal gases and liquid-gas phase transition by the lattice Boltzmann equation, Phys. Rev. E **49**, 2941 (1994).

[8] J. Zhang, Lattice Boltzmann method for microfluidics: models and applications, Microfluid. Nanofluid. **10**, 1 (2011).

[9] M. Sega, M. Sbragaglia, S. S. Kantorovich, and A. O. Ivanovd, Mesoscale structures at complex fluid-fluid interfaces: a novel lattice Boltzmann/molecular dynamics coupling, Soft Matter **9**, 10092 (2013).

[10] X. Xue, L. Biferale, M. Sbragaglia, and F. Toschi, A lattice Boltzmann study on brownian diffusion and friction of a particle in a confined multicomponent fluid, Journal of Computational Science **47**, 101113 (2020).

[11] G. Ortali, A. Gabbana, I. Atmodimedjo, and A. Corbetta, Enhancing lattice kinetic schemes for fluid dynamics with Lattice-Equivariant Neural Networks, AIAA Journal **63**, 716 (2025).

[12] D. Hendrycks and K. Gimpel, Gaussian error linear units (GELU), arXiv preprint arXiv:1606.08415 (2016).

[13] A. Friedman, *Generalized Functions and Partial Differential Equations* (Courier Corpora-

tion, 2005).

[14] M. A. Rahman, Z. E. Ross, and K. Azizzadenesheli, U-no: U-shaped neural operators, arXiv preprint arXiv:2204.11127 (2022).

[15] Z. Li, N. Kovachki, K. Azizzadenesheli, B. Liu, K. Bhattacharya, A. Stuart, and A. Anandkumar, Fourier neural operator for parametric partial differential equations, arXiv preprint arXiv:2010.08895 (2020).

[16] X. Xue, M. Sbragaglia, L. Biferale, and F. Toschi, Effects of thermal fluctuations in the fragmentation of a nanoligament, Physical Review E **98**, 012802 (2018).

[17] D. Chiappini, M. Sbragaglia, X. Xue, and G. Falcucci, Hydrodynamic behavior of the pseudopotential lattice Boltzmann method for interfacial flows, Physical Review E **99**, 053305 (2019).

[18] S. Succi, *The lattice Boltzmann equation: for fluid dynamics and beyond* (Oxford University Press, 2001).

[19] T. Krüger, H. Kusumaatmaja, A. Kuzmin, O. Shardt, G. Silva, and E. M. Viggen, *The Lattice Boltzmann Method*, Graduate Texts in Physics (Springer International Publishing, Cham, 2017).

**Applications**	**Lattice Model**	**Lattice Model Illustration**	**Collision Kernel**	**Hyperparameter for loss function**	**Equivariant representation of macroscopic equations**
Kármán vortex street	D3Q19	$x \in [14:2:34], \alpha \in [0:0.04:2)$	Smagorinsky Large-eddy simulations collision kernel	$\lambda_{\text{MSE}} = 0.5$, $\lambda_1 = 10$, $\lambda_2 = 1$,$\lambda_3 = 1$	Navier-Stokes equations
Liquid ligament breakup	D3Q19	$\mathbf{x}_c \in [140:1:170] \times R_c[10:1:20]$	Multiple-relaxation-time collision kernel with Shan-Chen forcing	$\lambda_{\text{MSE}} = 0.5$,$\lambda_1 = 1$, $\lambda_2 = 0$, $\lambda_3 = 1$	Navier-Stokes equations for multiphase flows
Bubble adhesion in phase space	D2Q9	$v \in [1.5:0.1:2.5], t \in [0:1:100]$	Bhatnagar-Gross-Krook (BGK) collision kernel	$\lambda_{\text{MSE}} = 0.5$,$\lambda_1 = 1$, $\lambda_2 = 0$,$\lambda_3 = 1$	Cahn-Hilliard equation coupled with Navier-Stokes equations

TABLE S1. Table illustrating the selection of applications and corresponding hyperparameters for the MSE+Phys loss function in this work, highlighting their relevance to multiple physical phenomena, the corresponding lattice models, and equations of motion at the macroscale.

**Examples**	Domain size	$T_{\mathbf{LB}}^{\mathbf{total}}$	Effective Domain size	$T_{\mathbf{LB}}^{\mathbf{eff}}$
Kármán vortex street LR (c++)	$110 \times 80 \times 3$ LU	17.24 ± 1.8s	80×80 LU	4.18 ± 0.5s
Liquid ligament breakup (c++)	$48 \times 128 \times 48$ LU	14.88 ± 1.5s	128×48 LU	0.31 ± 0.03s
Bubble adhesion in phase space	100×300 LU	104.34 ± 3.4s	84×78 LU	22.79 ± 0.74s

TABLE S2. Comparison of full-domain and effective-domain LB simulation runtimes for the three benchmark examples. All LB simulations were executed in single precision (FP32) on the Intel(R) Xeon(R) Gold 6254 CPU and repeated ten times; reported values denote the mean ± standard deviation. $T_{\mathrm{LB}}^{\mathrm{total}}$ corresponds to the runtime of the full-domain LB simulation, while $T_{\mathrm{LB}}^{\mathrm{eff}}$ denotes the runtime when restricting computation to the effective domain. Domain sizes are reported in lattice units (LU).
