# Supplementary material for: Fast-forward prediction of lattice Boltzmann dynamics with physics-informed neural operators
Source: Nat Commun. 2026 Jul 29;17:9243. doi: 10.1038/s41467-026-75730-1 (PMC13526859; doi:10.1038/s41467-026-75730-1)
Supplement: Supplementary file 2 [file 41467_2026_75730_MOESM2_ESM.pdf]

nature portfolio

Peer Review File

Fast-forward prediction of lattice Boltzmann dynamics with physics-informed neural operators

Corresponding Author: Professor Peter Coveney

This file contains all reviewer reports in order by version, followed by all author rebuttals in order by version.

Attachments originally included by the reviewers as part of their assessment can be found at the end of this file.

Version 0:

Reviewer comments:

Reviewer #1

(Remarks to the Author)
The paper introduces a machine learning framework to create a surrogate model for lattice Boltzmann (LB) simulations. The article argues that traditional LBE solvers are computationally expensive because they require fine time-stepping to resolve local particle collisions and streaming. The proposed AI approach uses a neural network to fast-forward the simulation by directly predicting the evolution of single-particle distribution functions (PDFs) over large time horizons, bypassing the need to explicitly compute the collision kernel at each step.

The approach uses a Physics-Informed Neural Operator (PINO), specifically built on the U-shaped Neural Operator (UNO) and lears a global operator mapping initial PDF fields to future ones, while incorporating constraints of rotational and reflection invariance to ensure consistency across scales. For added physical consistency, it incorporates macroscopic variable (moments of the PDFs) to the loss function. In this way, it makes sure to train the PINO for the evolution of both the kinetic and macroscopic variables.

Three examples are shown: 2D von Karman vortex street (with a D3Q19 LBM), liquid-ligament breakup (with Shan/Chen), and bubble adhesion (with a phase-field approach), where the learned operator replaces multiple LBM timesteps with one operator call. The authors claim that the approach is predictive, cross-collision and cross-physics, discretization-invariant, and two to three orders of magnitude faster than conventional LBM. As desribed below, it is however not clear that the presented data fully supports these claims.

The topic is timely and aligns with the growing interest in scientific machine learning. Playing with a neural operator advancing with large time steps, instead of applying PINNS at collision-streaming level, deserves attention and opens a field that has to my knowledge not been adressed so far in lattice Boltzmann.

However, while the manuscript demonstrates that the approach "works", it does not provide any motivation for applying the PINO framework to PDFs instead of directly learning the macroscopic variables (as all presented cases are classical CFD problems described as solutions of a macroscopic eqution). In such cases, the use of a LBM approach in place of classical CFD is usually motivated by the computational efficiency of the scheme, or the simplicity of boundary modeling, or other similar arguments, none of which seem to be valid here. The lack of comparison of a PDF vs macroscopic-variable approach, and the lack of comparison of the results with the existing literature of PINO use, makes it difficult to find a motivation to the fundamental premises of the article. Although many parts of the manuscript suggest an advantage to the proposed approach, this implicit claim is unsupported throughout the work

Another fundamental issue stems from the reference simulations themselves, which are unvalidated in the article and raise questions about their quality and validity. I therefore believe that the paper requires a major rewrite with a clearer scope and a stronger link between specific claims and their validation.

To start with the issue of unvalidated simulations, the authors present three canonical benchmark cases for which reliable reference data are available. However, they do not validate their own lattice Boltzmann results against any known result. In particular, the von Karman vortex street example displays clear signs of numerical instability near the cylinder, as well as

spurious oscillations originating from the inlet. Given the simplicity of this test case, these issues raise serious concerns about whether a state-of-the-art numerical setup was employed. The authors should have considered for instance using a modern LBM collision model, such as the cumulant approach, to mitigate these instabilities. As a consequence of these deficiencies, the neural operator is trained on data that contain evident numerical errors and, concerningly, reproduces the same incorrect instability pattern with high accuracy. This outcome weakens the claim that the framework is physics-informed and may even, to the contrary, lead to the impression that it primarily reproduces patterns present in the training data.

Part of the difficulty likely stems from the small computational domains, which appear to have been chosen to satisfy the memory constraints of the machine-learning framework. While such limitations are understandable, the manuscript should include a clear and quantitative discussion of the memory restrictions, the corresponding design choices, and the implications for the fidelity and interpretability of the simulations performed under these conditions.

Also, while the novelty of the manuscript appears to rest on applying the operator to probability distribution functions rather than to the velocity field, as was done in previous non-LBM studies, the purpose and potential advantage of this choice is not well stated. This lack of clarity contrasts with the strong rhetorical emphasis placed on the "kinetic" aspect throughout the article, illustrated by repeated expressions such as "underlying kinetic system", "kinetic super-resolution", "kinetic dataset", "kinetic-focused ML", "kinetic constraints", "kinetic simulations", "underlying kinetic physics", "kinetic-based method", "kinetic data-driven framework", and "kinetic regimes". In reality, the proposed approach appears to have an inherent disadvantage, as it requires training the operator on 19 fields instead of three (velocity) or four (velocity and phase field). Interestingly, the authors report their best results when including both a term for the PDFs and a term for the velocity and density into the loss function. This outcome suggests that the most effective formulation is not purely "kinetic" but rather hybrid. It would therefore be natural to question whether the use of PDFs is necessary at all for the neural operator, or whether comparable performance could be obtained with a loss function based solely on macroscopic variables (computed through the LBM or any CFD method producing equivalent results). Including such an investigation would considerably strengthen the paper and help articulate its central message.

The article further contains unsupported claims :

- The operator is claimed to be faster than plain LBM, but this comparison is flawed due to mismatched hardware: the LBM was run on a CPU with poor performance (an Intel Core-i7) while the ML framework runs on a fast NVIDIA 4090 GPU. As it is common nowadays to run LB simulations on GPU, the LB should be run on the same GPU fs the ML framework or fair comparison. Furthermore, the performance figures should be stated clearly and should be verifiable. This is not possible with the currenlty provided materials (the execution time is reported but the corresponding domain size and number of iterations are not clearly stated). For this statement, as well as for other reported simulation results, the documentation must be more systematic so the reader can make sense out of the reported data.

- The paper state that the framework is predictive and claims cross-collision and cross-physics applicability, but the meaning of these statements is unclear. To be clear, training and inference consistently use the same collision models and physics throughout the three presented examples. If I didn't fundamentally misread the article, no case is presented in which a model trained on one type of physics, or on one observed phenomenon, is used to exhibit different physics or behavior. In the case of the von Karman street, the model samples a period of the uniform oscillation with different windows and then reproduces the same oscillation it was trained on. In the two multiphase examples, some parameters (initial placement) were varied to sample multiple configurations in training and infer a result from another configuration. To clarify, it could be good to show how the predicted result differs from one of the training runs with similar initial condition; as it stands, the article however provides no data to suggest cross-physics applicability.

- The paper claims the model achieves super-resolution, a term which I believe would suggest the ability to recover unresolved subgrid structure from coarse data. But the article does not offer any evidence of this. It certainly is interesting to note that the UNO operator used in the case claimed to exhibit super-resolution is resolution invariant due to its internal data representation. But the provided analysis does not show that the produced hires result is in any way better than an interpolated copy of a low-resolution output. It would be difficult to exhibit actual superresolution with the chosen test cases, which are represented by laminar and smooth fields. Only a quantitative comparison with reference results of the test case could show if the operator can get any actual improvement from its application to finer resolved data.

I would finaly like to comment on the three following points:

- On page 3, the authors state: "a rigorous derivation of continuum equations from underlying particle dynamics—a challenge that has recently seen major progress through the work of Deng et al". A rigorous derivation of continuum euqations from underlying paritcle dynamics is a topic that has been throughly investigated in more than a century. It is delicate to claim "major progress" in this field by referencing a non peer-reviewed manuscript.

- In the abstract, and later in the text, the authors state that the LBM is limited to small time-steps "due to strict time-step restrictions imposed by collision kernels". This statement is difficult to understand. It would appear more precise to attribute the small time-step limitation to explicit time-stepping constraints which are also present as CFL-like condition in other explicit time-stepping schemes.

(Remarks on code availability)

Reviewer #2

(Remarks to the Author)
See attached review in pdf file.

(Remarks on code availability)

Reviewer #3

(Remarks to the Author)
In the manuscript titled “Fast-Forward Lattice Boltzmann: Learning Kinetic Behaviour with Physics-Informed Neural Operators”, the authors presented a physics-informed neural operator framework for the lattice Boltzmann equation, a novel data-driven approach for modelling fluid flows. Three cases were tested which covered both single-phase and multiphase flows using the large-eddy simulation model, Shan-Chen model and phase-field model. This represents wide range of interests. To improve the work, some specific suggestions are listed as below.

1.In S3, the LB simulations with the C++ code were run on the 1.2 GHz Quad-Core Intel Core I7 but the machine learning inference part was done on a NVIDIA 4090 24GB card. That is, the comparisons were based on different hardware. It is unclear if the LB simulations were using single-core or multiple cores. But it is not unusual that, compared with a single CPU core, the LB simulations could be run hundreds of times faster on a GPU card like 4090. Therefore, it is suggested to conduct comparisons using the same hardware so that more insights into the performance could be obtained. It will be very useful if the memory consumption could be compared as well.

2.Which precision was used for the float number in the LB simulations and the machine learning part?

3.How was machine learning framework implemented? It is suggested to introduce the software stack used, list some pseudo codes for key steps, and include a flowchart for the workflow in supplementary information.

4.How many resources were used for the training? These could include the time used for the training and the actual memory consumed on A100.

5.It appears that acceleration ratio for the liquid ligament breakup case is significantly different from the other two 2D cases. What could be the reason?

6.It will be great to have a discussion on how the “traditional” LB simulations and the machine learning counterpart could work together in the future. Apart from the potentially faster predictions, are there any other benefits by using the machine learning framework?

7.Apart from the UNO and FNO used in this work, is there other neural operator for choice?

8.A discussion on how to choose the hyperparameter for the loss functon(i.e., those listed in Table A1) will be very helpful. What will be the impact on the results if another set of hyperparameters are chosen?

9.At Page 16 of the supplementary information, “Table VI to Table VIII” was cited which probably should be Table S6 To Table S8. Table S4 and Table S5 appears not cited in the text.

(Remarks on code availability)

Version 1:

Reviewer comments:

Reviewer #2

(Remarks to the Author)
See attached review document.

(Remarks on code availability)

Reviewer #3

(Remarks to the Author)
The authors have satisfactorily addressed all my comments in the revised manuscript. In particular, the revised work

demonstrates that the ML predictions for test cases are faster than the traditional LBM modeling approach when using the same hardware. This is promising, and I therefore recommend the manuscript for publication.

(Remarks on code availability)

Version 2:

Reviewer comments:

Reviewer #2

(Remarks to the Author)
I am now satisfied by the authors' responses to my concerns and changes to the paper that address them. In particular, I appreciate the further discussion and the addition of the training cost information.

I provide some advice/suggestions below which the authors are welcome to include in this paper, but I will not hold this up with further iteration.

As I am sure you are aware, the term 'kinetic' is a fuzzily defined term in many fields, and can be interpreted in different ways by many readers, which can lead to definitional mismatches similar to what I was arguing in my review. In particular, researchers in some fields think of the Navier-Stokes equations, or at least the diffusion/viscosity/conductivity transport coefficients as 'kinetic effects', as in some derivations e.g. Chapman-Enskog these can be calculated from an underlying distribution properties, which are is approximated by the NSE solution. Similarly, the BGK collision operator indirectly assumes that your system will remain reasonably close to Maxwellian by some metric. I've seen this in particular in the fusion modeling community, but I see it occasionally in other hydrodynamics-focused application areas. So while by one definition, i.e., that you are solving a phase-space equation for the distribution function means it is kinetic, readers may assume that you are 'really' solving the NSE in another way, even if they know better (myself included).

For my implicit time-stepping question, I stand by some of my complaints about LB vs the other models/numerical formulations, but you raised an excellent point in your response that is worth foregrounding to stave off similar criticisms in future, namely, that due to its explicit timestepping nature and relatively simple quadratures, LB is *much* more efficient on a timestep to timestep basis than implicit or semi-implicit solves used in other kinetic models. Other fields may be more sensitive to stability concerns (e.g. including absorption/re-emission physics in photons) which push them to implicit methods, whereas you note other multiphysics effects (e.g. turbulence closures) which assume locality here. I'd still be curious to see a semi-implicit LB model someday but as you note that is beyond the scope of this paper.

(Remarks on code availability)

Open Access This Peer Review File is licensed under a Creative Commons Attribution 4.0 International License, which permits use, sharing, adaptation, distribution and reproduction in any medium or format, as long as you give appropriate

credit to the original author(s) and the source, provide a link to the Creative Commons license, and indicate if changes were made.
In cases where reviewers are anonymous, credit should be given to 'Anonymous Referee' and the source.
The images or other third party material in this Peer Review File are included in the article's Creative Commons license, unless indicated otherwise in a credit line to the material. If material is not included in the article's Creative Commons license and your intended use is not permitted by statutory regulation or exceeds the permitted use, you will need to obtain permission directly from the copyright holder.
To view a copy of this license, visit https://creativecommons.org/licenses/by/4.0/

Response to Reviewer #1

Response to Reviewer #1

We thank the reviewer for the detailed and thoughtful comments. Below we list the reviewer's points (R1.x) followed by our responses. Reviewer comments are reproduced verbatim for clarity. The blue text indicates adjustments in our manuscripts.

Major Comments

R1.1 Motivation for applying PINO to PDFs rather than macroscopic variables. *The manuscript does not provide a motivation for applying the PINO framework to PDFs instead of directly learning the macroscopic variables. All presented cases are classical CFD problems described through macroscopic equations. The use of LBM in place of classical CFD is typically justified by its computational efficiency or boundary simplicity, neither of which are relevant here. The lack of comparison between a PDF-based vs macroscopic-variable-based operator, and the lack of comparison with existing PINO literature, make it difficult to motivate the core premise of the article.*

Response to R1.1 We thank the reviewer for raising this important point regarding the motivation for learning the particle distribution functions (PDFs) rather than only the macroscopic variables. However, our intention in this paper is not to learn a macroscopic CFD neural operator, but specifically to accelerate the kinetic time advancement intrinsic to the lattice Boltzmann method (LBM). Predicting PDFs directly is motivated by three considerations.

First, the LBM evolution is fundamentally defined at the kinetic level through the collision and streaming of discrete PDFs, from which macroscopic quantities are obtained as moments. Learning the PDF evolution allows us to emulate the LBM time advancement without requiring the network to rediscover equilibrium closures or non-equilibrium moments that are already encoded in the LBM formulation. This construction preserves compatibility with any macroscopic post-processing used by LBM and is far richer, as it retains much more information.

Second, many LBM-based multiphysics models, including those used in our Shan-Chen and phase-field examples, depend explicitly on non-equilibrium PDFs to compute interfacial stresses, forcing terms, and phase coupling. These quantities cannot be reconstructed from macroscopic fields alone. A macroscopic model requires an additional closure or reconstruction step, whereas a PDF-based model naturally retains the information needed for these applications. If high-order macroscopic moments were retained, it would be impossible to reconstruct the non-equilibrium component.

Third, a PDF-based neural operator inherits the discretization independence of LBM. Since the operator acts on the discrete velocity components rather than on grid-dependent macroscopic fields, it can be applied at different spatial resolutions or macroscopic reconstructions without retraining. This aligns with our goal of designing a drop-in surrogate for the LBM kinetic update rather than a new macroscopic CFD method.

Importantly, as discussed in Shan's work and related to Grad's approach, LBM can represent kinetic effects that go beyond PDE-governed macroscopic dynamics. In such settings, the macroscopic fields alone do not contain sufficient information to uniquely reconstruct the full set of PDFs. If the macroscopic moments are absent or incomplete in the training data, any reconstruction step becomes ill-posed and introduces significant errors in the non-equilibrium components, often causing the LBM solver to become unstable. Even when the data are generated from a pure LBM simulation, the reconstruction of PDFs from macroscopic variables produces incorrect non-equilibrium contributions, leading to the propagation of systematic errors during time advancement.

Regarding the hybrid loss formulation: while the kinetic loss enforces consistency at the PDF level, the macroscopic loss promotes global-local mapping and stability. The combined loss reflects the multiscale nature of the underlying kinetic system. This observation does not contradict the motivation for using PDFs; rather, it confirms that both scales are important for robust LBM acceleration.

To clarify these points, we have revised the Introduction to state more explicitly the motivation and scope of our method.

In the revised manuscript (Introduction), we have clarified the motivation for predicting PDFs rather than only macroscopic fields as follows (Introduction, Page 4):

"Our goal is to construct a surrogate for the kinetic time advancement that is intrinsic to the lattice Boltzmann equation. Predicting the discrete single particle distribution functions preserves the full non-equilibrium content of the kinetic description. Importantly, as highlighted in Shan's work[35], the kinetic representation can encode physical effects beyond those captured by macroscopic PDEs. When only low-order macroscopic moments are available, reconstructing the PDFs becomes a non-unique and often ill-posed inverse problem. The macroscopic fields do not contain sufficient information to uniquely determine the non-equilibrium components of the PDFs, and any missing or incomplete moment information leads to reconstruction errors. These errors directly contaminate the non-equilibrium stresses that drive LBE dynamics, and even small inaccuracies can cause numerical instabilities when the reconstructed PDFs are reinserted into a lattice Boltzmann solver for further time integration if needed. By learning the PDFs directly, the proposed framework eliminates this reconstruction ambiguity and preserves compatibility with the full kinetic structure of the LBE, ensuring that the learned fields can be stably advanced by any downstream LBM procedure if desired."

Additionally, we have performed a systematic evaluation of macroscopic-variable prediction using the same architecture and training epochs as employed for our single-particle distribution function models, and using exactly the same von Kármán dataset. The corresponding results are shown below.

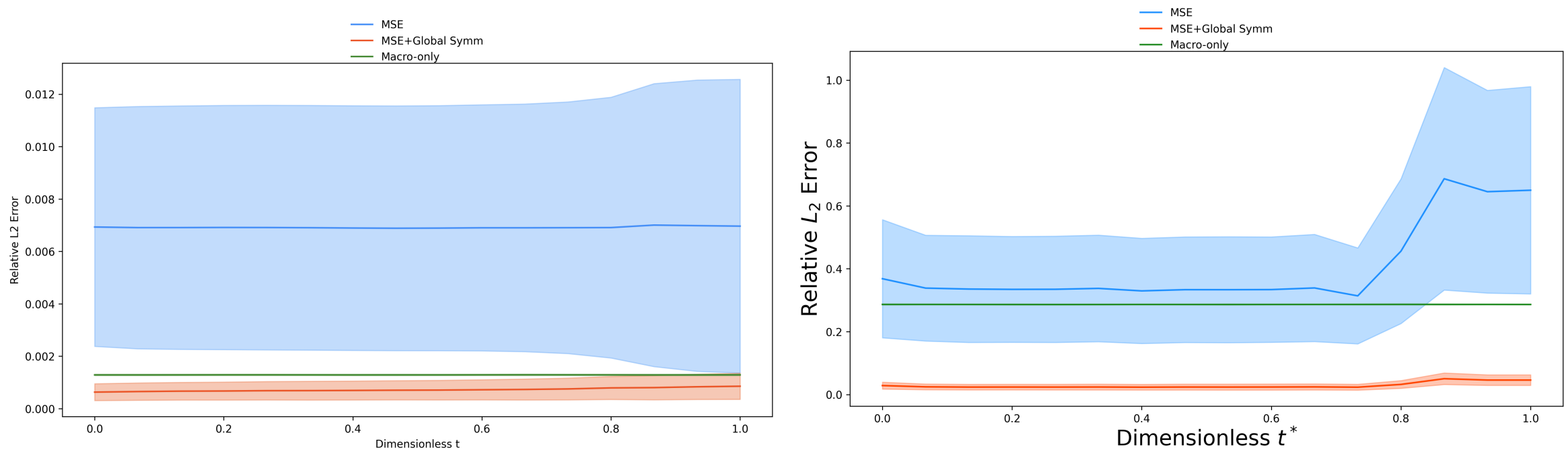

Figure 1: Relative L_2 error for density (left) and velocity magnitude (right) in the von Kármán vortex street, comparing three training strategies using the same architecture and training conditions: kinetic learning with global symmetry (orange), kinetic learning with standard MSE loss (blue), and macroscopic-only learning (green). Shaded regions indicate one standard deviation computed over ten independently trained models. The global symmetry formulation yields the lowest error and the smallest variance for both density and velocity. While macroscopic-only learning produces competitive density accuracy, the kinetic models achieve substantially better velocity prediction, with the gap widening toward later stages of the rollout.

von Karman Vortex Street Figure 1 shows that the kinetic models achieve lower and more stable errors than the macroscopic-only model for the velocity field, particularly during the later stages of the rollout where long-term accumulation effects appear. This improvement may arise because the kinetic formulation has access to the richer single-cell information encoded in the full set of distribution functions, which the macroscopic variables alone do not provide. For density, the macroscopic-only and kinetic models achieve similar accuracy, which is expected because density is primarily determined by the zeroth moment. Nevertheless, when predicting velocity—which depends on higher-order moments—the kinetic models significantly outperform the macroscopic baseline. The global symmetry constraint consistently reduces variance and improves stability, highlighting its importance for long-horizon kinetic prediction.

Accordingly, we have adjusted the text in Section III A, page 11:

"In Fig. 3 **c**, we present the relative L_2 error in the macroscopic predictions obtained using the MSE and MSE+Phys loss functions. The plots show the evolution of the error over normalized time $t^* = t/t_{end}$ with $t_{end} = 6000\Delta t$ for velocity (top) and density (bottom), comparing models trained with standard MSE loss (blue) and physics-informed MSE+Phys loss (red). Importantly, we also compare against training with macroscopic data alone (green). Shaded regions denote the standard deviation over ten independently trained models. The MSE+Phys kinetic formulation consistently outperforms both the MSE-only kinetic model and the macroscopic-only baseline, yielding lower mean error and reduced variance across the prediction horizon. This improvement stems from the richer single-cell information contained in the full set of kinetic distribution functions, which the macroscopic variables alone cannot provide using the same architecture. The advantage of the kinetic evolution becomes increasingly pronounced at later stages of the rollout, particularly for the velocity field, underscoring the stabilising influence of the physics-based constraints during predictions."

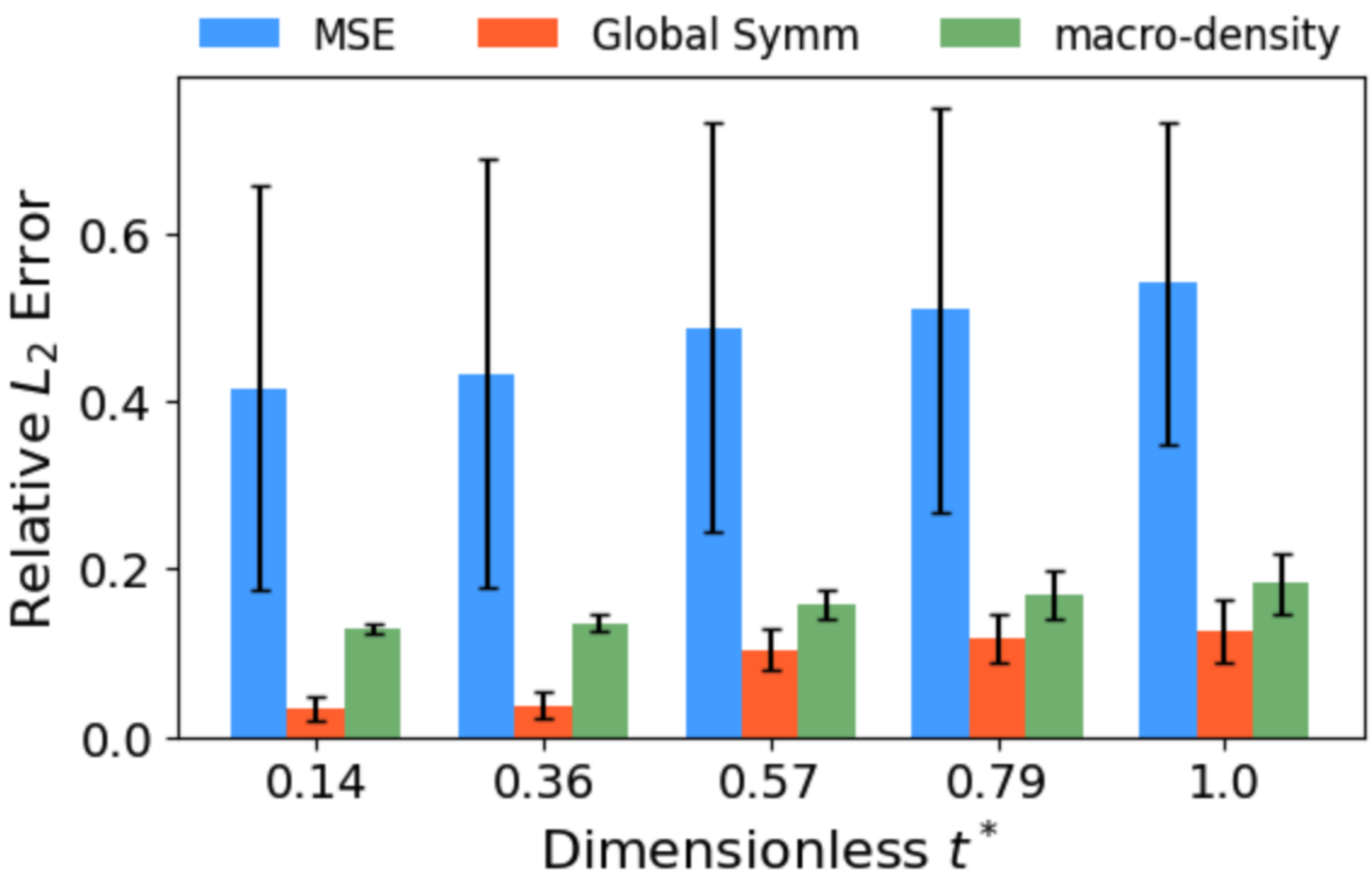

Figure 2: Relative L_2 error of the reconstructed density field for the ligament breakup case, evaluated at five dimensionless times $t^* = 0.14,\ 0.36,\ 0.57,\ 0.79,\ 1.0$. Three learning strategies using the same architecture and training conditions are compared: kinetic learning with global symmetry (orange), kinetic learning with standard MSE loss (blue), and macroscopic-only density prediction (green). Bars indicate one standard deviation over ten independently trained models. The standard kinetic MSE model exhibits the largest error and variance, while the global symmetry formulation significantly stabilises the kinetic evolution and yields the lowest overall errors. The macroscopic-only model performs competitively for this case because density is the primary state variable in this phase-field formulation. Nevertheless, global symmetry formulation yields the lowest overall errors.

Ligament breakup For the ligament breakup case, shown in Fig. 2, the behaviour of the three learning strategies differs from the vortex street example. Here, the primary quantity governing the interface dynamics is the density and the macroscopic field already captures much of the relevant structure. As a result, the macroscopic-only model achieves competitive accuracy. The standard kinetic MSE model, however, displays significantly higher error and variance, reflecting the increased sensitivity of the kinetic fields during the breakup process. In contrast, the global symmetry kinetic model remains consistently accurate and well-behaved across all evaluation times, comfirming that the additional symmetry-based constraint stabilises the kinetic evolution. **While macroscopic prediction may be sufficient for reconstructing density in this specific phase-field system, it cannot recover the full distributions and thus cannot replace LBM at the kinetic level.** Learning the PDFs is the only viable route for developing a general surrogate for LBM time advancement.

Accordingly, we have adjusted the text in Section III B, page 14:

"Figure 4 **c** shows the relative L_2 error of macroscopic predictions evaluated at five normalised time points $t^* = 0.14,\ 0.36,\ 0.57,\ 0.79,\ 1.0$. Error bars represent 95% confidence intervals across 10 independently trained models. The kinetic MSE model exhibits the largest error and variance, reflecting the sensitivity of the PDF fields during the interface-breakup process. The macroscopic-only model achieves competitive accuracy, which is expected since density is

the primary state variable governing dynamics in this system. In contrast, the MSE+Phys kinetic model consistently produces the lowest error and the smallest variance across the full prediction horizon. This indicates that the physics-based constraint stabilises the kinetic evolution and allows the model to exploit the richer single-cell information contained in the PDF representation. This result highlights the improved stability and predictive accuracy achieved by incorporating the physics-informed loss during training."

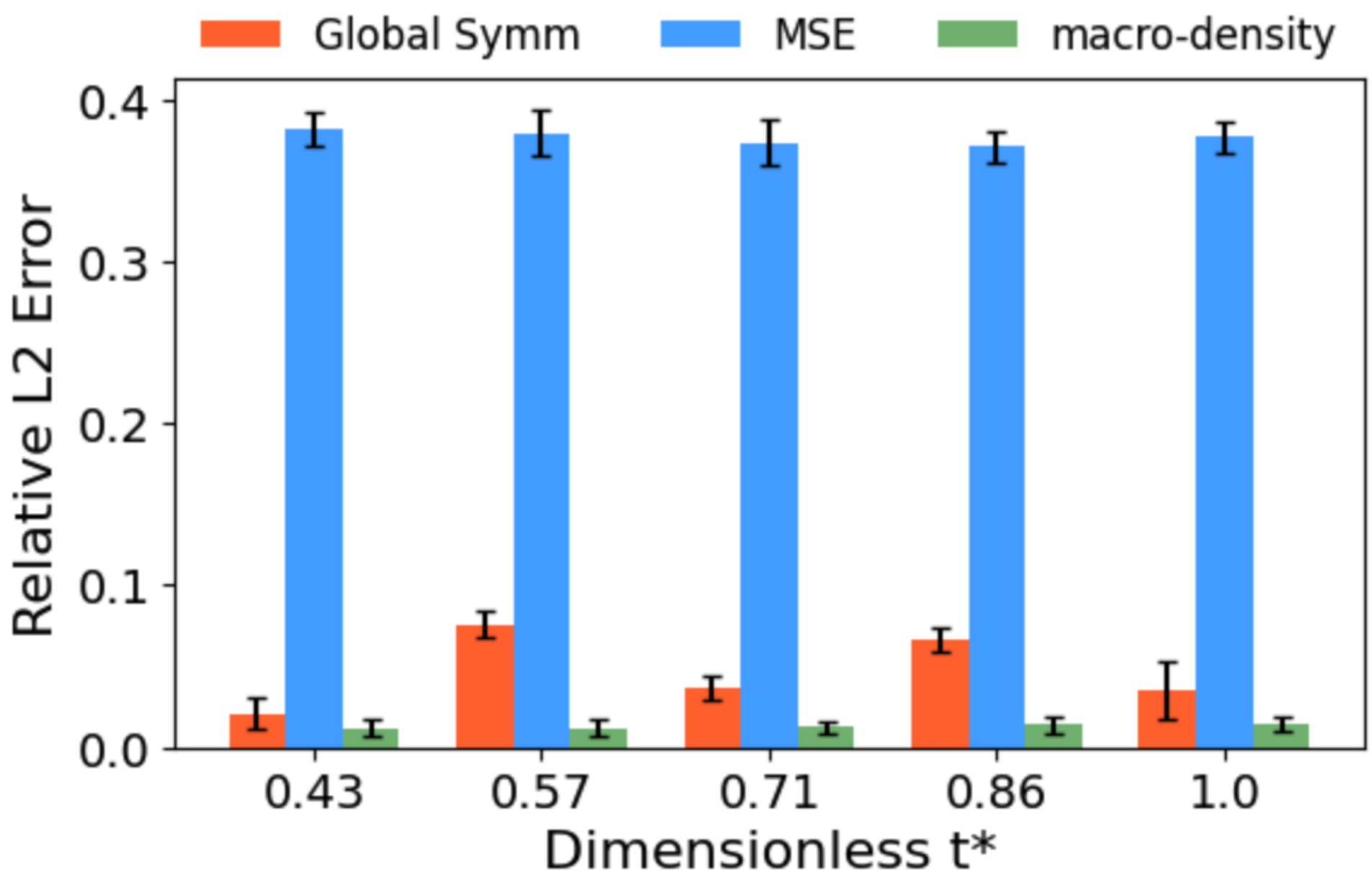

Figure 3: Relative L_2 error of the reconstructed phase-field variable ϕ for the bubble adhesion case, evaluated at five dimensionless times. Three learning strategies are compared: kinetic learning with global symmetry (orange), standard kinetic learning (blue), and macroscopic-only ϕ prediction (green). Error bars represent the standard deviation over ten independently trained models. The phase-field model evolves a single smooth scalar variable with weak kinetic non-equilibrium components. As a result, direct macroscopic prediction achieves lower error, while kinetic learning remains stable and competitive, with the global symmetry formulation providing the most robust kinetic prediction.

Bubble adhesion For the bubble adhesion case (Fig. 3), the error trends differ from the vortex and ligament examples. This system uses a D2Q9 phase-field LBM model in which the primary dynamical quantity is the scalar order parameter ϕ. Predicting ϕ directly is therefore a significantly lower-dimensional task than predicting the entire set of nine PDFs and reconstructing ϕ via moment operations. As a result, macroscopic-only learning achieves the smallest L_2 error in this case. In contrast, kinetic learning must reconstruct the full PDF set, which increases the complexity of the prediction problem. Nevertheless, the kinetic models remain stable and accurate, and the global symmetry formulation provides the best performance among the kinetic variants. **Importantly, macroscopic-only learning cannot be used to recover the PDFs, and therefore cannot serve as a surrogate kinetic solver.** This result is consistent with our broader observation that kinetic learning provides the greatest benefit when accurate PDF reconstruction is required, even when macroscopic predictions alone may appear

competitive for scalar phase-field variables.

We have therefore updated the text in Section III C, page 16:

" A comparative evaluation of the relative error is shown in Fig.5**c**. Using the same architecture, dataset, and training conditions, the standard kinetic MSE model exhibits the largest error and variance throughout the prediction horizon. The macroscopic-only model achieves the lowest error for the phase-field variable, which is expected because phase is the primary scalar quantity evolved in this D2Q9 phase-field system. The MSE+Phys kinetic model, however, provides a clear improvement over the MSE-only kinetic formulation, yielding significantly lower error and markedly reduced variance across all time instances. Error bars indicate the 95% confidence interval across the ensemble of trained models. These results demonstrate that the physics-based constraint stabilizes the kinetic evolution, even though direct macroscopic prediction remains highly effective for this scalar phase-field variable. Importantly, although macroscopic prediction is highly effective for this scalar field, predicting a single macroscopic variable cannot reconstruct the full set of PDFs, and therefore cannot serve as a surrogate for accelerating the underlying LBE solver. This physics-based constraint thus remains essential when the goal is to learn the forward kinetic evolution. "

Across the three benchmark cases, our comparison highlights complementary insights regarding kinetic and macroscopic learning. First, in the D3Q19 von Kármán and ligament-breakup cases, kinetic learning yields more accurate macroscopic predictions than macroscopic-only learning. This improvement may arise because the kinetic model, using the same architecture and dataset, can exploit internal correlations among the 19 distribution functions and leverage the richer single-cell information they encode. Second, in the D2Q9 bubble-adhesion case, the kinetic and macroscopic models achieve comparable accuracy, consistent with the fact that the phase-field dynamics evolve a single scalar order parameter, making direct macroscopic prediction a lower-dimensional task. We emphasize that our goal is not to claim that kinetic learning universally outperforms macroscopic models but rather to demonstrate an alternative route for accelerating LBM simulations. Since a full LBM update requires reconstructing the PDFs via $f = f^{\mathrm{eq}} + f^{\mathrm{neq}}$, missing macroscopic information can easily lead to an ill-posed recovery of the non-equilibrium component. Directly learning the forward evolution of the PDFs provides a more reliable way to preserve non-equilibrium effects and a faithful surrogate for the kinetic LBM operator. Across all cases, despite the larger memory footprint, learning the kinetic fields provides a richer and more physically consistent representation of the LBM dynamics, and serves as a necessary foundation whenever accurate PDF recovery or kinetic-level acceleration is required.

As a result of this discussion of the reviewer's comments we have added the following sentences to the conclusion on Page 17:

"Nevertheless, across the benchmark cases, our comparison shows that kinetic learning provides clear benefits in D3Q19 systems, where the richer PDF representation captures internal correlations that improve macroscopic accuracy, while yielding comparable performance to macroscopic only models in the D2Q9 phase field case. Despite the higher memory cost, directly learning the PDF evolution remains a reliable and physically consistent route for accelerating lattice Boltzmann simulations, as only the kinetic formulation preserves the non equilibrium in-

formation that cannot be recovered from macroscopic simulations variables when higher order moment information is missing."

R1.2 *To start with the issue of unvalidated simulations, the authors present three canonical benchmark cases for which reliable reference data are available. However, they do not validate their own lattice Boltzmann results against any known result. In particular, the von Karman vortex street example displays clear signs of numerical instability near the cylinder, as well as spurious oscillations originating from the inlet. Given the simplicity of this test case, these issues raise serious concerns about whether a state-of-the-art numerical setup was employed. The authors should have considered for instance using a modern LBM collision model, such as the cumulant approach, to mitigate these instabilities. As a consequence of these deficiencies, the neural operator is trained on data that contain evident numerical errors and, concerningly, reproduces the same incorrect instability pattern with high accuracy. This outcome weakens the claim that the framework is physics-informed and may even, to the contrary, lead to the impression that it primarily reproduces patterns present in the training data.*

Part of the difficulty likely stems from the small computational domains, which appear to have been chosen to satisfy the memory constraints of the machine-learning framework. While such limitations are understandable, the manuscript should include a clear and quantitative discussion of the memory restrictions, the corresponding design choices, and the implications for the fidelity and interpretability of the simulations performed under these conditions.

Response to R1.2 We thank the reviewer for raising concerns regarding the quality of the LBM reference simulations. We respectfully note that the LBM solver employed in this work is a standard and well-established implementation that has been extensively tested and used in many prior publications[1,2,3]. For the von Kármán vortex street at $Re = 180$, the BGK + SGS collision models is used for this benchmark, and our simulation parameters follow commonly accepted setups in the LBM literature[1,4]. We have also performed simulations with BGK + SGS collision models at a higher resolution, when the spurious currents are eliminated, with proper citations included. Importantly, our kinetic model is still capable of predicting the higher resolution model as well. We have added it to our text accordingly in the discussion of the Application Section A page 10:

"In Fig.3 **b**, the top row shows the ground-truth fields at 160×160 based on LB solver [**?**]. The middle row presents the absolute error of the MSE+Phys prediction on the low-resolution (LR) grid (80×80), which is the resolution used for training. Each prediction step is $\Delta t_n = 50\Delta t$. The bottom row displays the absolute error at super-resolution, obtained through direct inference of the same model on the 160×160 grid without any retraining. Across all frames, both the low-resolution and super-resolution errors remain within 0 to 1×10^{-4}, indicating that the learned operator preserves accuracy even when evaluated at a resolution twice as fine as the training grid. Also, for the super resolution dataset collision relaxation time differs from the LR value. This demonstrates that the model is able to capture the fine-scale with the kinetic data and smoothly across spatial resolutions and supports super-resolution inference."

We have also made adjustments for Application Section A, page 11:

"Figure 3 **d** shows the density evolution obtained through the kinetic data following Eq.8. Due to the weak compressibility of LBE[12], the predicted field captures minor density oscillations with the help of the MSE+Phys constraint, indicated by dark blue circles, which align well with the ground truth. The top row shows the reference density fields at 160×160, while the middle row displays the absolute error at the low-resolution, corresponding to the training resolution. The bottom row shows the absolute error at 160×160, obtained by direct super-resolution inference without retraining the model. Across all time instants, the absolute density error remains below 1×10^{-2}, indicating that the learned operator achieves highly accurate macroscopic reconstruction with distribution functions even when evaluated on a grid twice as fine as the training resolution."

Source code citations:

1. Xue, Xiao, Hua-Dong Yao, and Lars Davidson. "Synthetic turbulence generator for lattice Boltzmann method at the interface between RANS and LES." Physics of Fluids 34.5 (2022).

2. Chiappini, Daniele, et al. "Hydrodynamic behavior of the pseudopotential lattice Boltzmann method for interfacial flows." Physical Review E 99.5 (2019): 053305.

3. Krüger, Timm, et al. The lattice Boltzmann method. Vol. 10. No. 978-Cham: Springer International Publishing, 2017.

For the other cases, we also have now cited the LB solver we used accordingly[2,3]. Our objective was not to reproduce a full-scale CFD benchmark but to generate consistent kinetic-level data for training a LB machine learning surrogte model. Since the model learns local spatiotemporal correlations in the PDFs, a compact domain is fully sufficient for training and is widely used in operator-learning contexts.

We have updated text in the conclusion page 17:

"The kinetic representation naturally requires more memory than macroscopic fields (19 versus 4 variables per cell in D3Q19 and 9 versus 3 in D2Q9), which limits the feasible training domain size. Nevertheless, across the benchmark cases, our comparison shows that kinetic learning provides clear benefits in D3Q19 systems, where the richer PDF representation captures internal correlations that improve macroscopic accuracy, while yielding comparable performance to macroscopic only models in the D2Q9 phase field case. Despite the higher memory cost, directly learning the PDF evolution remains a reliable and physically consistent route for accelerating lattice Boltzmann simulations, as only the kinetic formulation preserves the non equilibrium information that cannot be recovered from macroscopic simulations variables when higher order moment information is missing."

R1.3 Small computational domains and memory limitations. *The manuscript uses very small computational domains, likely due to the memory constraints of the machine-learning framework. These constraints, design choices, and their implications should be discussed quantitatively.*

Response to R1.3 We thank the reviewer for raising this important point regarding dataset size. We agree that data volume plays a crucial role in the stability and generalisation of

learning-based approaches. In our setting, however, each training sample consists of full spatiotemporal fields of all discrete distribution functions $\{f_i\}$, which makes kinetic data inherently much higher dimensional than macroscopic datasets. For example, a single D3Q19 kinetic snapshot contains 19 scalar fields over the entire computational domain, meaning that even a “small” number of samples corresponds to larger of effective training degrees of freedom. The primary limitation on dataset size is computational feasibility: generating kinetic training data at high temporal resolution and storing multi-channel LBM fields is significantly more memory expensive than producing macroscopic data. Increasing dataset size further would incur prohibitive memory and computational costs, particularly for three-dimensional cases. Nevertheless, we emphasise that the presented datasets are sufficient to demonstrate consistent accuracy across all three flow scenarios, as supported by our numerical evaluations.

To clarify these points, we have added a statement in the Methods section explaining why kinetic datasets contain substantially more information per sample than macroscopic datasets and why compact datasets are therefore sufficient for training. (See Page 19)

“Note that each kinetic snapshot contains all discrete distribution functions across the full spatial domain (e.g., 19 variables per cell in D3Q19 and 9 in D2Q9), the memory footprint of kinetic datasets is larger than that of macroscopic-only datasets. This high dimensionality limits feasible dataset sizes and batch sizes during training.”

R1.4 Unclear novelty and unclear advantage of predicting PDFs. *The novelty appears to rest on applying the operator to PDFs rather than velocity fields, but the manuscript does not articulate the advantage of this choice. The text repeatedly emphasizes “kinetic” aspects, yet predicting PDFs requires learning many more fields (19 instead of 3–4). Moreover, best results occur when combining PDF-based and macroscopic losses, suggesting the method is hybrid rather than kinetic. This raises the question of whether PDFs are needed at all.*

Response to R1.4 We thank the reviewer for raising this point. Our intention is not to claim that predicting PDFs is universally superior to predicting macroscopic variables for all CFD applications, but rather to highlight why it is specifically appropriate in the context of accelerating the kinetic time-advancement of LBM. The LBM dynamics are defined at the level of the discrete particle distribution functions, from which macroscopic fields are obtained via moment operations. Predicting the PDFs therefore preserves the full non-equilibrium kinetic state, which is essential for multiphysics LBM models such as the Shan-Chen and phase-field formulations used in our study. These models rely explicitly on non-equilibrium contributions that cannot be reconstructed uniquely from macroscopic variables alone. As stated in Figure 1, PDF prediction provides a more accurate prediction of both density and velocity in the same training setup.

While macroscopic-only neural operators are suitable for accelerating direct PDE solvers, our objective here is to provide a drop-in surrogate for the LBM kinetic update itself which may accelerating the LBE solvers. A purely macroscopic PINO would require reconstructing PDFs at every step before the solver could advance, which is an ill-posed inversion (non-equilibrium reconstruction is limited and non-trivial) and can introduce significant instability. The hybrid loss used in our framework reflects the multiscale structure of LBM: the kinetic loss preserves

the non-equilibrium information, while the macroscopic loss enforces global conservation and stability.

In the revised Introduction we now explicitly state that our motivation for predicting PDFs (See Response to 1.1 & Response to 1.3) is tied to accelerating the kinetic-level LBM evolution, preserving non-equilibrium information required by multiphase and force-coupled LBM models, and avoiding the instabilities associated with reconstructing PDFs from macroscopic variables. We also clarify that the contribution of this work lies in learning the kinetic operator itself, rather than proposing an alternative macroscopic CFD solver.

R1.5 Unsupported computational speedup claims. *The claim of 2-3 orders of magnitude speedup is unsupported because the LBM solver was run on a CPU (Intel Core-i7) while the ML model was run on an NVIDIA RTX 4090 GPU. A fair comparison requires running LBM on the same GPU hardware. Furthermore, run-time documentation (domain size, iteration counts) is insufficiently detailed to independently verify the reported timings.*

Response to R1.5 We thank the reviewer for raising this point. Since the numerical LBM solver used in this study does not have a GPU implementation, all baseline LBM timings were obtained on CPU hardware. To ensure full transparency and a fair comparison, we have recomputed all LBM timings in single precision (FP32), matching the precision used by the ML surrogate. This removes any ambiguity regarding floating-point differences. The neural model was trained on an NVIDIA A100 80 GB GPU, while the originally reported inference timings were obtained on our Intel(R) Xeon(R) Gold 6254 CPU @ 3.10 GHz. For consistency, we have rerun all benchmarks on the same CPU using FP32 for both methods, and we additionally report GPU inference times for completeness. The revised manuscript now includes a hardware- and precision-consistent performance comparison, with updated timing results reflecting this unified FP32 benchmarking.

We have adjusted the text in the Supplementary Information Section S3, Page 11:

"Instead of computing every intermediate timestep as in traditional LB solvers, the predictor effectively skips multiple timesteps, yielding substantial runtime reductions. In the revised manuscript, all model training is performed on a single NVIDIA A100 80 GB GPU, while all inference benchmarks are executed on our inference hardware, an Intel(R) Xeon(R) Gold 6254 CPU @ 3.10 GHz. Since the reference LB solver is a CPU-only implementation, we executed it in single precision (FP32) to ensure a fair comparison with the neural predictor. Accordingly, all reported speedup results now use matched FP32 precision on the same CPU hardware. A detailed comparison of the updated runtimes and effective domain size is provided in Table S2."

We have provide new table regarding detailed LB simulation domain size and effective domain size for training in the Supplementary information Page 41:

**Examples**	Domain size	$T_{\mathbf{LB}}^{\mathbf{total}}$	Effective Domain size	$T_{\mathbf{LB}}^{\mathbf{eff}}$
Kármán vortex street LR (c++)	$110 \times 80 \times 3$ LU	17.24 ± 1.8s	80×80 LU	4.18 ± 0.5s
Liquid ligament breakup (c++)	$48 \times 128 \times 48$ LU	14.88 ± 1.5s	128×48 LU	0.31 ± 0.03s
Bubble adhesion in phase space	100×300 LU	104.34 ± 3.4s	84×78 LU	22.79 ± 0.74s

Table 1: Comparison of full-domain and effective-domain LB simulation runtimes for the three benchmark examples. All LB simulations were executed in single precision (FP32) on the Intel(R) Xeon(R) Gold 6254 CPU and repeated ten times; reported values denote the mean $\pm$ standard deviation. $T_{\text{LB}}^{\text{total}}$ corresponds to the runtime of the full-domain LB simulation, while $T_{\text{LB}}^{\text{eff}}$ denotes the runtime when restricting computation to the effective domain. Domain sizes are reported in lattice units (LU).

We have added a speed-up table to the main manuscript (Page 30):

**Examples**	**Simulation time (CPU)**	**Prediction time (CPU)**	**Acceleration**
Kármán vortex street LR (c++)	4.18 ± 0.5s	$0.025 \pm 0.0012s$	$\approx$ 167.2 times
Liquid ligament breakup (c++)	0.31 ± 0.03s	0.043 ± 0.0011s	$\approx$ 7.2 times
Bubble adhesion in phase space	22.79 ± 0.74s	$0.043 \pm 0.0018s$	$\approx$ 530.08 times

Table 2: Comparison of simulation and prediction runtimes. All LB simulations were measured in single precision (FP32) and repeated ten times; the reported values denote the mean, with the accompanying "$\pm$" indicating the standard deviation. All ML predictions were also evaluated in FP32 and repeated one hundred times, with mean and standard deviation reported in the same format. The precise definition of the acceleration metric is provided in Supplementary Information S3.

R1.6 Unclear meaning of "predictive", "cross-collision", and "cross-physics". *The manuscript states that the model is predictive, cross-collision, and cross-physics, but these terms are unclear. All examples use consistent physics and collision models between training and inference. No example demonstrates transfer across physics or collision models. In the von Kármán case, the model reproduces the same oscillation window sampled during training; in the multiphase cases, only initial conditions vary. Thus, no data supports cross-physics applicability.*

Response to R1.6 We thank the reviewer for pointing out the ambiguity in these terms. Our intention was not to claim transfer across fundamentally different physical models, but rather to highlight that the proposed neural operator is able to learn the forward kinetic evolution independently of the specific collision operator or multiphysics formulation used to generate the data. In other words, the same operator architecture and loss design apply equally to BGK,

MRT, Shan-Chen, and phase-field LBM datasets without requiring model-specific modifications. Each dataset is trained and inferred under consistent physics, but the methodology itself is not restricted to one collision model or one class of LB dynamics.

To avoid any misunderstanding, we have revised the manuscript to clarify that the framework is "collision-model-agnostic" and "applicable across multiple LBM formulations," rather than implying cross-physics generalisation in the sense of training on one physical model and inferring a different one.

In the abstract, we changed the text accordingly to avoid misunderstandings:

"In addition, the framework is agnostic to the specific LBM formulation, allowing the same neural operator architecture and loss design to be applied to different kinetic datasets, each trained within its own governing dynamics."

R1.7 Unsupported claim of super-resolution. *The manuscript claims super-resolution but provides no evidence that the operator recovers subgrid structures beyond interpolation. The test cases are laminar and smooth, making it difficult to show true super-resolution. A quantitative comparison with high-resolution reference solutions would be required.*

Response to R1.7 We thank the reviewer for the comments. In response, we have now performed LBM simulations on a 160×160 grid using appropriately adjusted relaxation times to maintain the same Reynolds number, and we compare the corresponding errors. These updated results are included in the revised Figure 3. In addition, we have expanded our discussion of kinetic super-resolution in the abstract to clarify our assessment.

We have modified the abstract to read:

Our framework is discretization-invariant, enabling models trained on coarse-grained PDFs to perform inference on finer spatial resolutions, even when the intrinsic relaxation-time parameter differs between the coarse and fine lattices.

Additional Comments

R1.8 Inappropriate citation regarding derivation of continuum equations. *On page 3, citing a non-peer-reviewed preprint (Deng et al.) as "major progress" in rigorously deriving continuum equations from particle dynamics is not appropriate, given the long-standing literature in this field.*

Response to R1.8 We thank the reviewer for the comment. We agree that the reference to Hilbert's sixth problem and its surrounding discussion are not essential for the goals of this manuscript and may distract from the main narrative. We have therefore removed this material entirely from the revised Introduction. The updated text now focuses solely on the motivation and methodological aspects directly relevant to the lattice Boltzmann framework and the proposed operator-learning approach.

R1.9 Incorrect statement about time-step restrictions of LBM. *The abstract states that LBM is limited to small time-steps due to restrictions imposed by collision kernels. A*

clearer explanation is that small time-steps arise from explicit CFL-like stability constraints, similar to other explicit schemes.

Response to R1.9 We have made the following adjustment to our abstract

Despite its success, solving the LBE numerically remains computationally intensive due to the small time steps required by explicit kinetic schemes, which are subject to CFL-like stability constraints.

Response to Reviewer #2

We thank Reviewer #2 for the thorough evaluation of our work and for highlighting several areas for clarification and improvement. We address each point in detail below.

Reviewer #2 (Remarks to the Author)

R2.1: Paper organization

The reviewer argues that the manuscript is poorly organized, that critical methodological details appear too late in the paper, and that the supplemental material is overly relied upon.

Response to R2.1

We thank the reviewer for the organisational issues in the manuscript. In fact, we are following Nature's format to organize our paper accordingly, and we do agree that we can further improve the structure enhances readability, and we have revised the manuscript accordingly.

1. We have reorganized the manuscript by moving several key figures from later sections to earlier locations so that they appear where they are first discussed. Specifically, Figure 3 has been moved to Page 10, Figure 4 to Page 13, and Figure 5 to Page 15.

2. In accordance with the Nature Portfolio formatting guidelines, which require the Methods section to appear after the Results, we have kept the detailed methodology in the Methods section while avoiding excessive technical detail.

3. We have also relocated several important results, including the acceleration benchmarks, super-resolution demonstrations, and their supporting explanations, from the Supplementary Information into the main manuscript, reducing the need to refer back to the Supplementary Information and presenting the primary findings more cohesively.

The acceleration results previously presented in Supplementary Information Table S2 have been moved into the main manuscript on Page 30 to improve accessibility and reduce reliance on the Supplementary Information. Super-resolution demonstrations have been added on Figure 3.

R2.2: Motivation and missing connections to prior kinetic literature

The reviewer states that although the manuscript motivates the surrogate operator by citing the time–step restriction from the collision operator, it fails to relate this issue to existing literature on kinetic models, such as penalty methods or multi-scale approaches.

Response to R2.2

We appreciate the reviewer's insightful comments regarding the broader context of time-step acceleration techniques in kinetic models. We agree that situating our approach within the wider landscape of kinetic modeling methods will strengthen our manuscript, and we have revised the text to clarify the relationship between our neural-operator framework and existing strategies in the literature.

In the revised manuscript, we have added a dedicated paragraph in the Introduction that explicitly discusses prior work on accelerating kinetic solvers, including penalty methods for the Boltzmann collision operator (e.g., Filbet & Jin, JCP 2010) and related multiscale techniques (See Introduction, Page 4):

"While several alternative kinetic schemes exist for handling collisional stiffness, such as penalty methods for the Boltzmann equation [filbet2011asymptotic], implicit Fokker-Planck formulations [taitano2017equilibrium], and classical implicit BGK or Lenard-Bernstein approaches [coron1991numerical,habbershaw2025implicit], these methods are formulated for continuum kinetic models and do not directly apply to the discrete-velocity lattice Boltzmann equation. In contrast, our focus is specifically on LBE-orientated acceleration, which has seen only limited data-driven attempts. The existing approaches are trained to approximate more general scenario without explicitly incorporating the discrete LB lattice structure as an inductive bias."

We have also expanded the discussion to highlight how our method differs from implicit or asymptotic-preserving approaches commonly used in kinetic theory. These revisions clarify that our framework is not intended to replace traditional numerical schemes but rather to provide a complementary, data-driven surrogate capable of extrapolating over large temporal scales. Importantly, it is fitted for the lattice Boltzmann solvers other than other numerical approaches, we have stated this clearly in the revised manuscript (See Introduction, Page 4):

"...these methods are formulated for continuum kinetic models and do not directly apply to the discrete-velocity lattice Boltzmann equation. In contrast, our focus is specifically on LBE-orientated acceleration, which has seen only limited data-driven studies."

R2.3: Missing discussion of implicit collision treatments

The reviewer points out that many kinetic models (e.g., BGK, Fokker–Planck, Lenard–Bernstein) admit implicit discretization of the collision operator, and that the manuscript should acknowledge or compare to this direction.

Response to R2.3

We thank the reviewer for highlighting the extensive body of work on implicit treatments of collision operators in broader kinetic theory. We agree that these methods provide important historical context; however, our study is deliberately focused on the lattice Boltzmann method (LBM) and its BGK-based collision model, rather than on general kinetic solvers for the Boltzmann or Fokker–Planck equations. As such, while implicit schemes are relevant at a conceptual level, they fall outside the primary scope of this LBM-centered work.

To address this point, we have added a brief paragraph in the Introduction acknowledging classical implicit treatments of collision operators in kinetic models (e.g., BGK and Fokker–Planck formulations) to provide proper context without shifting the paper's focus away from LBM. Beyond this introductory clarification, we have not expanded the main text further, in order to maintain the LBM-specific scope of the manuscript.

We declared this point in Section Introduction, Page 4:

"While several alternative kinetic schemes exist for handling collisional stiffness, such as penalty methods for the Boltzmann equation[filbet2011asymptotic], implicit Fokker–Planck formulations [taitano2017equilibrium], and classical implicit BGK or Lenard–Bernstein approaches [coron1991numerical,habbershaw2025implicit], these approaches target general kinetic solvers rather than the lattice Boltzmann equation. In contrast, our focus is specifically on LBE-orientated acceleration, which has seen only limited data-driven studies."

We have also revised the caption of Figure 1 to better position the manuscript within the broader context of existing data-driven lattice Boltzmann approaches.

"A multiscale framework for lattice Boltzmann equation encompassing microscopic, mesoscopic, and macroscopic perspectives. Panel **a** outlines the kinetic data-driven hybrid ML approach for LBE, where neural networks are used to learn or enhance collision kernels in the lattice Boltzmann equation, a discrete form of the Boltzmann equation."

R2.4: Hydrodynamic limit and motivation for kinetic modelling

The reviewer asks why a neural operator should be trained on the kinetic (LBM) formulation if, at time scales much larger than the relaxation time, the system behaves hydrodynamically. The reviewer questions whether the method captures anything essentially kinetic and why hydrodynamic equations would not suffice.

Response to R2.4

We appreciate the reviewer's thoughtful question regarding the hydrodynamic limit and the relationship between kinetic and continuum descriptions. It is correct that, for flows where the characteristic time scale is much larger than the relaxation time, the LBM recovers the Navier–Stokes equations. However, our work targets scenarios where the full kinetic representation of LBM is still beneficial, such as multiphase dynamics, interfacial forces, and mesoscale interactions, while aiming to accelerate its temporal evolution without discarding its kinetic structure. Our neural operator is designed to learn directly in the space of distribution functions rather than macroscopic fields, allowing the framework to remain compatible with the rich set of LBM collision models, forcing schemes, and multiphase formulations that do not readily reduce to classical hydrodynamics.

To clarify this motivation, we have added a concise explanation in the Introduction that highlights why preserving the kinetic formulation is advantageous for LBM-based multiphase and interfacial problems, and how this differs from learning purely hydrodynamic operators.

In the revised manuscript (Introduction), we have clarified the motivation for predicting PDFs rather than only macroscopic fields as follows: (Introduction, Page 4)

"Our goal is to construct a surrogate for the kinetic time advancement that is intrinsic to the lattice Boltzmann equation. Predicting the discrete single particle distribution functions preserves the full non-equilibrium content of the kinetic description. Importantly, as highlighted in Shan's work[35], the kinetic representation can encode physical effects beyond those captured by macroscopic PDEs. When only low-order macroscopic moments are available, reconstructing the PDFs becomes a non-unique and often ill-posed inverse problem. The macroscopic fields do not contain sufficient information to uniquely determine the non-equilibrium components of the PDFs, and any missing or incomplete moment information leads to reconstruction errors. These errors directly contaminate the non-equilibrium stresses that drive LBE dynamics, and even small inaccuracies can cause numerical instabilities when the reconstructed PDFs are reinserted into a lattice Boltzmann solver for further time integration if needed. By learning the PDFs directly, the proposed framework eliminates this reconstruction ambiguity and preserves compatibility with the full kinetic structure of the LBE, ensuring that the learned fields can be stably advanced by any downstream LBM procedure if desired."

This additional context helps position the method within the LBM framework without extending the scope into a broader comparison with continuum solvers. Additionally, we have performed a systematic evaluation of macroscopic-variable prediction using the same architecture and training epochs as employed for our single-particle distribution function models, and using exactly the same von Kármán dataset. The corresponding results are shown below.

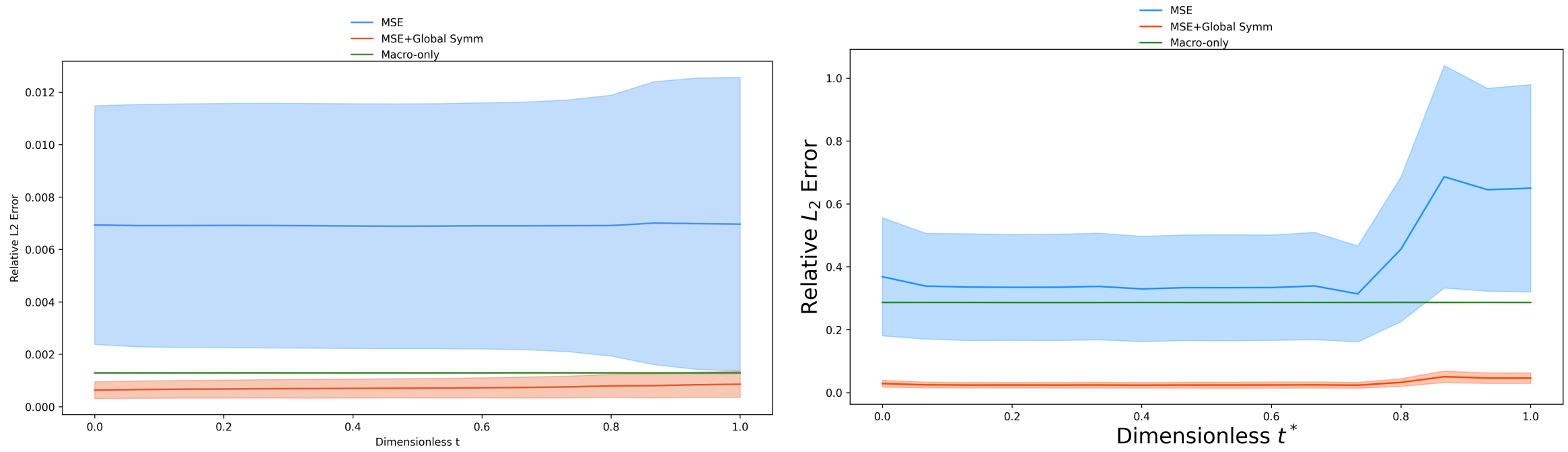

Figure 1: Relative L_2 error for density (left) and velocity magnitude (right) in the von Kármán vortex street, comparing three training strategies using the same architecture and training conditions: kinetic learning with global symmetry (orange), kinetic learning with standard MSE loss (blue), and macroscopic-only learning (green). Shaded regions indicate one standard deviation computed over ten independently trained models. The global symmetry formulation yields the lowest error and the smallest variance for both density and velocity. While macroscopic-only learning produces competitive density accuracy, the kinetic models achieve substantially better velocity prediction, with the gap widening toward later stages of the rollout.

von Karman Vortex Street Figure 1 shows that the kinetic models achieve lower and more stable errors than the macroscopic-only model for the velocity field, particularly during the later stages of the rollout where long-term accumulation effects appear. This improvement may arise because the kinetic formulation has access to the richer single-cell information encoded in the full set of distribution functions, which the macroscopic variables alone do not provide. For density, the macroscopic-only and kinetic models achieve similar accuracy, which is expected because density is primarily determined by the zeroth moment. Nevertheless, when predicting velocity—which depends on higher-order moments—the kinetic models significantly outperform the macroscopic baseline. The global symmetry constraint consistently reduces variance and improves stability, highlighting its importance for long-horizon kinetic prediction.

Accordingly, we have adjusted the text in Section III A, page 11:

"In Fig. 3 **c**, we present the relative L_2 error in the macroscopic predictions obtained using the MSE and MSE+Phys loss functions. The plots show the evolution of the error over normalized time $t^* = t/t_{end}$ with $t_{end} = 6000\Delta t$ for velocity (top) and density (bottom), comparing models trained with standard MSE loss (blue) and physics-informed MSE+Phys loss (red). Importantly, we also compare against training with macroscopic data alone (green). Shaded regions denote the standard deviation over ten independently trained models. The MSE+Phys kinetic formulation consistently outperforms both the MSE-only kinetic model and the macroscopic-only baseline, yielding lower mean error and reduced variance across the prediction horizon. This improvement stems from the richer single-cell information contained in the full set of kinetic distribution functions, which the macroscopic variables alone cannot provide using the same architecture. The advantage of the kinetic evolution becomes increasingly pronounced at later stages of the rollout, particularly for the velocity field, underscoring the stabilising influence of the physics-based constraints during predictions."

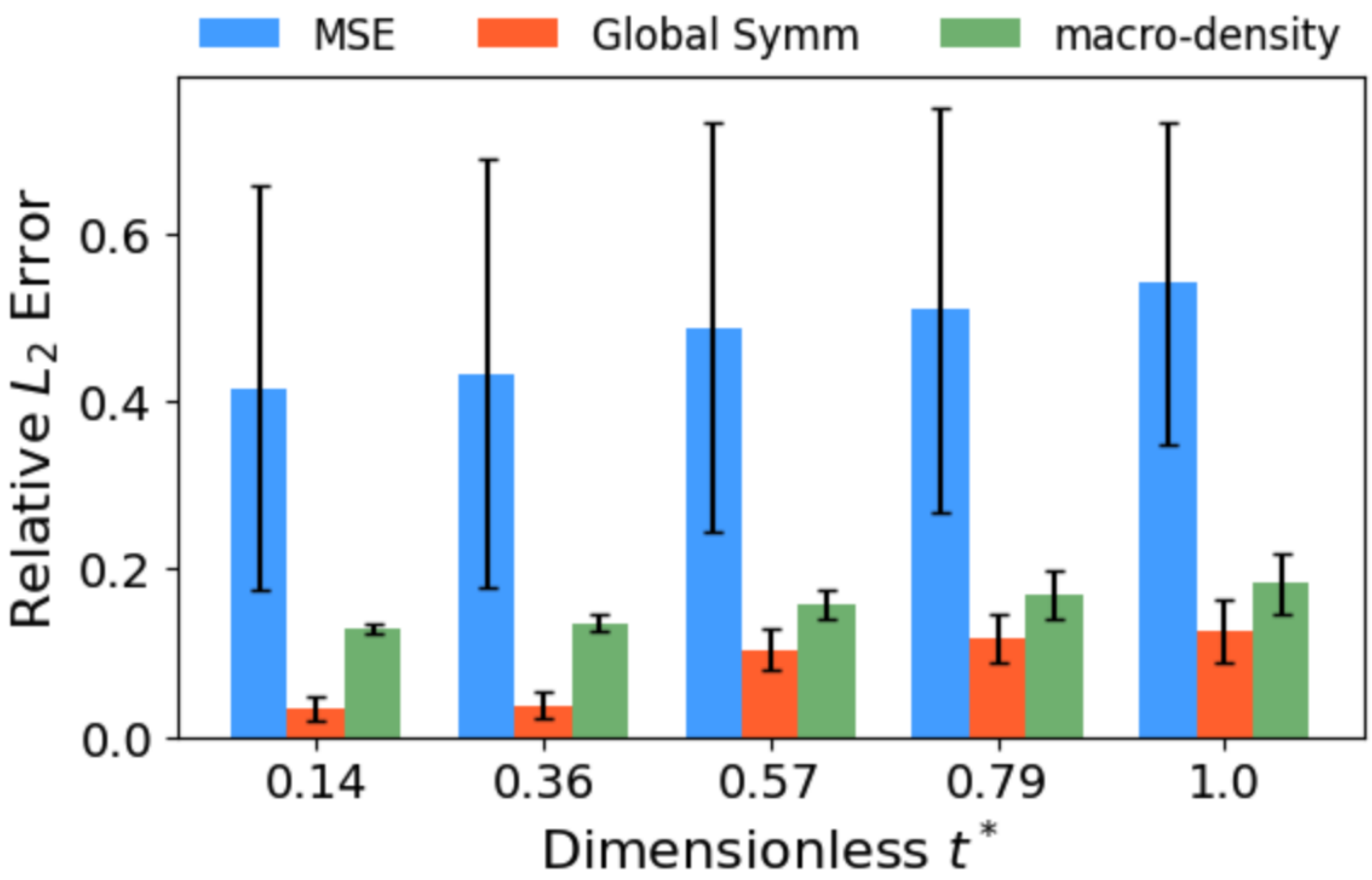

Figure 2: Relative L_2 error of the reconstructed density field for the ligament breakup case, evaluated at five dimensionless times $t^* = 0.14,\ 0.36,\ 0.57,\ 0.79,\ 1.0$. Three learning strategies using the same architecture and training conditions are compared: kinetic learning with global symmetry (orange), kinetic learning with standard MSE loss (blue), and macroscopic-only density prediction (green). Bars indicate one standard deviation over ten independently trained models. The standard kinetic MSE model exhibits the largest error and variance, while the global symmetry formulation significantly stabilises the kinetic evolution and yields the lowest overall errors. The macroscopic-only model performs competitively for this case because density is the primary state variable in this phase-field formulation. Nevertheless, global symmetry formulation yields the lowest overall errors.

Ligament breakup For the ligament breakup case, shown in Fig. 2, the behaviour of the three learning strategies differs from the vortex street example. Here, the primary quantity governing the interface dynamics is the density and the macroscopic field already captures much of the relevant structure. As a result, the macroscopic-only model achieves competitive accuracy. The standard kinetic MSE model, however, displays significantly higher error and variance, reflecting the increased sensitivity of the kinetic fields during the breakup process. In contrast, the global symmetry kinetic model remains consistently accurate and well-behaved across all evaluation times, comfirming that the additional symmetry-based constraint stabilises the kinetic evolution. **While macroscopic prediction may be sufficient for reconstructing density in this specific phase-field system, it cannot recover the full distributions and thus cannot replace LBM at the kinetic level.** Learning the PDFs is the only viable route for developing a general surrogate for LBM time advancement.

Accordingly, we have adjusted the text in Section III B, page 14:

"Figure 4 **c** shows the relative L_2 error of macroscopic predictions evaluated at five normalised time points $t^* = 0.14,\ 0.36,\ 0.57,\ 0.79,\ 1.0$. Error bars represent 95% confidence intervals across 10 independently trained models. The kinetic MSE model exhibits the largest error and variance, reflecting the sensitivity of the PDF fields during the interface-breakup process. The macroscopic-only model achieves competitive accuracy, which is expected since density is

the primary state variable governing dynamics in this system. In contrast, the MSE+Phys kinetic model consistently produces the lowest error and the smallest variance across the full prediction horizon. This indicates that the physics-based constraint stabilises the kinetic evolution and allows the model to exploit the richer single-cell information contained in the PDF representation. This result highlights the improved stability and predictive accuracy achieved by incorporating the physics-informed loss during training."

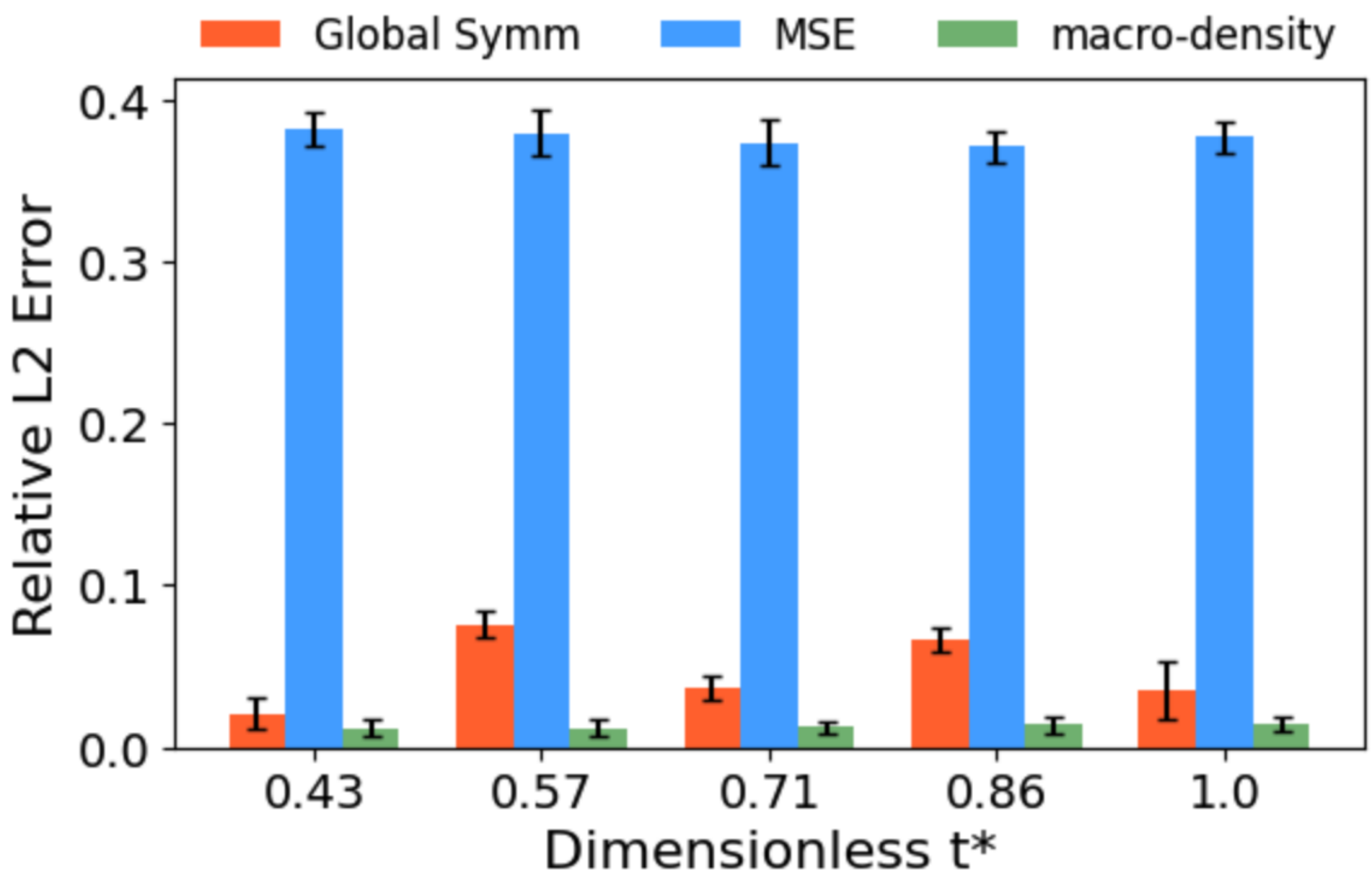

Figure 3: Relative L_2 error of the reconstructed phase-field variable ϕ for the bubble adhesion case, evaluated at five dimensionless times. Three learning strategies are compared: kinetic learning with global symmetry (orange), standard kinetic learning (blue), and macroscopic-only ϕ prediction (green). Error bars represent the standard deviation over ten independently trained models. The phase-field model evolves a single smooth scalar variable with weak kinetic non-equilibrium components. As a result, direct macroscopic prediction achieves lower error, while kinetic learning remains stable and competitive, with the global symmetry formulation providing the most robust kinetic prediction.

Bubble adhesion For the bubble adhesion case (Fig. 3), the error trends differ from the vortex and ligament examples. This system uses a D2Q9 phase-field LBM model in which the primary dynamical quantity is the scalar order parameter ϕ. Predicting ϕ directly is therefore a significantly lower-dimensional task than predicting the entire set of nine PDFs and reconstructing ϕ via moment operations. As a result, macroscopic-only learning achieves the smallest L_2 error in this case. In contrast, kinetic learning must reconstruct the full PDF set, which increases the complexity of the prediction problem. Nevertheless, the kinetic models remain stable and accurate, and the global symmetry formulation provides the best performance among the kinetic variants. **Importantly, macroscopic-only learning cannot be used to recover the PDFs, and therefore cannot serve as a surrogate kinetic solver.** This result is consistent with our broader observation that kinetic learning provides the greatest benefit when accurate PDF reconstruction is required, even when macroscopic predictions alone may appear

competitive for scalar phase-field variables.

We have therefore updated the text in Section III C, page 16:

" A comparative evaluation of the relative error is shown in Fig.5**c**. Using the same architecture, dataset, and training conditions, the standard kinetic MSE model exhibits the largest error and variance throughout the prediction horizon. The macroscopic-only model achieves the lowest error for the phase-field variable, which is expected because phase is the primary scalar quantity evolved in this D2Q9 phase-field system. The MSE+Phys kinetic model, however, provides a clear improvement over the MSE-only kinetic formulation, yielding significantly lower error and markedly reduced variance across all time instances. Error bars indicate the 95% confidence interval across the ensemble of trained models. These results demonstrate that the physics-based constraint stabilizes the kinetic evolution, even though direct macroscopic prediction remains highly effective for this scalar phase-field variable. Importantly, although macroscopic prediction is highly effective for this scalar field, predicting a single macroscopic variable cannot reconstruct the full set of PDFs, and therefore cannot serve as a surrogate for accelerating the underlying LBE solver. This physics-based constraint thus remains essential when the goal is to learn the forward kinetic evolution. "

Across the three benchmark cases, our comparison highlights complementary insights regarding kinetic and macroscopic learning. First, in the D3Q19 von Kármán and ligament-breakup cases, kinetic learning yields more accurate macroscopic predictions than macroscopic-only learning. This improvement may arise because the kinetic model, using the same architecture and dataset, can exploit internal correlations among the 19 distribution functions and leverage the richer single-cell information they encode. Second, in the D2Q9 bubble-adhesion case, the kinetic and macroscopic models achieve comparable accuracy, consistent with the fact that the phase-field dynamics evolve a single scalar order parameter, making direct macroscopic prediction a lower-dimensional task. We emphasize that our goal is not to claim that kinetic learning universally outperforms macroscopic models but rather to demonstrate an alternative route for accelerating LBM simulations. Since a full LBM update requires reconstructing the PDFs via $f = f^{\mathrm{eq}} + f^{\mathrm{neq}}$, missing macroscopic information can easily lead to an ill-posed recovery of the non-equilibrium component. Directly learning the forward evolution of the PDFs provides a more reliable way to preserve non-equilibrium effects and a faithful surrogate for the kinetic LBM operator. Across all cases, despite the larger memory footprint, learning the kinetic fields provides a richer and more physically consistent representation of the LBM dynamics, and serves as a necessary foundation whenever accurate PDF recovery or kinetic-level acceleration is required.

As a result of this discussion of the reviewer's comments we have added the following sentences to the conclusion on Page 17:

"Nevertheless, across the benchmark cases, our comparison shows that kinetic learning provides clear benefits in D3Q19 systems, where the richer PDF representation captures internal correlations that improve macroscopic accuracy, while yielding comparable performance to macroscopic only models in the D2Q9 phase field case. Despite the higher memory cost, directly learning the PDF evolution remains a reliable and physically consistent route for accelerating lattice Boltzmann simulations, as only the kinetic formulation preserves the non equilibrium in-

formation that cannot be recovered from macroscopic simulations variables when higher order moment information is missing."

We have also added a description regarding the scope of our paper in the Introduction Page 6:

"The purpose of these three examples is to demonstrate that the proposed framework applies consistently across distinct LBE formulations and collision models, rather than to suggest that the simulated flows lie outside the continuum regime."

We have added sentences in the Conclusion (See Page 17) to acknowledge that the current examples are in regimes where continuum descriptions apply, but that our contribution is an accelerated, LBE-level surrogate that remains agnostic to the choice of collision operator and can therefore be reused across a broad class of LBM applications:

"Although the examples considered here lie within regimes where continuum descriptions are valid, our contribution is to provide an accelerated, LBE-level surrogate that remains agnostic to the specific collision operator. This allows the learned kinetic update to be reused across a broad class of LBM applications without requiring model-specific reformulation."

R2.5: Practical aspects - data cost, reusability, transferability

The reviewer requests more details on (i) the computational cost of generating training data, (ii) how reusable the trained models are, and (iii) whether the approach can transfer to other geometries or flow configurations.

Response to R2.5

We thank the reviewer for raising the important point regarding data We thank the reviewer for highlighting the practical aspects of data generation, model reusability, and transferability. In this work, our focus is to demonstrate that a neural operator can learn large-step temporal evolution of LBE distribution functions within representative flow configurations. Each dataset is generated using conventional LBM solvers, and the computational cost scales with the spatial resolution and the size of the temporal jump. **We have added the preparation of the generation of training data and the computational cost accordingly in the supplementary information S5.** While the model trained on a given family of geometries and flow conditions can be reused for cases within the same configuration family, transferring it to significantly different geometries or flow regimes would require additional data preparation and extended training datasets.

To clarify this point, we have added the following sentence in the Conclusion Page 17, noting that extending the framework to more diverse geometries and configurations will require additional curated datasets and is an important future research direction for developing generalisable neural operator models.

"...while we have successfully applied the neural-operator architecture to all the LBE problems discussed in this work, extending the framework to a wider variety of geometries and flow configurations will require additional data preparation and larger curated datasets. Building toward such a broader generalisation represents an important future direction, aligned with our longer-term goal of developing a generalisable neural operator for LBM that can transfer across diverse kinetic scenarios. Another promising route is to use ML-based operators as accelerated temporal jump solvers within an LB solver framework, where the ML model advances the solution over large time steps while LB solver provides fine-scale corrections."

Response to Reviewer #3

We thank the reviewer for their thoughtful and constructive comments. Below we address each point in detail.

R3.1 Hardware comparison fairness

Reviewer comment: *In S3, the LB simulations with the C++ code were run on the 1.2 GHz Quad-Core Intel Core I7 but the machine learning inference part was done on a NVIDIA 4090 24GB card. That is, the comparisons were based on different hardware. It is unclear whether the LB simulations were using a single-core or multiple cores. However, it is not unusual for LB simulations to be run hundreds of times faster on a GPU card like the 4090 compared to on a single CPU core. Therefore, it is suggested that comparisons be conducted using the same hardware so that more insights into the performance could be obtained. It would be very useful if the memory consumption could also be compared.*

Response: We thank the reviewer for raising this point. To ensure clarity and full hardware transparency, we have updated the performance assessment to reflect the exact computational setup used in this study. All models were trained on an NVIDIA A100 80 GB GPU, and the originally reported timings corresponded to inference executed on our Intel(R) Xeon(R) Gold 6254 CPU @ 3.10 GHz. For consistency, we have rerun all benchmarks on the same CPU using single-precision (FP32) inference, providing a uniform and fair basis for comparison. The revised manuscript now includes a fully hardware-consistent performance table with the updated timing results.

In addition, we have added a new table in the Supplementary Information (Page 41) detailing the full LBM simulation domain sizes and the effective domain sizes used during training.

**Examples**	Domain size	$T_{\mathbf{LB}}^{\mathbf{total}}$	Effective Domain size	$T_{\mathbf{LB}}^{\mathbf{eff}}$
Kármán vortex street LR (c++)	$110 \times 80 \times 3$ LU	17.24 ± 1.8s	80×80 LU	4.18 ± 0.5s
Liquid ligament breakup (c++)	$48 \times 128 \times 48$ LU	14.88 ± 1.5s	128×48 LU	0.31 ± 0.03s
Bubble adhesion in phase space	100×300 LU	104.34 ± 3.4s	84×78 LU	22.79 ± 0.74s

Table 1: Comparison of full-domain and effective-domain LB simulation runtimes for the three benchmark examples. All LB simulations were executed in single precision (FP32) on the Intel(R) Xeon(R) Gold 6254 CPU and repeated ten times; reported values denote the mean ± standard deviation. $T_{\mathrm{LB}}^{\mathrm{total}}$ corresponds to the runtime of the full-domain LB simulation, while $T_{\mathrm{LB}}^{\mathrm{eff}}$ denotes the runtime when restricting computation to the effective domain. Domain sizes are reported in lattice units (LU).

We have added a speed-up table to the main manuscript (Page 30):

**Examples**	**Simulation time (CPU)**	**Prediction time (CPU)**	**Acceleration**
Kármán vortex street LR (c++)	4.18 ± 0.5s	$0.025 \pm 0.0012s$	≈ 167.2 times
Liquid ligament breakup (c++)	0.31 ± 0.03s	0.043 ± 0.0011s	≈ 7.2 times
Bubble adhesion in phase space	22.79 ± 0.74s	$0.043 \pm 0.0018s$	≈ 530.08 times

Table 2: Comparison of simulation and prediction runtimes. All LB simulations were measured in single precision (FP32) and repeated ten times; the reported values denote the mean, with the accompanying "±" indicating the standard deviation. All ML predictions were also evaluated in FP32 and repeated one hundred times, with mean and standard deviation reported in the same format. The precise definition of the acceleration metric is provided in Supplementary Information S3.

R3.2 Numerical precision

Reviewer comment: *Which precision was used for the float number in the LB simulations and the machine learning part?*

Response: We thank the reviewer for raising this point. In the original submission, the LBM solver was executed in double precision, while the stored simulation data used for training and inference was converted to single precision (FP32). Moreover, our UNO implementation supports only single-precision inference, which is the standard setting for neural-operator models. To ensure a fully fair and hardware-consistent acceleration comparison, we have rerun all LBM simulations in single precision (FP32), matching the precision enforced by the ML surrogate.

All performance tables in the revised manuscript now reflect this uniform FP32–FP32 comparison between the LBM solver and the ML inference.

The updated results, including the revised LBM timings under single precision, are provided in the Supplementary Information (Page 41), and are displayed in Table 1 above.

R3.3 Implementation details and workflow

Reviewer comment: *How was machine learning framework implemented? It is suggested to introduce the software stack used, list some pseudo codes for key steps, and include a flowchart for the workflow in supplementary information.*

Response: We thank the reviewer for this constructive suggestion. Our machine-learning framework is implemented in PyTorch, using Fourier Neural Operators and U-shaped Neural Operators as the backbone architectures. In response to the reviewer's feedback, we have added a concise description of the software stack, a pseudo-code summary of the training procedure (three boxes below) to the Supplementary Information to enhance clarity and reproducibility. These additions can be found in Supplementary Information Section S4.3 Page 17:

We have accordingly documented our full training procedure, structured into three phases.

Phase 1: Data Preparation

Input: LBM dataset $\mathcal{D}$ with distribution fields $f_i(\mathbf{x}, t)$
Goal: Construct input-target training pairs for operator learning

1. Split $\mathcal{D}$ along the temporal axis into train/validation/test.
2. For each split, slide a temporal window of size $S+T$.
3. Construct:
$$X = \{f_{t-S+1}, \ldots, f_t\}, \qquad Y = \{f_{t+1}, \ldots, f_{t+P}\}.$$
4. Stack S distribution fields along the channel dimension $\Rightarrow X \in \mathbb{R}^{(19S)\times H\times W}$.
5. Save datasets into PyTorch `Dataset/DataLoader`.

Phase 2: Model & Loss Setup

1. Initialize UNO model f_θ with:

$$\text{input channels} = 19S, \quad \text{output channels} = 19P.$$

2. Define losses:

   - Data fidelity: $\mathcal{L}_{\text{L2}}$, $\mathcal{L}_{\text{H1}}$
   - Kinetic constraints: density $\mathcal{L}_\rho$, velocity $\mathcal{L}_u$
   - Global equivariance (D4 symmetry for example):

$$\mathcal{L}_{\text{eq}} = \frac{1}{8} \sum_{g \in D_4} \|f_\theta(g(X)) - g(Y)\|_2.$$

3. Set weighted total loss:

$$\mathcal{L} = a\,\mathcal{L}_{\text{L2}} + b\,\mathcal{L}_{\text{H1}} + c\,\mathcal{L}_\rho + d\,\mathcal{L}_u + e\,\mathcal{L}_{\text{eq}}.$$

4. Initialize Adam optimizer with cyclic learning-rate schedule.

Phase 3: Training Loop

For epoch $i = 1 \dots Epochs$:

1. Select learning rate from the periodic schedule.
2. Set model to training mode.
3. For each minibatch (X, Y):
   (a) Move tensors to GPU (FP32).
   (b) Compute prediction: $\hat{Y} = f_\theta(X)$.
   (c) Evaluate all loss components.
   (d) Compute total loss $\mathcal{L}$.
   (e) Backpropagate, clip gradients, optimizer step.
4. Set model to evaluation mode.
5. Compute validation losses over all batches.

R3.4 Training resources

Reviewer comment: *How many resources were used for the training? These could include the time used for the training and the actual memory consumed on A100.*

Response: We thank the reviewer for this question. All models were trained on a single

NVIDIA A100 80 GB GPU. We will include both the peak GPU memory usage and the total training time for each model in the revised Supplementary Information for completeness and reproducibility.

We have added the following text and table in the Supplementary Information Section S3 page 12:

The memory usage reported in Table 3 reflects the peak device allocation observed during training. FNO models require substantially more GPU memory due to their global spectral convolution layers, which scale with the full spatial resolution of the domain. In contrast, UNO employs localized upsampling–downsampling operations, resulting in a significantly smaller memory footprint. Differences across the three cases arise from variations in grid resolution (D3Q19 vs. D2Q9) and the number of velocity channels involved in each lattice model. Training times follow the same trend, with UNO consistently faster to train than FNO owing to its reduced computational and memory demands.

Table 3: GPU memory usage during training of the FNO and UNO models on a single NVIDIA A100 (80 GB) GPU. All models were trained in single precision (FP32) with batch size 32 for 100 epochs.

**Case**	**Model**	**GPU Memory (GB)**	**Training time (hh:mm:ss)**
Kármán vortex street (D3Q19)	FNO	39.9	03:20:16
	UNO	15.4	01:35:32
Liquid ligament breakup (D3Q19)	FNO	38.7	01:00:57
	UNO	15.9	00:26:50
Bubble adhesion (D2Q9)	FNO	29.1	00:15:34
	UNO	17.7	00:13:09

R3.5 Acceleration differences for the ligament case

Reviewer comment: *It appears that acceleration ratio for the liquid ligament breakup case is significantly different from the other two 2D cases. What could be the reason?*

Response: We thank the reviewer for highlighting this point. The lower acceleration ratio observed for the liquid ligament breakup case is mainly due to the smaller temporal jump used in our kinetic scheme due to sharp changes at the interface. For this case, we employ a more conservative time coarsening with $\Delta t = 10$, i.e. each neural operator step advances the solution by 10 underlying LBM time steps, whereas larger temporal jumps are used in the other 2D test cases. We have clarified this in the revised manuscript Section Application, Ligament breakup Page 14:

"The lower acceleration observed in the ligament-breakup case arises from the smaller temporal jump used ($\Delta t = 10$); increasing Δt yields proportionally higher acceleration."

R3.6 Future synergy between LB and ML

Reviewer comment: *It will be great to have a discussion on how the "traditional" LB simulations and the machine learning counterpart could work together in the future. Apart from*

the potentially faster predictions, are there any other benefits by using the machine learning framework?

Response: We appreciate the reviewer's insightful question regarding how traditional LBM solvers and machine-learning-based counterparts may complement one another in the future. Our current study focuses on demonstrating that neural operators can learn kinetic behaviour directly from the LBE formulation, providing a fast-forward surrogate for time evolution. We envision several directions in which LBM and ML can work synergistically. First, traditional LBM solvers can be used to generate high-fidelity training data across parameter ranges that are expensive to explore, enabling ML models to rapidly interpolate or extrapolate within this space. Second, ML-based kinetic solvers may serve as efficient "predictive accelerators" that replace or augment parts of the LBM time-stepping while the remainder of the simulation (e.g., boundary conditions, multiphase coupling, collision terms) continues to rely on the deterministic LBM solver. Finally, ML models offer the potential to learn closure terms, improve subgrid modelling, or correct numerical errors, thereby enhancing the fidelity of conventional LBM simulations.

A more comprehensive integration of ML and LBM, including hybrid solvers, adaptive correction models, and multi-resolution coupling, is an exciting future research direction beyond the scope of the present work, and we plan to explore these synergistic strategies in follow-up studies.

We have inserted this in the revised manuscript conclusion section Page 18:

"A promising hybrid direction is to use ML-based operators as accelerated temporal jump solvers within an LB solver framework, where the ML model advances the solution over large time steps while LB solver provides fine-scale corrections."

R3.7 Other neural operator choices

Reviewer comment: *Apart from UNO and FNO used in this work, is there other neural operator for choice?*

Response: We thank the reviewer for this helpful question. In addition to the FNO and UNO architectures explored in this work, a broad family of neural operators is available for modelling PDE-driven dynamics. Examples include Deep Operator Networks (DeepONets) as well as Tensorized Fourier neural operators or Laplace Neural operators could be used.

Our focus in this study is to demonstrate the feasibility of learning kinetic behaviour directly from the lattice Boltzmann equation using two representative operator classes, spectral (FNO) and upsampling-based (UNO). Extending our approach to other operator families is a promising future direction and may further improve generality and performance, depending on the targeted flow regime.

We have added the following text in the conclusion section Page 17:

"Beyond these neural operators, our framework is readily extendable to other neural operator families, offering a promising avenue for future development."

R3.8 Hyperparameter selection

Reviewer comment: *A discussion on how to choose the hyperparameter for the loss functon(i.e., those listed in Table A1) will be very helpful. What will be the impact on the results if*

another set of hyperparameters are chosen?

Response: We thank the reviewer for this insightful question. The loss-function weights in Table A1 were not chosen as arbitrary initial guesses, but rather to reflect the relative importance of different physical quantities in each problem. In particular, when accurate conservation of density, velocity, or phase-field variables is prioritised, the corresponding loss terms are assigned larger weights to enforce these physical constraints more strongly during training.

Other than that, we did not further perform exhaustive hyperparameter exploration; instead, all models were trained for the same number of epochs using a consistent weighting strategy to provide a systematic and reproducible baseline. This design choice avoids case-specific fine tuning and allows a fair comparison across different LBE models. We note that alternative choices of loss weights can shift the balance between accuracy in kinetic distributions and macroscopic quantities, and interested researchers may further adjust these parameters to emphasise specific physical targets as needed.

Accordingly, we have added additional text in Supplementary Information Section S3, Page 11: "In this work, we adopt a fixed and physically motivated set of hyperparameters for all experiments. The loss-function weights are chosen to reflect the relative importance of key physical quantities (e.g., density, velocity, and phase-field variables) in each problem, thereby enforcing the corresponding conservation or interface constraints during training. All models are trained for the same number of epochs using a consistent weighting strategy, avoiding case-specific fine tuning and ensuring a systematic and fully reproducible benchmark configuration. While we do not perform exhaustive hyperparameter optimisation, the framework allows interested users to further adjust the loss weights to emphasise specific physical targets or accuracy metrics as needed."

R3.9 Typographical corrections

Reviewer comment: *At Page 16 of the supplementary information, "Table VI to Table VIII" was cited which probably should be Table S6 to Table S8. Table S4 and Table S5 appear not cited in the text.*

Response: We thank the reviewer for pointing this out; we have adjusted the text accordingly.

Response to Reviewer #2, Round 2

We thank Reviewer #2 for their continued engagement with our manuscript and for clarifying the remaining concerns. We also appreciate that the reviewer considers the revised organization and the split between the main text and Supplementary Information which we implemented to be more appropriate. In this response, we focus only on the remaining substantive issues. In both the main manuscript and the Supplementary Information, blue text denotes revisions made in round 1, while red text denotes the additional revisions made in round 2. We have revised the manuscript and Supplementary Information in a systematic, point-by-point manner, matching each response below to the manuscript location where the corresponding change was implemented so that the context remains continuous and the terminology is consistent.

Reviewer #2 (Remarks to the Author)

Before addressing the individual points, we would like to clarify the scope of the manuscript. The reviewer raises two important alternative routes: implicit kinetic time integration and macroscopic closure/correction learning. We agree that both are relevant directions. However, neither of them is the focus of this work. The present manuscript addresses a different problem: whether a neural operator can learn a reusable, large-step surrogate for the kinetic LB state, i.e. the particle distribution functions, so that inference can advance the LB state without resolving every intermediate stream–collide step. We have revised the manuscript to make this distinction explicit and to avoid any wording that suggests implicit solvers or closure models are irrelevant.

R2.1: Relationship between LB, BGK, and implicit kinetic treatments

I am still not convinced that the Lattice Boltzmann equation is a special case that can be sufficiently separated from the implicit treatments used in the other models mentioned here (e.g. Boltzmann, Fokker-Planck, and BGK). In particular, the Lattice Boltzmann model and the BGK model are solving the same underlying equation. While the BGK paper suggested in my review is recent contains a method for implicit solution of the BGK model, I provided it more as an example than for its novelty; implicit solution of BGK has been known in the community for some time and it's not clear what separates Lattice Boltzmann from this. The discrete-velocity nature of it is just a numerical approximation. To relate to a different field, this is similar to how e.g. discrete ordinates are used in the neutron or photon transport equations, where there is also extensive literature on implicit time-stepping (I did not make this connection in the original review as the collision operator is different, but the approximation technique is the same).

I do not feel that the statements that these types of techniques may not apply to LBM are valid, or at least require a different justification in the text. The time step restriction is a crucial motivation for this entire work, which seeks to bridge the gap and make predictions beyond the

CFL restriction. I am not convinced that they can be dismissed, even if implicit solution of LBE is not the focus of this paper. Some discussion about why the LBE cannot be solved implicitly needs to be in this paper.

Response to R2.1

We thank the reviewer for returning to this point. We agree that our previous claim may have led to confusion regarding the relationship between implicit kinetic methods and explicitly solving the Lattice Boltzmann equation. To clarify, we regard implicit BGK, Fokker–Planck, Lenard–Bernstein, and discrete-ordinate transport schemes as relevant background for the present work. The LB equation is a discrete-velocity kinetic equation, and implicit treatments of related kinetic equations therefore provide important context. We have removed the statement that such methods "do not directly apply" to LB (Section I, p. 3). However, our objective is not to compare implicit and explicit kinetic time integrators, but to learn a pre-trained multi-step surrogate that replaces the standard LB kinetic update.

The revised manuscript contained herein further clarifies the scope of the contribution. Implicit kinetic schemes and the proposed method act at different algorithmic levels. Implicit schemes modify the time-stepping loop and trade the local stream–collide update for a coupled solver in order to improve stability or stiffness treatment. However, our neural operator is *not* another implicit time integrator. It is a pre-trained multi-step kinetic surrogate for the standard local LB update: once trained, it maps the current PDF state directly to a later PDF state in one forward pass. Thus, the method targets repeated use of the kinetic update, rather than the construction of a new implicit discretization of the LB equation.

This distinction matters because the mainstream LB methodology, including widely used open-source solvers such as OpenLB, Palabos, and HemeLB, is built around the local stream–collide update. In standard LB, collision is local, streaming is an exact shift along $\mathbf{c}_i$, and the update is naturally parallel across both CPU and GPU implementations. Boundary conditions, forcing terms, multiphase couplings, and turbulence closures are also formulated around this local update. A globally coupled implicit LB solve is possible and relevant, but it changes the time-integration route and the algorithmic object rather than accelerating the standard local LB workflow targeted here. Our surrogate is therefore intended to accelerate the kinetic update used by existing LB solvers without any need for them to become implicit solvers.

The revised text reads (Section Introduction, p. 3):

"Implicit and semi-implicit treatments of kinetic equations, including penalty methods for the Boltzmann equation, implicit Fokker–Planck formulations, implicit BGK and Lenard–Bernstein solvers, and implicit discrete-ordinate transport schemes form an important line of related work. These methods modify the time-stepping loop to improve stability or stiffness treatment. However, the focus of the present work is different: we learn a pre-trained multi-step kinetic surrogate for the standard local LB update. Once trained, the neural operator maps the current distribution-function state directly to a later distribution-function state in one forward pass, while preserving the kinetic variables used by conventional LB solvers. Thus, implicit kinetic solvers and the proposed surrogate are complementary rather than competing: the former address how to discretise and solve the kinetic equation within each time step, whereas the latter learns a reusable forward map for the kinetic state generated by standard LB simulations."

We believe this revised text addresses the reviewer's concern without turning the manuscript into a comparison between implicit and explicit LB solvers. It acknowledges the implicit kinetic literature, removes the dismissive wording, and clarifies that our work is about a learned multi-step kinetic map for standard LB workflows.

R2.2: Why learn PDFs rather than stress, viscosity, or closure quantities?

While it is correct to say that the LBE does allows you to capture flow features that a pure hydrodynamic solver cannot, I am still not convinced that the PDF is the correct thing to learn. The LBE is providing closure information for the Navier-Stokes equations, e.g. stress, viscosity, etc. Would it not be simpler to learn a model for those quantities from the LBE data rather than the full PDF? It may also be more transferable to other simulations.

Perhaps a better way to motivate the use of these models would be a rewrite the new text that you added to say instead "a hydro model advances the solution over large time steps while LBE-derived ML model provides fine-scale corrections."

Response to R2.2

We thank the reviewer for raising this point again. We note that this concern is closely related to questions raised in round 1 (Reviewer 1's R1.1 / R1.4 and the present reviewer's own R2.4). In response to those, the revised main manuscript already contains a direct, three-way comparison between (i) kinetic learning with the standard MSE loss, (ii) kinetic learning with the global-symmetry physics-informed loss, and (iii) macroscopic-only learning of the relevant hydrodynamic field, evaluated across all three benchmark cases with ten independently trained models per configuration. For convenience we reproduce those comparisons here.

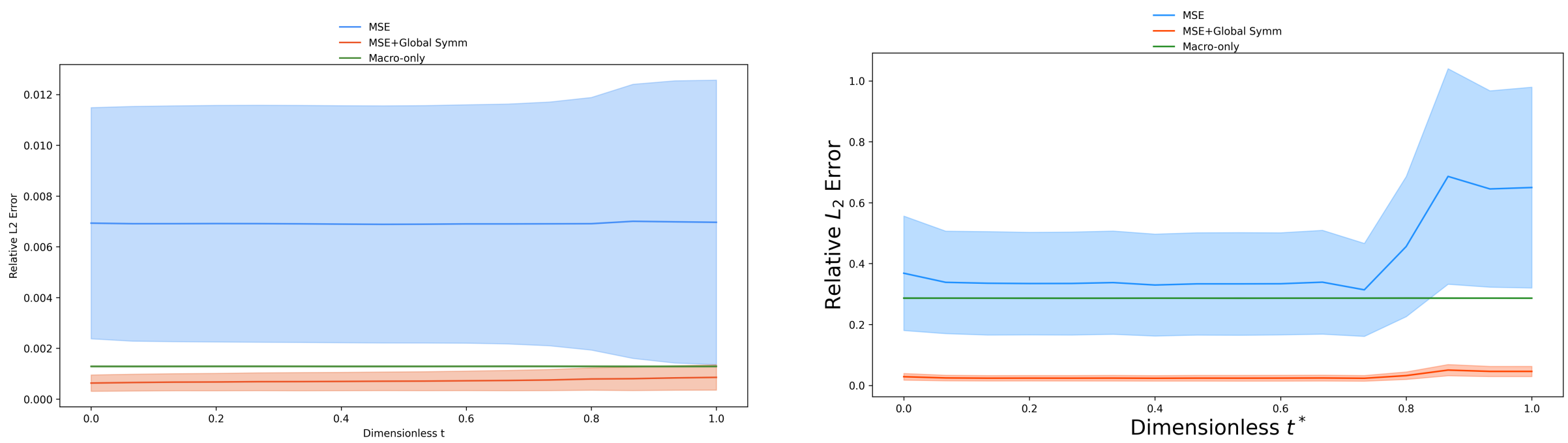

Figure 1: Von Kármán vortex street (D3Q19, BGK with Smagorinsky-type subgrid closure). Per-snapshot relative L_2 error of density (left) and velocity magnitude (right) for three training strategies that share the same architecture, training data, and optimizer: macroscopic-only learning (green), kinetic learning with standard MSE loss (blue), and kinetic learning with the global-symmetry physics-informed loss (orange). Solid lines and shaded bands report the mean and one standard deviation over ten independently trained models. The kinetic models give substantially better velocity prediction, with the gap widening at long rollout times. This comparison was added to the round 1 revised main manuscript as Fig. 3c.

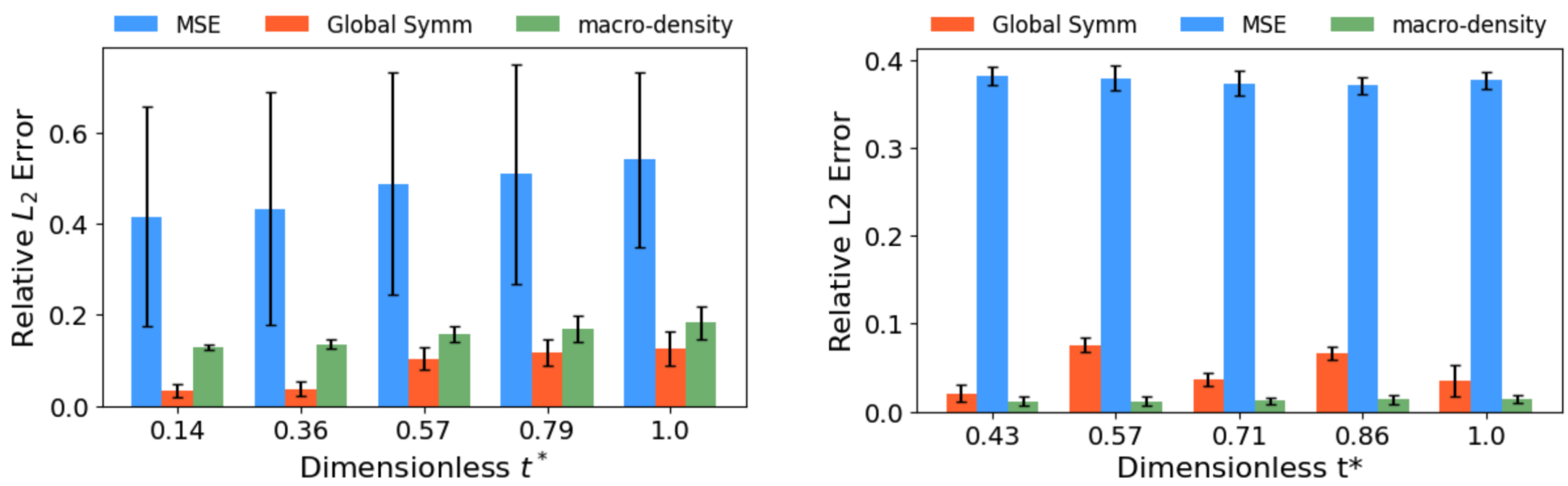

Figure 2: Two-phase test cases. Left: liquid ligament breakup (D3Q19, multiphase pseudopotential model), relative L_2 error of the reconstructed density field at five dimensionless times $t^* = 0.14, 0.36, 0.57, 0.79, 1.0$. Right: bubble adhesion in the presence of a cylinder (D2Q9, phase-field LB), relative L_2 error of the phase-field variable ϕ at five dimensionless times. The same three training strategies as in Fig. 1 are compared, with bars and shaded bands denoting one standard deviation over ten independently trained models. The two panels correspond to Fig. 4c and Fig. 5c of the round 1 revised main manuscript.

These comparisons support a more qualified claim than "PDF prediction is universally more accurate". The relative advantage depends on the observable and on the role the model is intended to play. In the kinetic-dominated von Kármán case, the kinetic models are more stable than the macroscopic-only baseline, especially for velocity at long rollout times. In the ligament-breakup case, the kinetic model with the global-symmetry constraint gives the lowest error and variance, while the macroscopic-density baseline remains competitive because density is the primary reported field in this multiphase example. In the bubble-adhesion case, the lower-dimensional macroscopic phase-field baseline is slightly more accurate on ϕ alone. Our reason for retaining the PDF-level formulation is not that it is always the lowest-error representation for every scalar observable, but because it preserves the full kinetic state needed for a surrogate LB solver. The same observation is made in the round 1 reply to R1.1 and is now reflected in the conclusion of the main manuscript. (Section Conclusion, p. 17)

Beyond the case-by-case accuracy comparisons, we would like to highlight two more fundamental reasons that motivate learning the PDFs rather than a closure quantity. We expand on each below because we believe they were not fully made in the previous round.

Native variable of the LB equation. The PDFs $f_i(\mathbf{x}, t)$ are the state variables of the LB equation; macroscopic density, velocity, stress, and effective viscosity are derived as moments of f_i. Reconstructing f_i from a small set of low-order macroscopic moments is, conversely, a non-unique inverse problem: low-order moments do not pin down the non-equilibrium and higher-order kinetic modes that participate in the next collision step. This motivation is stated in the revised manuscript (Section Introduction, p. 3).

Transferability is not a free lunch for closure learning either. The reviewer remarks that a closure-level model "may also be more transferable to other simulations." We agree that transferability is desirable, but transferability of *learned closures* remains a long-standing open problem in fluid mechanics. Subgrid-scale closures (Smagorinsky, dynamic Smagorinsky, Vre-

man) and RANS closures are well known to be regime-specific: they require recalibration across Reynolds-number ranges, between wall-bounded and free-shear flows, and between Newtonian and non-Newtonian regimes. Our PDF-level surrogate is not magically transferable across regimes either, but its transferability is not constrained by the validity range of any specific closure ansatz, because it is trained on the native PDE variable of the LB equation rather than on the residual of a closure model.

We now turn to the reviewer's specific alternative motivation, namely that "a hydro model advances the solution over large time steps while an LBE-derived ML model provides fine-scale corrections." We agree this is a useful direction. We would, however, like to highlight two practical considerations which led us to avoid adopting it in our work.

Issue 1 – Self-consistency and accuracy. A correction model of this form still relies on LBE simulations as the source of truth used to train the corrector. Once LBE is accepted as the trusted reference, learning a forward map directly on the LBE state f_i is the shorter and more self-consistent route, since it removes the intermediate hydrodynamic solver from the inference loop. The accuracy ceiling of the hydro+correction route is also not automatically higher: the comparisons embedded above (Figs. 1 and 2, reproduced from main-text Figs. 3c, 4c, 5c) show that in the D3Q19 von Kármán and ligament cases, kinetic learning **matches or outperforms macroscopic-only** learning of the same hydrodynamic field, particularly for velocity at long rollout times.

Issue 2 – Information loss in the inference loop. A hydro+correction scheme must, at every prediction step, project the kinetic state down to the macroscopic variables that the hydro solver evolves and, at the next step, reconstruct a full set of distribution functions before LB collision, streaming, boundary, or forcing operations can be applied. The second projection is intrinsically non-unique: the low-order macroscopic moments retained by the hydro solver do not determine the non-equilibrium and higher-order kinetic modes that participate in the subsequent collision step. This physical motivation is stated in the revised manuscript (Section Introduction, p. 3). As a consequence, the inference pipeline carries an irreducible reconstruction error at every macroscopic-to-kinetic step, which compounds over the rollout. By contrast, our PDF-level surrogate operates entirely in f_i-space and introduces only a single PDF$\to$PDF operator error per inference step, with no projection/reprojection loop.

Two complementary paths, neither replaces the other. Hydro+ML correction targets the closure of macroscopic equations using LBE data and naturally extends the LES and RANS closure-learning literature; PDF-level surrogacy targets the kinetic forward map of the LB equation itself and remains compatible with the standard LB workflow described in our R2.1 reply. The two paths address different objectives, and pursuing one does not invalidate the other. We keep the present manuscript focused on PDF-level time advancement but mention the hydro-corrector route as a complementary future direction in the Conclusion (Section Conclusion, p. 17).

The revised text reads (Section Conclusion, p. 17):

"Learning macroscopic closures such as stresses, effective viscosities, or phase-field fluxes is a complementary strategy for hybrid solvers, but it does not replace the kinetic LB state: each subsequent LB update would still require reconstructing the full distribution functions, with

the associated reconstruction error compounding over a rollout. The present work therefore advances the kinetic state directly, from which macroscopic and closure-related quantities can be recovered by moment operations. Using LB-derived data to train macroscopic closure or correction models for hydrodynamic solvers remains a valuable but distinct learning problem."

R2.3: Wall time for data generation, reuse, and a figure of merit

Thank you for the additional setup and dataset information in the supplementary material. However, it is still missing my key concern - what is the wall time needed to generate all of this data, relative to how broadly it might be re-used? As you note, you are somewhat boxed in by the geometries and flow configurations for the extensibility of the ML generated model. Perhaps some figure of merit that shows the time savings of using the ML generated model relative to the cost of the training would better justify this work to a reader.

Response to R2.3

We thank the reviewer for raising this point, which helps us state the practical motivation of the method more precisely. The trained model is not proposed as a way to undercut a single one-off LB simulation, where direct LB integration is more economical, but to convert a one-time offline cost into a reusable forward map that is then queried many times within the same configuration family.

The cumulative LB wall times used to generate the datasets are approximately 9.58 h, 1.82 h, and 14.38 h for the Kármán, ligament, and bubble-adhesion cases respectively, against one-time training costs of 1.59 h, 0.45 h, and 0.22 h. Data generation therefore dominates training in every case (by 6:1 to 65:1), so the offline cost a reader needs to weigh is essentially the dataset cost. The following figure of merit and per-case break-even table have been added (Supplementary Information, Section S3, p. 10):

"To quantify when the offline investment is recovered, we define the break-even number of inference queries as

$$N_{\mathrm{break-even}} = \frac{T_{\mathrm{data}} + T_{\mathrm{train}}}{T_{\mathrm{LB}}^{\mathrm{eff}} - T_{\mathrm{ML}}},$$

where T_{data} is the cumulative wall time used to generate the training dataset, T_{train} is the one-time training time, $T_{\mathrm{LB}}^{\mathrm{eff}}$ is the effective LB runtime for the same prediction window, and T_{ML} is the trained neural-operator inference time. We estimate T_{data} from the mean full-domain LB wall time and the number of simulations or snapshots used for each dataset. Using the timings already reported in the revised Supplementary Information, the resulting estimate is:"

**Case**	T_{data}	T_{train}	$T_{\mathrm{data}} + T_{\mathrm{train}}$	**Saving/query**	$N_{\text{break-even}}$
Kármán vortex street	2000×17.24 s $= 9.58$ h	1.59 h	11.17 h	$4.18 - 0.025 = 4.155$ s	$\approx 9.7 \times 10^3$
Liquid ligament breakup	440×14.88 s $= 1.82$ h	0.45 h	2.27 h	$0.31 - 0.043 = 0.267$ s	$\approx 3.1 \times 10^4$
Bubble adhesion	496×104.34 s $= 14.38$ h	0.22 h	14.60 h	$22.79 - 0.043 = 22.747$ s	$\approx 2.3 \times 10^3$

Table 1: "Approximate offline-cost amortization for the UNO surrogate. T_{data} is estimated from the mean full-domain LB wall time and the number of simulations or snapshots used to generate each dataset. The break-even count gives the number of inference queries after which the saved LB runtime exceeds the one-time data-generation and training cost."

"The break-even count lies between approximately 2.3×10^3 and 3.1×10^4 inference queries

across the three benchmarks; beyond this point the per-query saving accumulates and asymptotically approaches the inference-only acceleration reported in the main text. The relatively higher break-even count for the ligament case stems from its already inexpensive LB baseline (per-query saving of only 0.27 s), so the surrogate's value in that case is better read as enabling stable large-jump interfacial prediction than as a per-query cost reduction." Fig. 3 below visualises the same trade-off as cumulative LB and surrogate wall times in a log–log plot, with the three per-case crossover points marked.

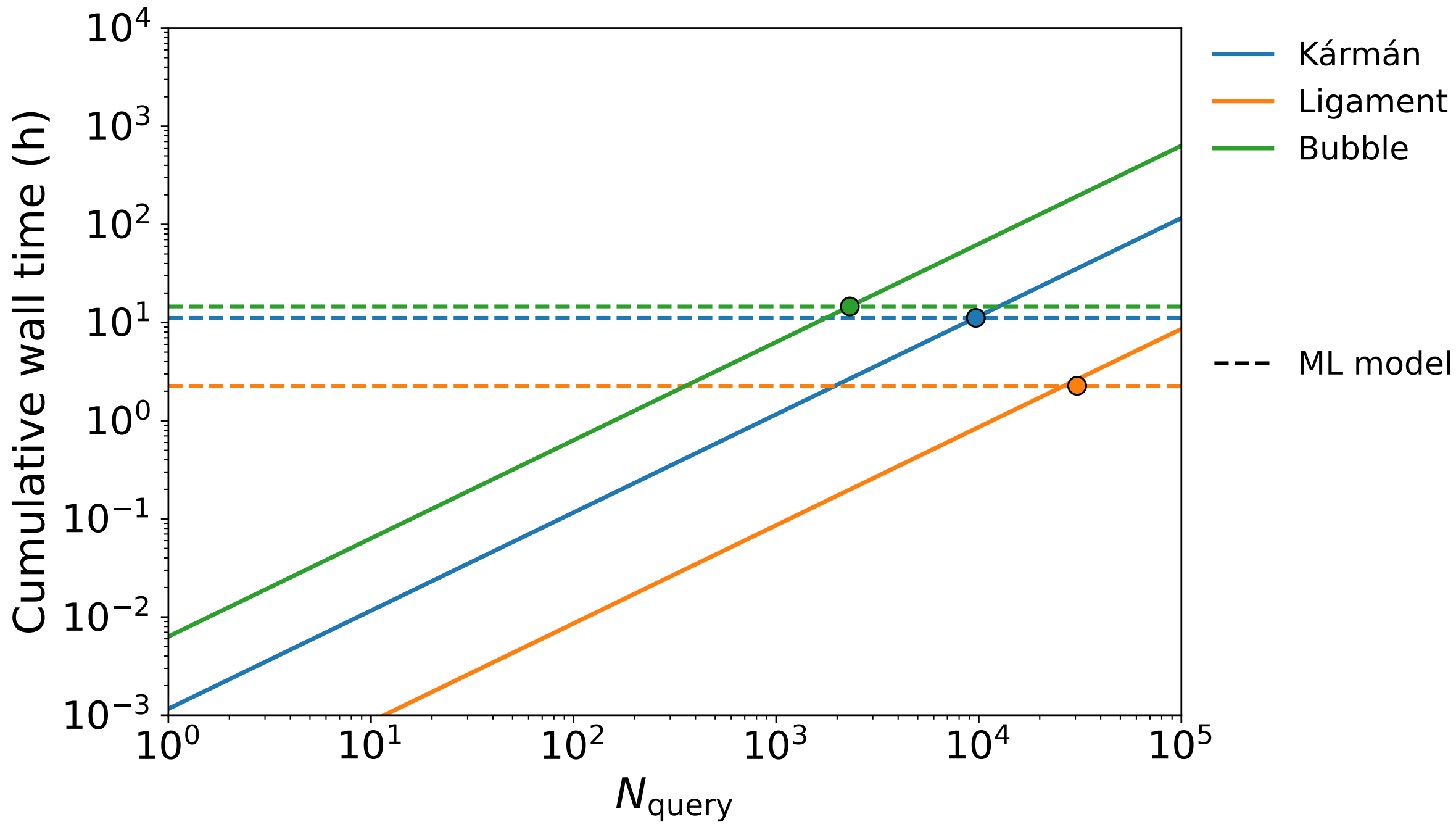

Figure 3: Cumulative wall time versus the number of inference queries N_{query}, in log–log scale. Solid lines are LB baselines (linear in N_{query}); dashed lines are the trained ML model approximated by the one-time offline cost $T_{\text{data}} + T_{\text{train}}$ (the per-query inference time is small enough to be neglected in the visualisation). Crossovers (filled circles) give the per-case break-even count $N_* \approx 9.7 \times 10^3$ (Kármán), 3.1×10^4 (ligament), 2.3×10^3 (bubble) computed from the table above.

The following clarification has also been added (Section Conclusion, p. 17):

"This cost accounting also clarifies the present limitation of the work. The current models are trained per case on relatively narrow geometry and parameter bands, so their reuse window is bounded by those training distributions. Thus, the present figure of merit should be read as a conservative per-case estimate: it answers how many times the surrogate must be reused within the same trained family before the offline cost is justified. A natural and important future direction would be to train a more general LB neural operator across geometries, lattice models, collision kernels and flow regimes. In that setting, the same one-off data-generation and training investment would be spread over far more downstream simulations and inference queries, substantially improving the figure of merit and turning the present per-case demonstration into a more general LB acceleration tool."

REVIEW OF THE PAPER: "FAST FORWARD LATTICE BOLTZMANN: LEARNING KINETIC BEHAVIOUR WITH PHYSICS-INFORMED NEURAL OPERATORS "

This paper presents a physics informed neural operator approach that allows one to step over the time step constraints of the collisional physics in the Lattice Boltzmann method. The neural operator preserves some conservation properties of the underlying LBM as well as rotational equivariance. The authors present numerical examples where their neural operator is able to make predictions for several different scenarios - von Karman vortex shedding, ligament breakup of a spatially varying cylinder, and bubble adhesion to a cylinder.

I have multiple concerns with this manuscript, which I will detail below. I believe this paper has many flaws, and would recommend rejection without major changes in how the material is presented and motivated, and would perhaps be better suited to a different journal.

1. Major comments

(1) This paper is poorly organized. While the authors do an adequate job introducing the LBE equation and giving a brief overview the neural operator $\mathcal{G}$ in Section II, the actual details are not laid down until the end of the paper, after the numerical results and conclusion. The material in Section V should follow the introductory groundwork in Section II. Furthermore, this paper leans heavily on references to multiple Supplementary Material references, which should be used relatively sparingly. Perhaps this paper would be better served as a much longer paper with the supplemental material included.

(2) The primary motivation for the surrogate neural operator G is the time step constraint given by the collisional term in Eq. 1. Indeed, this is an issue that plagues many kinetic representations and several attempts have been made in other fields to address this. However, aside from a comment in the abstract about this neural operator framework being agnostic to the specific form of the underlying collision model, no connections are made to any other kinetic models used in other literature or other attempts to address this issue. For example, for the more complicated Boltzmann collision operator for rarefied gas dynamics, Filbet and Jin (JCP, 2010) present a penalty method that allows for taking large time steps even in collisional regimes.

(3) Building on the previous point, many other collision models admit implicit time stepping of collision operators. For example, see the implicit Fokker-Planck model of Taitano et al (JCP 2015) for the Fokker-Planck collision model. Additionally, for simpler BGK or Lenard-Bernstein models, implicit discretizations of the collision

operator have been used in practice for quite some time. See for example, Coron and Perthame, Siam J. Num. Anal., 1991, or Habbershaw et al, J. Stat. Phys 2025, or the review in https://arxiv.org/pdf/2404.18039

(4) The Boltzmann-BGK equation, which is the underlying equation that the LBM solves, has an asymptotic limit of the Euler or Navier-Stokes equations. This is defined by the Knudsen number, which is typically defined as a ratio of the mean free path between particle collisions and some characteristic length scale. This can also equivalently be defined as a ratio of timescales - the ratio of the average collision time (τ in BGK models) and a characteristic timescale. For a problem where the reference timescale, e.g. the time steps we see, is significantly larger than the collision time, we expect kinetic models to reduce to a solution to the Euler or Navier-Stokes equations, as any interesting kinetic dynamics would have been smoothed out before then. What then makes this method more advantageous than simply solving the hydrodynamics equations? Or, perhaps, training a neural operator on the less expensive hydrodynamic equations. Is there something uniquely kinetic that is being captured in these numerical examples that hydro on its own cannot capture?

(5) For practical takeaways, there should be a longer discussion of how much time is spent to generate data for the model vs how re-usable it is. Furthermore, could information/models learned by this technique be easily transferred to other problems? The application to more complex flows is unclear from what is in the manuscript.

SECOND REVIEW OF THE PAPER: "FAST FORWARD LATTICE BOLTZMANN: LEARNING KINETIC BEHAVIOUR WITH PHYSICS-INFORMED NEURAL OPERATORS "

I appreciate the authors' thoughtful replies to my reviews. I will address this review to the authors' replies to each topic in sequence

(1) Thank you for the reorganization of the paper, and for noting the constraints on the journal format, and I think the split of the content between the 'main' paper and the supplemental is more appropriate.

(2) (combined with the next item)

(3) I am still not convinced that the Lattice Boltzmann equation is a special case that can be sufficiently separated from the implicit treatments used in the other models mentioned here (e.g. Boltzmann, Fokker-Planck, and BGK). In particular, the Lattice Boltzmann model and the BGK model are solving the same underlying equation. While the BGK paper suggested in my review is recent contains a method for implicit solution of the BGK model, I provided it more as an example than for its novelty; implicit solution of BGK has been known in the community for some time and it's not clear what separates Lattice Boltzmann from this. The discrete-velocity nature of it is just a numerical approximation. To relate to a different field, this is similar to how e.g. discrete ordinates are used in the neutron or photon transport equations, where there is also extensive literature on implicit time-stepping (I did not make this connection in the original review as the collision operator is different, but the approximation technique is the same).

    I do not feel that the statements that these types of techniques may not apply to LBM are valid, or at least require a different justification in the text. The time step restriction is a crucial motivation for this entire work, which seeks to bridge the gap and make predictions beyond the CFL restriction. I am not convinced that they can be dismissed, even if implicit solution of LBE is not the focus of this paper. Some discussion about why the LBE cannot be solved implicitly needs to be in this paper.

(4) While it is correct to say that the LBE does allows you to capture flow features that a pure hydrodynamic solver cannot, I am still not convinced that the PDF is the correct thing to learn. The LBE is providing closure information for the Navier-Stokes equations, e.g. stress, viscosity, etc. Would it not be simpler to learn a model for those quantities from the LBE data rather than the full PDF? It may also be more transferable to other simulations

(5) Thank you for the additional setup and dataset information in the supplementary material. However, it is still missing my key concern - what is the wall time needed to generate all of this data, relative to how broadly it might be re-used? As you

note, you are somewhat boxed in by the geometries and flow configurations for the extensibility of the ML generated model. Perhaps some figure of merit that shows the time savings of using the ML generated model relative to the cost of the training would better justify this work to a reader.

Perhaps a better way to motivate the use of these models would be a rewrite the new text that you added to say instead "a hydro model advances the solution over large time steps while LBE-derived ML model provides fine-scale corrections."
